# *p*-Terphenyls From *Aspergillus* sp. GZWMJZ-055: Identification, Derivation, Antioxidant and α-Glycosidase Inhibitory Activities

**DOI:** 10.3389/fmicb.2021.654963

**Published:** 2021-02-25

**Authors:** Yanchao Xu, Yong Wang, Dan Wu, Wenwen He, Liping Wang, Weiming Zhu

**Affiliations:** ^1^State Key Laboratory of Functions and Applications of Medicinal Plants, Guizhou Medical University, Guiyang, China; ^2^School of Pharmaceutical Sciences, Guizhou Medical University, Guiyang, China; ^3^Key Laboratory of Chemistry for Natural Products of Guizhou Province, Chinese Academy of Sciences, Guiyang, China; ^4^Laboratory for Marine Drugs and Bioproducts, Pilot National Laboratory for Marine Science and Technology, School of Medicine and Pharmacy, Ocean University of China, Qingdao, China

**Keywords:** endophytic fungus, *Aspergillus* sp., antioxidant activity, α-glucosidase inhibition, *Eucommia ulmoides*

## Abstract

One new (**1**) and fifteen known (**2**–**16**) *p*-terphenyls were isolated from a solid culture of the endophytic fungus *Aspergillus* sp. GZWMJZ-055 by adding the leaves of its host *Eucommia ulmoides*. Furthermore, nine *p*-terphenyls (**17**–**25**) were synthesized from the main compounds (**5**–**7**), among which derivatives **18**, **19**, **21**, **22**, and **25** are new *p*-terphenyls. Compounds **15** and **16** were also, respectively, synthesized from compounds **6** and **7** by oxidative cyclization of air in the presence of silica gel. These *p*-terphenyls especially those with 4,2′,4″-trihydroxy (**4**–**7**, **20**, **21**) or 4, 4″-dihydroxy-1,2,1′,2′-furan (**15**, **16**) substituted nucleus, exhibited significant antioxidant and α-glucosidase inhibitory activities and lower cytotoxicity to caco-2 cells. The results indicated their potential use as lead compounds or dietary supplements for treating or preventing the diabetes.

## Introduction

Diabetes is a chronic metabolic disease characterized by high blood sugar (HBS). Long-term HBS causes the damage to blood vessels and endangers various organs such as the heart, brain, kidneys, peripheral nerves, and eyes, and thus seriously affects the life quality of patients. Studies show that oxidative stress may be one of the important causes for diabetes and its complications. Too much reactive oxygen species in the body will increase the maturation disorder and apoptosis of pancreatic β-cells, leading to decrease insulin synthesis and secretion. Hyperglycemia and hyperlipidemia in diabetic patients can promote the production of active oxides, causing oxidative stress, then oxidative stress and hyperglycemia promote each other, leading to a vicious circle (Karunakaran and Park, 2013). At present, the treatment of type 2 diabetes is based on oral drugs, mainly containing metformin, α-glucosidase inhibitors, dipeptidyl peptidase IV inhibitors, and sodium-glucose cotransporter 2 inhibitors. Among them, α-glucosidase inhibitors can inhibit the degradation of polysaccharides to glucose and delay the absorption of glucose in the small intestine to reduce blood sugar. Such drugs can effectively reduce postprandial hyperglycemia without causing symptoms of hypoglycemia and are highly beneficial to patients who use carbohydrates as their main source of calories.

The α-glucosidase inhibitors, such as acarbose, miglitol, and voglibose currently used clinically are all microbial metabolites or their derivatives. Therefore, discovery of the new α-glucosidase inhibitors from microbial natural products (NPs) has unique advantages. *p*-Terphenyls, as an important kind of fungal NPs, its chemical investigation could be dated back to 1877 (Liu, 2006). At present, over 230 *p*-terphenyls have been isolated from fungi and lichens (Li et al., 2018). In addition, some *p*-terphenyl derivatives were also total synthesized (Yonezawa et al., 1998; Takahashi et al., 2017; Zhang et al., 2018). As reported, *p*-terphenyls had a broad spectrum of biological properties, such as cytotoxic (Wang et al., 2019, 2020), antimicrobial (Intaraudom et al., 2017), and phosphodiesterase inhibitory (El-Elimat et al., 2013) activities, but the most interesting bioactivities were antioxidative (Kuhnert et al., 2015) and *α*-glucosidase inhibitory activities (Ma et al., 2014). Furthermore, *p*-terphenyls can also be isolated from edible mushroom (Liu et al., 2004; Ma et al., 2014; Wang et al., 2014), indicating that this kind of compounds have low toxicity in the human body and are very suitable for the research of anti-diabetic drugs.

**FIGURE 1:**
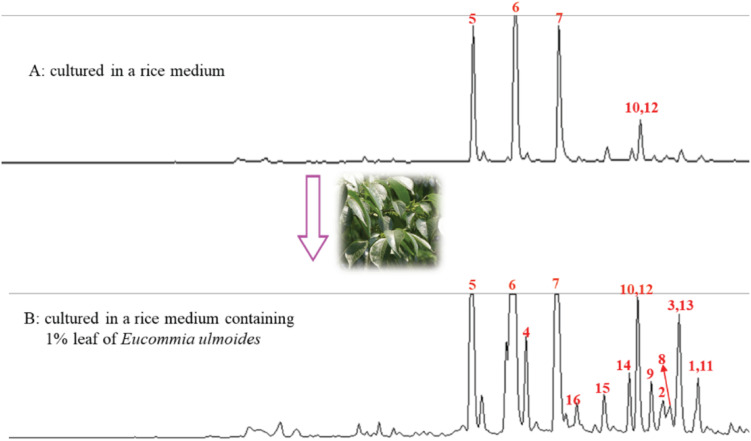
HPLC Analysis of EtOAc extract from the rice cultures without **(A)** and within **(B)** the leaf of *E. ulmoides*.

**FIGURE 2:**
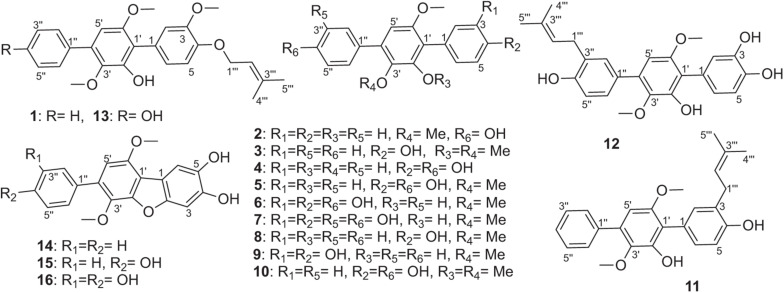
The structures of compounds **1**–**16**.

**SCHEME 1:**
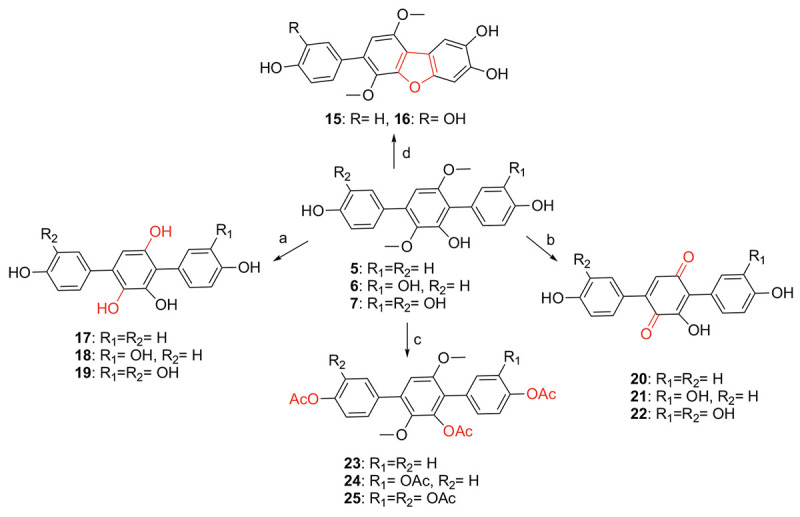
Synthesis of compounds **15**–**25** from **5** to **7** (a. BBr_3_/CH_2_Cl_2_, −20°C to rt; b. BBr_3_/CH_2_Cl_2_, −20°C to rt, then O_2_/silica gel/MeOH; c. Ac_2_O, DMAP, CH_2_Cl_2_, 40°C; d. O_2_/silica gel/MeOH, rt).

During our research for new compounds with α-glucosidase inhibitory activity from the plant endophytes, we isolated and identified a *p*-terphenyls-producing strain, *Aspergillus* sp. GZWMJZ-055 endophytic with the famous Chinese medical plant *Eucommia ulmoides*. The fermentation potency (5.3–9.5 g/kg) and the α-glucosidase inhibitory activity (IC_50_ 15.0 to 2.0 μg/mL) of the ethyl acetate (EtOAc) extracts of the fermentation significantly increased after adding the leaves of *Eucommia ulmoides* to the culture medium (Figure 1). Chemical investigation led to the isolation of sixteen *p*-terphenyls, including the new 3-*O*-methyl-4″-deoxyterprenin (**1**) as well as the known 4-deoxyterphenyllin (**2**) (Lin et al., 2019), 4″-deoxy-2′-methoxyterphenyllin (**3**) (Shan et al., 2019), 5′-methoxy-[1,1′:4′,1″-terphenyl]-2′,3′,4,4″-tetraol (**4**) (Zhang et al., 2018), terphenyllin (**5**) (Kamigauchi et al., 1998), 3-hydroxyterphyllin (**6**) (Liu et al., 2012), 3,3″-dihydroxyterphyllin (**7**) (Liu et al., 2012), 4″-deoxyterphenyllin (**8**) (Shan et al., 2019), 4″-deoxy-3-hydroxyterphenyllin (**9**) (Wang et al., 2020), 3′-*O*-methylterphenyllin (**10**) (Yan et al., 2017), 4″-deoxyprenylterphenyllin (**11**) (Wei et al., 2007), prenylterphenyllin A (**12**) (Cai et al., 2011), 3-methoxyterprenin (**13**) (Kamigauchi et al., 1998), 4″-deoxycandidusin A (**14**) (Guo et al., 2012), candidusin A (**15**) (Wang et al., 2020), and candidusin B (**16**) (Liu et al., 2012; Figure 2). Compounds **15** and **16** were also, respectively, prepared from compounds **6** and **7** by an intramolecular oxidative cyclization of air catalyzed by silica gel (Scheme 1). In addition, nine *p*-terphenyls (**17**–**25**) were synthesized from the main NPs (**5**–**7**) (Scheme 1), among which derivatives **18**, **19**, **21**, **22**, and **25** are new *p*-terphenyls. These *p*-terphenyls showed antioxidant and α-glycosidase inhibitory activities (Table 1).

**TABLE 1:** Antioxidant and α-glycosidase activities of compounds **1**–**25**.

**Compound**	**DPPH (IC_50_, μM)**	**ORAC (μMole TE/μMole)**	**α-glucosidase (IC_50_, μM)**	**α-glucosidase in caco-2 (IC_50_, μM)**
**1**	>100	1.0 ± 0.04	>500	–
**2**	>100	1.2 ± 0.09	239.1 ± 5.9	–
**3**	>100	2.1 ± 0.5	>500	–
**4**	1.7 ± 0.02	6.8 ± 0.05	5.9 ± 0.6	–
**5**	10.9 ± 0.3	6.0 ± 0.09	2.8 ± 0.2	0.38 ± 0.01
**6**	1.4 ± 0.01	5.7 ± 0.3	10.9 ± 1.3	0.29 ± 0.02
**7**	1.1 ± 0.03	4.5 ± 0.4	18.3 ± 0.9	0.36 ± 0.01
**8**	57.8 ± 0.8	0.5 ± 0.01	129.8 ± 3.5	–
**9**	1.9 ± 0.04	0.5 ± 0.06	464.5 ± 23.6	–
**10**	>100	0.5 ± 0.03	>500	–
**11**	1.6 ± 0.01	1.2 ± 0.5	34.4 ± 1.1	–
**12**	1.6 ± 0.02	1.4 ± 0.01	64.4 ± 1.2	–
**13**	>100	0.4 ± 0.07	152.5 ± 9.6	–
**14**	4.0 ± 0.2	0.4 ± 0.06	128.3 ± 2.3	–
**15**	3.6 ± 0.06	2.1 ± 0.06	13.1 ± 0.2	0.11 ± 0.02
**16**	3.2 ± 0.1	2.0 ± 0.06	7.9 ± 0.3	0.36 ± 0.01
**17**	5.3 ± 0.06	4.8 ± 0.3	25.1 ± 0.7	–
**18**	3.7 ± 0.07	3.7 ± 0.1	25.0 ± 0.8	–
**19**	1.8 ± 0.04	2.6 ± 0.1	19.4 ± 0.1	–
**20**	5.9 ± 0.08	6.1 ± 0.2	8.9 ± 0.4	0.11 ± 0.01
**21**	1.6 ± 0.04	3.9 ± 0.1	4.0 ± 0.08	0.12 ± 0.01
**22**	1.7 ± 0.02	2.8 ± 0.3	10.1 ± 0.1	–
**23**	>100	0.1 ± 0.01	>500	–
**24**	37.8 ± 0.1	0.8 ± 0.07	>500	–
**25**	11.0 ± 0.1	1.7 ± 0.07	>500	–
**VC**	2.8 ± 0.03			
**Acarbose**			265.4 ± 10.2	157.7 ± 14.0

*– not tested.*

## Materials and Methods

### General Experimental Procedures

UV spectra were measured on a Waters 2487 dual *λ* absorbance detector. IR spectra were recorded on a Nicolet Nexus 470 spectrophotometer as KBr disks. ^1^H, ^13^C NMR and 2D NMR spectra were recorded on Bruker-600 MHz using TMS as an internal standard. ESIMS and HR-ESIMS analysis were carried out on Waters Xevo TQS and Agilent Technologies 6530 Accurate-Mass Q-TOF LC/MS, respectively. Column chromatography was performed on silica gel (200–300 mesh; Qingdao Puke Parting Materials Co., Ltd., China), Sephadex LH-20 (Amersham Biosciences, Uppsala, Sweden), silica gel H, and plates precoated with silica gel GF254 (Qingdao Puke Parting Materials Co., Ltd., China), respectively. HPLC separation was performed on HITACHI Primaide with an ODS column (YMC-pack ODS-A, 10 mm × 250 mm, 5 μm, 4 mL/min). Synthetic compounds were also purified using a SepaBean machine equipped with SepaFlash columns (Santai Technologies Inc., China).

### Fungal Material

The fungus *Aspergillus* sp. GZWMJZ-055 was isolated from the leaves of *Eucommia ulmoides* collected from Guiyang Medicinal Botanical Garden and was determined as *Aspergillus* sp. by the phylogenetic tree (Supplementary Figure 1) of the ITS sequence (GenBank No. KY038594). The strain was deposited in Guiyang laboratory in 20% glycerol at −80°C.

### Cultural Media

The working strain was prepared on PDA agar medium, containing 20% potato, 2.0% glucose, 2.0% agar, and 1 L tap water. The seed medium contained 2.0% maltose, 2.0% mannitol, 1.0% glucose, 1.0% sodium glutamate, 0.3% yeast extract, 0.1% corn extract, 0.1% KH_2_PO_4_, 0.05% MgSO_4_⋅7H_2_O, 1 L tap water. The solid fermentation medium was prepared from 100 g rice, 1 g dry leaves of *Eucommia ulmoides* and 120 mL distilled water in a 1000-mL Erlenmeyer flask.

### Fermentation, Extraction and Isolation

The fungal strain GZWMJZ-055 was cultured on PDA at 28°C for 3 days to prepare the seed culture. Spores were incubated at 28°C for 2 days, a rotary shaker with shaking at 120 rpm in a 500 mL cylindrical flask containing 150 mL seed medium. The seed medium (5 mL) was added to the above rice fermentation medium in a 1000-mL Erlenmeyer flask. Totally, 100 Erlenmeyer flasks were incubated at room temperature (rt) under static conditions for 30 days. The cultures were then extracted by ethyl acetate (EtOAc) (500 mL for each) three times and the combined EtOAc extracts were dried *in vacuo* until constant weigh to yield 423.5 g of EtOAc extract.

The EtOAc extract (423.5 g, adsorbed in 500 g 100–200 mesh silica gel) was chromatographed on a silica gel (2 kg, 200–300 mesh) column (10 cm × 100 cm) using step gradient elution of CH_2_Cl_2_–MeOH (v/v100:0, 100:1, 50:1, 25:1, 10:1, 5:1, 1:1, 1:2, 1:5, and 1:10, each 8 L) to yield fifteen fractions (Fr.1–Fr.15). Fraction 2 (18.7 g, adsorbed in 50 g 100–200 mesh silica gel) was then subjected to a silica gel (400 g, 200–300 mesh) column (6 cm × 61 cm), eluting with CH_2_Cl_2_–MeOH (100:1, v/v) to afford sixteen subfractions (Fr.2-1∼Fr.2-16). Subfraction Fr.2–3 (1.2 g) was separated with silica gel chromatography eluted by CH_2_Cl_2_ to yield five fractions (Fr.2-3-1∼Fr.2-3-5). Subfraction Fr.2-3-4 (100.6 mg) was purified by semi-preparative HPLC [80% MeOH/H_2_O with 0.15% trifluoroacetic acid (TFA)] to yield compound **1** (17.2 mg, t*_*R*_* 14.0 min). Fraction 2-7 (2.8 g) was further separated into six subfractions by Sephadex LH-20 eluting with MeOH-CH_2_Cl_2_ (1:1, v/v). Subfraction 2-7-4 (455.0 mg) was separated with silica gel chromatography eluted by CH_2_Cl_2_ to yield three fractions (Fr.2-7-4-1∼Fr.2-7-4-3). Subfraction 2-7-5 (58.8 mg) was purified by semi-preparative HPLC (80% MeOH/H_2_O) to yield compound **11** (9.7 mg, t*_*R*_*13.6 min). Fraction 2-9 (185 mg) was further separated into five subfractions (Fr.2-9-1∼Fr.2-9-5) by Sephadex LH-20 eluting with MeOH-CH_2_Cl_2_ (1:1, v/v). Subfraction 2-9-3 separated with silica gel chromatography eluted by CH_2_Cl_2_ to yield five fractions (Fr.2-9-3-1∼Fr.2-9-3-5). Fr.2-9-3-5 was further purified by semi-preparative HPLC (80% MeOH/H_2_O) to yield compound **14** (5.6 mg, t*_*R*_* 7.0 min). Subfraction 2-9-5 was purified by semi-preparative HPLC (70% MeOH/H_2_O) to yield compound **12** (6.0 mg, t*_*R*_* 7.2 min). Fraction 2-13 (240.2 mg) was further separated into fourteen subfractions by Sephadex LH-20 eluting with MeOH-CH_2_Cl_2_ (1:1, v/v). Subfraction 2-13-10 was further separated into four subfractions separated with silica gel chromatography eluted by MeOH-CH_2_Cl_2_ (1:80, v/v). Fr.2-13-10-2 was purified by semi-preparative HPLC (75% MeOH/H_2_O) to yield compound **3** (12.5 mg, t*_*R*_* 10.0 min). Subfraction 2-13-13 was purified by semi-preparative HPLC (70% MeOH/H_2_O) to yield compound **2** (7.1 mg, t*_*R*_* 8.7 min). Fraction 2-14 (452.3 mg) was further separated into six subfractions separated with silica gel chromatography eluted by CH_2_Cl_2_. Fr.2-14-5 (20.6 mg) was purified by semi-preparative HPLC (80% MeOH/H_2_O) to yield compound **13** (6.1 mg, t*_*R*_*16.0 min). Fraction 9 (3.5 g) was subjected to a silica gel column, elution with step gradient elution of CH_2_Cl_2_–MeOH (0–100%, v/v) to afford three subfractions (Fr.9-1∼Fr.9-3). Fraction 9-2 (1.2 g) was further separated into nine subfractions by Sephadex LH-20 eluting with MeOH. Fraction 9-2-3 (418.2 mg) was further separated into seven subfractions separated with silica gel chromatography eluted by MeOH-CH_2_Cl_2_ (1:20, v/v). Fraction 9-2-3-2 (20.0 mg) was purified by HPLC on an ODS column (75% MeOH/H_2_O) to give compound **8** (4.0 mg, t*_*R*_* 8.0 min). Fraction 9-2-3-5 (26.1 mg) was purified by HPLC on an ODS column (75% MeOH/H_2_O) to give compound **9** (6.4 mg, t_*R*_ 4.5 min). Fraction 9-2-9 (29.7 mg) was purified by HPLC on an ODS column (65% MeOH/H_2_O) to give compound **15** (6.1 mg, t*_*R*_* 5.0 min). Fraction 9-3 (1.3 g) was further separated into thirteen subfractions by Sephadex LH-20 eluting with MeOH. Fraction 9-3-7 (317.1 mg) was further separated into nine subfractions separated with silica gel chromatography eluted by MeOH-CH_2_Cl_2_ (1:10, v/v). Fraction 9-3-7-4 (10.7 mg) was purified by HPLC on an ODS column (55% MeOH/H_2_O) to give compound **10** (2.8 mg, t*_*R*_* 18.2 min). Fraction 9-3-8 was further separated with silica gel chromatography eluted by MeOH-CH_2_Cl_2_ (1:15, v/v) to get compound **5** (900 mg). Fraction 9-3-12 (52.7 mg) was purified by HPLC on an ODS column (60% MeOH/H_2_O) to give compounds **4** (6.6 mg, t*_*R*_* 10.4 min) and **16** (6.0 mg, t*_*R*_* 18.0 min). Fraction 10 (2.8 g) was chromatographed on a silica gel column using step gradient elution of CH_2_Cl_2_–MeOH (5–100%, v/v) to yield compounds **6** (700 mg) and **7** (1.2 g).

Compound **1**: white powder; UV (MeOH) *λ*_*max*_ (log *ε*) 230 (4.03), 276 (3.96) nm; IR (KBr) *v*_*max*_ 3486, 2934, 1517, 1482, 1461, 1399, 1238, 1117, 1075, 1009, 770, and 703 cm^–1^; HR ESIMS *m/z* 443.1837 [M+Na]^+^ (calcd. for C_26_H_28_O_5_Na, 443.1829) (Supplementary Figure 2); ^1^H and ^13^C NMR data, see Table 2 and Supplementary Figures 3–7.

**TABLE 2:** ^1^H (600 MHz) and ^13^C (150 MHz) NMR data of compounds **1**, **18**, and **19** in DMSO-*d*_6_.

**Position**	**1**		**18**		**19**	
	**δ _*C*_**	**δ _*H*_ (*J* in Hz)^a^**	**δ _*C*_**	**δ _*H*_ (*J* in Hz)**	**δ _*C*_**	**δ _*H*_ (*J* in Hz)**
1	126.5, C		125.7, C		125.7, C	
2	115.0, CH	6.88, d (1.8)	118.6, CH	6.75, d (2.0)	118.6, CH	6.74, d (2.0)
3	148.2, C		143.8, C		143.7, C	
4	146.7, C		144.3, C		144.2, C	
5	112.5, CH	6.96, d (8.3)	114.8, CH	6.71, d (8.0)	114.8, CH	6.71, d (8.0)
6	123.1, CH	6.82, dd (8.3, 1.8)	122.0, CH	6.60, dd (8.0, 2.0)	122.0, CH	6.59, dd (8.0, 1.9)
1′	117.7, C		116.0, C		115.8, C	
2′	148.3, C		145.0, C		144.9, C	
3′	139.5, C		134.4, C		134.4, C	
4′	132.7, C		129.6, C		130.1, C	
5′	103.2, CH	6.46, s	106.5, CH	6.26, s	106.5, CH	6.24, s
6′	153.2, C		148.2, C		148.1, C	
1″	138.2, C		128.2, C		128.2, C	
2″	128.7, CH	7.61, d (7.5)	130.0, CH	7.35, d (8.5)	116.6, CH	6.97, d (2.0)
3″	128.4, CH	7.47, t (7.5)	114.9, CH	6.79, d (8.5)	144.7, C	
4″	127.3, CH	7.37, t (7.5)	156.2, C		144.2, C	
5″	128.4, CH	7.47, t (7.5)	114.9, CH	6.79, d (8.5)	115.3, CH	6.75, d (8.0)
6″	128.7, CH	7.61, d (7.5)	130.0, CH	7.35, d (8.5)	119.9, CH	6.79, dd (8.0, 2.0)
1″′	64.8, CH_2_	4.53, d (6.7)				
2″′	120.4, CH	5.47, t (6.7)				
3″′	136.9, C					
4″′	18.0, CH_3_	1.72, s				
5″′	25.5, CH_3_	1.76, s				
3-OMe	55.5, CH_3_	3.73, s				
3′-OMe	60.4, CH_3_	3.30, s				
6′-OMe	55.7, CH_3_	3.67, s				
2′-OH		8.68, s				

*^*a*^d, dd, s, t respectively means doublet, a doublet of doublets, singlet and triplet.*

### Chemical Synthesis Procedures

#### Synthesis of Compounds **15** and **16**

Compound **6** (30 mg, 85 μmol) was dissolved in MeOH (10 mL), and 200–300 mesh silica gel (4 g) was then added to the solution. After the reaction was stirred overnight at rt, the solvent was evaporated. The residue was purified by flash column chromatography (FCC) eluting with EtOAc-CH_2_Cl_2_ (v/v 1:5) to give compound **15** (26 mg, 74 μmol, 87% yield) as a light-yellow solid (*R*_*f*_ 0.4). By the same procedure, compound **16** (24 mg, 65 μmol, 80% yield) was prepared from the reaction of compound **7** (30 mg, 81 μmol) and purified as a light-yellow solid (*R*_*f*_ 0.4) by FCC eluting with EtOAc-CH_2_Cl_2_ (v/v 1:3).

Compound **15**: ^1^H NMR (600 MHz, DMSO-*d*_6_) and ^13^C NMR (150 MHz, DMSO-*d*_6_) data, see Supplementary Table 5 and Supplementary Figures 8, 9. ESIMS *m/z* 351.0 [M−H]^–^.

Compound **16**: ^1^H NMR (600 MHz, DMSO-*d*_6_) and ^13^C NMR (150 MHz, DMSO-*d*_6_) data, see Supplementary Table 5 and Supplementary Figures 10, 11. ESIMS *m/z* 367.1 [M−H]^–^.

#### Synthesis of Compounds **17**–**19**

Compound **5** (30 mg, 89 μmol) was dissolved in CH_2_Cl_2_ (5 mL), and BBr_3_ (0.8 mL, 0.54 mmol, 17% in CH_2_Cl_2_) was then added at −20°C under the protection of argon. The reaction mixture was warmed up to rt and stirred overnight. The reaction was quenched by adding H_2_O (20 mL) at 0°C, and EtOAc (200 mL) was then added. The EtOAc phase was washed with H_2_O (20 mL × 4), dried over anhydrous Na_2_SO_4_, and concentrated in vacuo. The residue was purified by semipreparative HPLC eluting with 40% MeOH/H_2_O containing 0.15% TFA to provide compound **17** (26 mg, 84 μmol, 94% yield, *t*_*R*_ 7.5 min) as an orange solid. By the same procedures, compound **18** (25 mg, 77 μmol, 91% yield, *t*_*R*_ 8 min) was prepared from the reaction of compound **6** (30 mg, 85 μmol) and BBr_3_ (0.76 mL, 0.51 mmol) in CH_2_Cl_2_ and purified from HPLC by 30% MeOH/H_2_O containing 0.15% TFA, while compound **19** (25 mg, 73 μmol, 90% yield, *t*_*R*_ 7 min) was prepared from the reaction of **7** (30 mg, 81 μmol) and BBr_3_ (0.72 mL, 0.48 mmol) in CH_2_Cl_2_ and purified from HPLC by 25% MeOH/H_2_O containing 0.15% TFA.

Compound **17**: ^1^H NMR (600 MHz, DMSO-*d*_6_) and ^13^C NMR (150 MHz, DMSO-*d*_6_) data, see Supplementary Table 6 and Supplementary Figures 12, 13. ESIMS *m/z* 309.2 [M−H]^–^.

Compound **18**: IR (KBr) ν_*max*_ 3122, 2355, 2337, 1714, 1653, 1504, 1392, 1236, 1109, 1030, 827, 671 cm^–1^. HR ESIMS *m/z* 325.0716 [M−H]^–^ (calcd. for C_18_H_13_O_6_, 325.0707) (Supplementary Figure 14). ^1^H NMR (600 MHz, DMSO-*d*_6_) and ^13^C NMR (150 MHz, DMSO-*d*_6_) data, see Table 2 and Supplementary Figures 15, 16.

Compound **19**: IR (KBr) ν_*max*_ 3134, 2357, 2339, 1682, 1653, 1556, 1508, 1456, 1279, 1184, 1107, 1032, 872, 667 cm^–1^. HRESI MS *m/z* 343.0808 [M+H]^+^ (calcd. for C_18_H_15_O_7_, 343.0812) (Supplementary Figure 17). ^1^H NMR (600 MHz, DMSO-*d*_6_) and ^13^C NMR (150 MHz, DMSO-*d*_6_) data, see Table 2 and Supplementary Figures 18, 19.

#### Synthesis of Compounds **20**–**22**

Compound **5** (1 g, 3.0 mmol) was dissolved in CH_2_Cl_2_ (100 mL), and BBr_3_ (27 mL, 18 mmol, 17% in CH_2_Cl_2_) was added at −20°C under the argon atmosphere. After stirring overnight at rt, H_2_O (40 mL) was added to quench the reaction at 0°C. The CH_2_Cl_2_ was evaporated and then EtOAc (200 mL) was added. The EtOAc phase was washed with H_2_O (20 mL × 4) and concentrated *in vacuo*. The residue was dissolved in MeOH (50 mL), and silica gel (200–300 mesh, 20 g) was added to the solution. The reaction mixture was stirred overnight at rt and then the solvent was evaporated *in vacuo*. The residue was purified by FCC eluting with EtOAc-CH_2_Cl_2_ (v/v 1:1) to provide compound **20** (550 mg, 1.78 mmol, 59% yield) as a dark red solid (*R*_*f*_ 0.2). By the same procedures, compound **21** (389 mg, 1.2 mmol, 86% yield, *R*_*f*_ 0.2) was prepared from the reaction of compound **6** (500 mg, 1.4 mmol) with BBr_3_ (12.7 mL, 8.4 mmol) in CH_2_Cl_2_ and then 200–300 mesh silica gel (12 g) in MeOH, and purified by FCC eluting with EtOAc-CH_2_Cl_2_ (v/v 2:1), while compound **22** (58 mg, 0.17 mmol, 63% yield, *R*_*f*_ 0.3) was prepared from the reaction of compound **7** (100 mg, 0.27 mmol) with BBr_3_ (2.4 mL, 1.62 mmol) in CH_2_Cl_2_ and then 200–300 mesh silica gel (6 g) in MeOH, and purified by FCC eluting with EtOAc-CH_2_Cl_2_ (v/v 4:1).

Compound **20**: ^1^H NMR (600 MHz, DMSO-*d*_6_) and ^13^C NMR (150 MHz, DMSO-*d*_6_) data, see Supplementary Table 6 and Supplementary Figures 20, 21. ESIMS *m/z* 307.1 [M−H]^–^.

Compound **21**: IR (KBr) *ν*_*max*_ 3122, 2359, 2339, 1653, 1602, 1510, 1404, 1335, 1281, 1228, 1176, 1101, 841, 667, 538 cm^–1^. HR ESIMS *m/z* 325.0704 [M+H]^+^ (calcd. for C_18_H_13_O_6_, 325.0707) (Supplementary Figure 22). ^1^H NMR (600 MHz, DMSO-*d*_6_) and ^13^C NMR (150 MHz, DMSO-*d*_6_) data, see Table 3 and Supplementary Figures 23, 24.

**TABLE 3:** ^1^H (600 MHz) and ^13^C (150 MHz) NMR data of compounds **21**, **22**, and **25** in DMSO-*d*_6_.

**Position**	**21**		**22**		**25^a^**	
	***δ*_*C*_**	***δ*_*H*_ (*J* in Hz)^b^**	***δ*_*C*_**	***δ*_*H*_ (*J* in Hz)**	***δ*_*C*_**	***δ*_*H*_ (*J* in Hz)**
1	122.4, C		122.4, C		130.8, C	
2	119.0, CH	6.81, d (2.0)	118.9, CH	6.81, d (2.0)	124.9, CH	7.10, s
3	144.2, C		144.2, C		141.5, C	
4	145.1, C		145.0, C		141.3, C	
5	114.7, CH	6.74, d (8.2)	114.7, CH	6.73, d (8.2)	123.2, CH	7.32, d (8.5)
6	121.6, CH	6.66, dd (8.2, 2.0)	121.6, CH	6.66, dd (8.2, 2.0)	128.4, CH	7.17, d (8.5)
1′	118.4, C		118.3, C		122.7, C	
2′	152.2, C		152.2, C		142.4, C	
3′	183.4, C		183.3, C		143.0, C	
4′	141.6, C		141.6, C		132.9, C	
5′	131.2, CH	6.72, s	131.2, CH	6.65, s	110.5, CH	7.04, s
6′	187.0, C		186.9, C		152.6, C	
1″	123.1, C		123.5, C		135.4, C	
2″	130.8, CH	7.46, d (8.5)	116.6, CH	7.04, d (2.2)	124.0, CH	7.54, s
3″	115.3, CH	6.85, d (8.5)	147.5, C		141.9, C	
4″	159.1, C		145.1, C		141.6, C	
5″	115.3, CH	6.85, d (8.5)	115.5, CH	6.80, d (8.0)	123.7, CH	7.39, d (8.5)
6″	130.8, CH	7.46, d (8.5)	120.9, CH	6.94, dd (8.0, 2.0)	127.1, CH	7.58, d (8.5)
3′-OMe					60.8, CH_3_	3.42, s
6′-OMe					56.2, CH_3_	3.79, s

*^*a*^The ^1^H and ^13^C NMR Data for five acetoxy were δ_*H*_ 2.06 (s, 3H), 2.29 (s, 3H), 2.31 (s, 9H), and δ_*C*_ 168.3 × 2 (C), 168.4 × 2 (C), 168.7 (C), 20.0 (CH_3_), 20.4 × 4 (CH_3_), respectively. ^*b*^d, dd, s respectively means doublet, a doublet of doublets, and singlet.*

Compound **22**: IR (KBr) ν_*max*_ 3417, 3130, 2357, 2333, 1655, 1618, 1552, 1506, 1281, 1201, 1099, 933, 876, 820, 779, 667 cm^–1^. HR ESIMS *m/z* 341.0649 [M + H]^+^ (calcd. for C_18_H_13_O_7_, 341.0656) (Supplementary Figure 25). ^1^H NMR (600 MHz, DMSO-*d*_6_) and ^13^C NMR (150 MHz, DMSO-*d*_6_) data, see Table 3 and Supplementary Figures 26, 27.

#### Synthesis of Compounds **23**–**25**

Compound **5** (30 mg, 89 μmol) was dissolved in CH_2_Cl_2_ (3 mL), then DMAP (102 mg, 0.8 mmol) and acetic anhydride (0.08 mL, 0.8 mmol) were added sequentially. After stirring for 2 h at 40°C, the CH_2_Cl_2_ was evaporated and EtOAc (20 mL) was added. The obtained organic phase was washed with H_2_O (20 mL × 4), dried over anhydrous Na_2_SO_4_, and concentrated *in vacuo*. The residue was purified by SepaBean machine eluting with 5–70% MeOH/H_2_O to provide **23** (36 mg, 78 μmol, 88% yield) as a white solid. By the same procedures, compounds **24** (35 mg, 67 μmol, 79% yield) and **25** (38 mg, 66 μmol, 81% yield) were, respectively, prepared from the reaction of compound **6** (30 mg, 85 μmol) with DMAP (124 mg, 1.02 mmol) and acetic anhydride (0.1 mL, 1.02 mmol) in CH_2_Cl_2_ (3 mL), and compound **7** (30 mg, 81 μmol) with DMAP (148 mg, 1.21 mmol) and acetic anhydride (0.12 mL, 1.21 mmol) in CH_2_Cl_2_ (3 mL). Both compounds **24** and **25** were purified by SepaBean machine eluting with 5–65% MeOH/H_2_O.

Compound **23**: IR (KBr) ν_*max*_ 3132, 2356, 2333, 1761, 1653, 1562, 1522, 1479, 1396, 1228, 1201, 1667, 1107, 1082, 1009, 920, 841, 671 cm^–1^. HR ESIMS *m/z* 465.1537 [M+H]^+^ (calcd. for C_26_H_25_O_8_, 465.1544) (Supplementary Figure 28). ^1^H NMR (600 MHz, DMSO-*d*_6_) and ^13^C NMR (150 MHz, DMSO-*d*_6_) data, see Supplementary Table 6 and Supplementary Figures 29, 30.

Compound **24**: ^1^H NMR (600 MHz, DMSO-*d*_6_) and ^13^C NMR (150 MHz, DMSO-*d*_6_) data, see Supplementary Table 6 and Supplementary Figures 31, 32. ESIMS *m/z* 545.0 [M+Na]^+^.

Compound **25**: IR (KBr) ν_*max*_ 3128, 2359, 2337, 1768, 1655, 1558, 1522, 1475, 1400, 1203, 1115, 1093, 899, 671 cm^–1^. HR ESIMS *m/z* 603.1470 [M+Na]^+^ (calcd. for C_30_H_28_O_12_Na, 603.1473) (Supplementary Figure 33). ^1^H NMR (600 MHz, DMSO-*d*_6_) and ^13^C NMR (150 MHz, DMSO-*d*_6_) data, see Table 3 and Supplementary Figures 34, 35.

### Oxygen Radical Absorbance Capacity (ORAC) Assay

The anti-oxidative activity of compounds was evaluated by ORAC assay (Huang et al., 2002) that was carried out mainly by using 2,2′-azobis(2-amidinopropane) dihydrochloride (AAPH, 153.0 μM), fluorescein (FL, 81.6 nM), testing compounds, and trolox as a positive control, all of which were dissolved in phosphate buffer solution (PBS, 75 mM, pH 7.4). The concentrations were 6.25 μM for compounds **4**–**7** and trolox, 12.5 μM for compounds **1**–**3**, **8**–**16** and trolox, and 25.0 μM for compounds **17**–**25** and trolox, respectively (Supplementary Figure 36). In short, each 25 μL of testing compounds, blank (PBS), negative (PBS) and trolox, and 150 μL of FL were added in each well and incubated at 37°C for 10 min. Each 25 μL of AAPH was then added to the testing compounds, blank and trolox groups, and 25 μL of PBS was added to the negative group. Fluorescence intensity of each well was measured one time every 1 min for 90 cycles using a Fluoroskan Ascent FL plate-reader (Thermo Scientific Varioskan LUX) at excitation of λ 485 nm and emission of λ 530 nm. The relative fluorescence intensity *f* was equaled to the ratio of the absolute fluorescence reading to the initial fluorescence reading, and the net area under curve (AUC) was obtained by subtracting the AUC of the blank from that of the compound. The AUC was calculated as 0.5 + *f*_1_ +… *f*_*i*_ +… + *f*_89_ + 0.5 × *f*_90_, in which *f*_*i*_ means the ratio of fluorescence reading at time *i* to the initial fluorescence reading. The final ORAC values were calculated as micromole of trolox equivalents per micromole of the compound (μmole TE/μmole) by using a regression equation between the trolox concentration and the net area under the FL decay curve. That is, the relative ORAC value = (AUC_*compound*_ – AUC_*blank*_)/(AUC_*trolox*_ – AUC_*blank*_).

### DPPH Radical-Scavenging Assay

The anti-oxidative activity of compounds was also evaluated by 2,2-diphenyl-1-picrylhydrazyl (DPPH) radical-scavenging assay (Wang et al., 2007). The experiment was divided into the following five groups, blank (methanol, MeOH), sample (mix compound and DPPH solution), background (pure compound solution), negative (pure DPPH solution), and positive [mix vitamin C (VC) and DPPH solution] controls. DPPH (0.15 mM), compounds (1–100 μM), and VC (1–100 μM) that was regarded as a compound sample in the following procedures all were dissolved in MeOH. Each 160 μL of MeOH was placed in negative control and blank groups, while each 160 μL of testing compounds or VC was placed in sample and background groups. Then, MeOH (each 40 μL) was, respectively, added to blank and background groups, while 40 μL of DPPH was, respectively, added to negative, positive, and sample controls. After 30-min incubation in the dark at rt, the decrease in DPPH radical concentration was monitored by measuring the absorbance at λ 517 nm with a microplate reader (Multiskan Spectrum, Thermo Scientific Varioskan LUX). The DPPH radical-scavenging rate was calculated as:

$$\text{Scavenging rate (\%)}=[\left(A_{n\,e\,g\,a\,t\,i\,v\,e}-A_{b\,l\,a\,n\,k}\right)-\left(A_{s\,a\,m\,p\,l\,e}-A_{b\,a\,c\,k\,g\,r\,o\,u\,n\,d}\right)]/\,\left(A_{n\,e\,g\,a\,t\,i\,v\,e}-A_{b\,l\,a\,n\,k}\right)\times 100\%.$$

The IC_50_ (half maximal inhibitory concentration) values of compounds and VC were calculated by SPSS (Statistical Package for the Social Sciences) software from the radical-scavenging rates at the final concentrations of 100, 50, 10, 5, and 1 μM.

### α-Glucosidase Inhibitory Assays

#### α-Glucosidase Inhibitions in *Saccharomyces cerevisiae*

The inhibitions of the compounds against α-glucosidase from *Saccharomyces cerevisiae* were assayed by reported method (Xu et al., 2018). The testing compounds were dissolved in dimethyl sulfoxide (DMSO) to obtain stock solution (10 mM) and then diluted into the concentrations by PBS (pH 6.8), while α-glucosidase (2.0 U/mL, Sigma), 4-nitrophenyl-α-D-glucopyranoside (PNPG, 2.5 mM, Macklin), Na_2_CO_3_ (0.2 M), and acarbose (2.5 mg/mL, Sigma) were directly dissolved in PBS. 20 μL of the compound solution and acarbose were, respectively, mixed in a 96-well microplate with 20 μL of α-glucosidase and 60 μL of PBS as the drug and positive groups, while the pure PBS solution was used as the blank group. After incubation for 15 min at 37°C, 20 μL of PNPG solution was added to each well of testing groups and further incubated at 37°C for 30 min. Finally, 80 μL of Na_2_CO_3_ solution was added to each well to stop the reaction and the absorbance was measured by a microplate reader (Multiskan Spectrum, Thermo Scientific Varioskan LUX) at λ 405 nm. The inhibitory rate (%) was calculated as [1 − (A_*drug*_/A_*blank*_)] × 100%. The IC_50_ values were calculated by SPSS software from the drug inhibitory rates at the final concentrations of 500, 250, 50, 25, 5, and 1 μM (Table 3).

#### α-Glucosidase Inhibitions in Caco-2 Cell Line

The α-glucosidase inhibition assay was also carried out in caco-2 cell line (Hansawasdi and Kawabata, 2006). Caco-2 cells at logarithmic growth stage were inoculated in a 6-well plate with an inoculation density of 4000/cm^2^ and cultured in an incubator with 5% CO_2_ at 37°C in a Dulbecco’s Modified Eagle Medium (DMEM) supplemented with 10% fetal bovine serum, 1% non-essential amino acid, 1% penicillin/streptomycin, 1% L-glutamine, and 0.25 mg plasmocin. The DMEM medium was changed one time every 2 days for 24 days. Then, the culture medium was removed, and the cell surface was washed three times by a PBS solution (pH 7.4) at 37°C. 1.0 mL of sucrose/maltose (both 28 mM) PBS solution was added to the control well, 1 mL of PBS was added to the blank well, 0.2 mL of compounds or acarbose (positive control) with different concentration and 0.8 mL of above sucrose/maltose solution were added to the drug well. The final concentration gradients of compounds and acarbose were 1.0, 0.3, 0.1, 0.03, 0.01 μM and 10000, 3000, 1000, 300, 100 μg/mL, respectively. The obtained solutions for the enzymatic hydrolysis reactions of sucrose and maltose were incubated at 37°C for 60 min. After terminating the reactions in an ice bath for 10 min, 10 μL of the reaction mixture was added into 1 mL of the glucose kit (Nanjing Jiancheng Bioengineering Institute Co., Ltd.) and maintain 10 min at 37°C. The α-glucosidase inhibitory activity of the compounds was then determined by measuring the glucose content in the reaction solution (pipette 100 μL reaction solution into 96-well plate) via the absorbance at λ 505 nm with a microplate reader (BioTek Synergy H1, BioTek, VT, United States). The α-glucosidase inhibitory rate (%) was calculated as [1 − (A_*drug*_– A_*blank*_)/ (A_*control*_– A_*blank*_)] × 100%. The IC_50_ values were calculated as showed in Table 3 by SPSS software.

The cytotoxic effects on the coca-2 cells were evaluated by the CTG assay (Elisia and Kitts, 2008; Wang et al., 2019). Briefly, coca-2 cells were seeded in 96-well plates at a density of 2 × 10^3^ cells/well and treated with the final concentration of 1.0 μM of the compounds. After 72 h incubation, 100 μL of CTG solution (Promega) was added into each well. The luminescence value was tested by using a microplate reader (BioTek Synergy H1) after staying at rt for 10 min.

## Results and Discussion

### Metabolic Regulation of the Fungus

After adding the leaves of *Eucommia ulmoides* in the rice medium, both the production and the α-glucosidase inhibitory activity of the EtOAc extracts of the solid-state fermentation increased significantly from 5.3 to 9.5 g/kg and from the IC_50_ value of 15.0 to 2.0 μg/mL, respectively. The original *p*-terphenyl products **5**–**7**, **10**, and **12** in the rice medium also largely increased by adding the leaves of *E*. *ulmoides*. In addition, the number of the *p*-terphenyl-type chromophores also increased significantly. For example, *p*-terphenyls **1**–**4**, **8**, **9**, **11**, and **13**–**16** were newly produced after adding the leaves of *E*. *ulmoides* (Figure 1). The results indicated that the content and diversity of the microbial natural products could increase highly by adding the host materials to the culture media of the microorganisms via the chemical microbe-host interaction.

### The Identification of the New *p*-Terphenyl (1)

**FIGURE 3:**
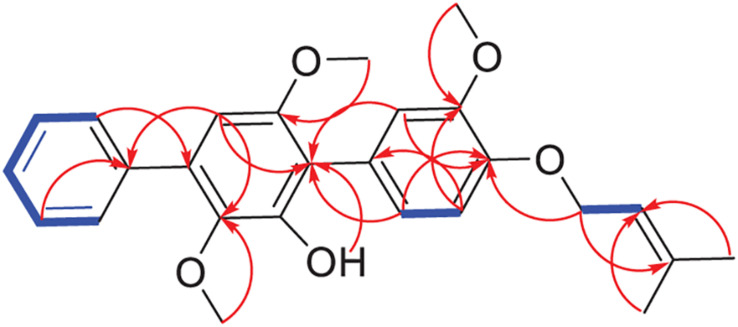
Key HMBC (red arrows) and COSY (blue bold lines) of compound **1**.

Compound **1** was obtained as a white powder. Its molecular formula was determined as C_26_H_28_O_5_ according to its HR-ESIMS peak at *m/z* 443.1837 [M+Na]^+^ (Supplementary Figure 2). The NMR spectra displayed nine sp^2^- non-hydrogenated carbons, ten sp^2^-methines, one sp^3^-methylene, three methoxyls, and two methyls (Supplementary Figures 3–5). Except for the lack of 4″-hydroxyl, these data (Table 2) were very similar to those reported 3-methoxyterprenin (**13**), indicating compound **1** as a highly oxygenated *p*-terphenyl. The signals of five mono-substituted phenyl protons at *δ*_*H*_ 7.61 (d, *J* = 7.5 Hz, H-2″/H-6″), 7.47 (t, *J* = 7.5 Hz, H-3″/H-5″), and 7.37 (t, *J* = 7.5 Hz, H-4″) which showed ^1^H-^1^H COSY correlations (Figure 3 and Supplementary Figure 6) of H-2″/H-3″/H-4″ confirmed replacement of 4″-OH in **13** by a hydrogen atom in **1**. Furthermore, the ^1^H-^1^H COSY between H-5 (*δ*_*H*_ 6.96) and H-6 (*δ*_*H*_ 6.82), H-1″′ (*δ*_*H*_ 4.53) and H-2″′ (*δ*_*H*_ 5.47), along with the key HMBC connections (Figure 3 and Supplementary Figure 7) from H-1″′ to C-3″′ (*δ*_*C*_ 136.9) and C-4 (*δ*_*C*_ 146.7), H-4″′ (*δ*_*H*_ 1.72) to C-2″′ (*δ*_*C*_ 128.7) and C-5″′ (*δ*_*C*_ 25.5), and H-2″/H-6″ to C-4′ (*δ*_*C*_ 132.7) supported the structure. Thus, compound **1** was identified as 3-*O*-methyl-4″-deoxyterprenin.

### Synthesis of *p*-Terphenyls **17**–**25**

**SCHEME 2:**
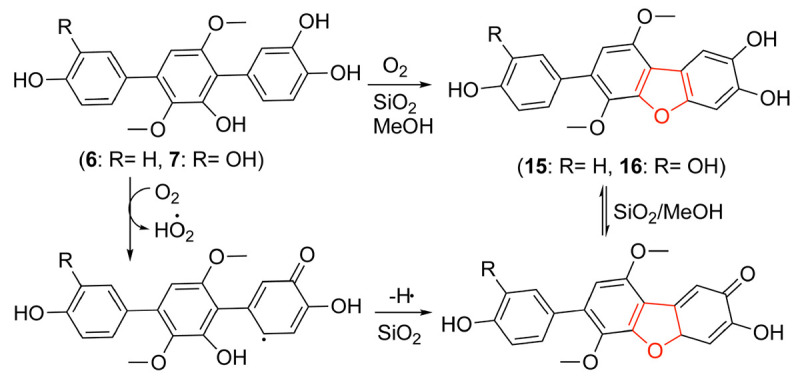
A possible mechanism forming **15** and **16** by an oxidative cyclization of **6** and **7**.

As shown in Scheme 1, compounds **17***–***19** were synthesized from compounds **5***–***7** by demethylation reaction using BBr_3_, which were further transformed to compounds **20***–***22** by oxidation of air in the system of silica gel and MeOH. The acetylation of compounds **5***–***7** provided compounds **23***–***25** by Ac_2_O/DMAP. It is interesting that compounds **15** and **16** could be synthesized from compounds **6** and **7** by an oxidative dehydrocyclization of air in the system of silica gel and MeOH. But compound **5** cannot undergo the same reaction to form the corresponding 2,2′-oxygen bridged *p*-terphenyl derivative, indicating that the oxidative cyclization might be carried out by a radical process. That is, compounds **6** and **7** formed a radical intermediate **a** which underwent an intramolecular cyclization to generate the keto intermediate **b** in the presence of SiO_2_ and O_2_. Compounds **15** and **16** were then yielded by a keto-enol tautomerization of the intermediate **b** in the SiO_2_ and MeOH (Scheme 2). To confirm the effect of silica gel and O_2_, the reaction of compound **6** was carried out in the four conditions, i.e., O_2_, silica gel/argon, silica gel/air, and silica gel/O_2_. The results showed that compound **6** could not be converted to compound **15** without silica gel. Both the reaction and conversion rates increased in the order of O_2_, silica gel/argon, silica gel/air, and silica gel/O_2_ (Supplementary Figure 37). And the silica gel acted as a catalyst to accelerate the tautomerization between keto and enol. The fact that a little compound **15** was also formed in the silica gel/argon system could be explained from the air adsorbed in the silica gel.

### The Bioactivities of *p*-Terphenyls

All of the obtained *p*-terphenyls (**1***–***25**) were tested for the anti-oxidative activity against DPPH radicals and ORAC as well as the α-glucosidase inhibitions. The results (Table 3) showed that compounds **4***–***7**, **17**, and **20** have a significant antioxidant capacity with the ORAC values of 6.8, 6.0, 5.7, 4.5, 4.8, and 6.1 μmole TE/μmole, respectively, indicating that 4-, 2′-, and 4″-hydroxys are active sites of *p*-terphenyls. When these hydroxyls were changed to hydrogens or etherified in part or wholly, the antioxidant capacity was greatly reduced. Compounds **4**, **6**, **7**, **9**, **11**, **12**, **19**, **21**, and **22** showed more potent DPPH radical-scavenging activity than VC with the IC_50_ value of 1.7, 1.4, 1.1, 1.6, 1.6, 1.8, 1.6, 1.7, and 2.8 (VC) μM, respectively, indicating that 4- and 2′-hydroxys are very important active sites of the *p*-terphenyls. As the disappearance of the two hydroxys, changing to hydrogens, methoxyls or acetoxyls, for example, the DPPH radical-scavenging activity of *p*-terphenyls was greatly reduced. However, the activity is still maintained when the 2′-hydroxy formed a furan ring with C-6.

The most obvious inhibition against α-glucosidase from *Saccharomyces cerevisiae* was observed for compounds **4**, **5**, **16**, **20**, and **21** whose IC_50_ values were 5.9, 2.8, 7.9, 8.9, and 4.0 μM, respectively. The results indicated that 4-, 2′,- and 4″-hydroxys are the most important active-sites for α-glucosidase inhibition of *p*-terphenyls, two or three of which were replaced by hydrogens, methoxyls or acetoxyls resulted in the loss or decrease of α-glucosidase inhibitory activity. Seven compounds (**5***–***7**, **15**, **16**, **20**, and **21**) with significant antioxidant and α-glucosidase inhibitory activities could be prepared on a large scale, were further tested for the α-glucosidase inhibitions in caco-2 cell line. As expected, these seven compounds exhibited the announced activity with the IC_50_ values of 0.38, 0.29, 0.36, 0.11, 0.36, 0.11, and 0.12 μM, respectively. It is interesting that these seven compounds were not toxic to the caco-2 cells at the concentration of 1 μM, whose inhibitory rate was 0.4%, 0.2%, 0.5%, 20.5%, 2.7%, 0.4%, and 1.2%, respectively.

## Conclusion

There is a mutually beneficial relationship between endophytes and their host plants. Adding the host plants to the culture medium of endophytes could enhanced the metabolic potential of the endophytic strains and thus enriched the chemodiversity of the microbial natural products. *p*-Terphenyls, especially those 4,2′,4″-trihydroxy or 4,4″-dihydroxy-1,2,1′,2′-furan substituted ones, have a stronger antioxidant activity, α-glucosidase inhibitory activity and lower cytotoxicity, implying their potential use in the fight against diabetes as the drug leads or dietary supplements.

## Data Availability Statement

Publicly available datasets were analyzed in this study. This data can be found here: GenBank No. KY038594.

## Author Contributions

YX isolated the fungus and compounds, performed the structure elucidation, and assayed part of the bioactivity. YW synthesized the compounds. DW assayed part of the bioactivity. WH did fermentation and extraction. LW directed the implementation of the study and prepared the manuscript. WZ designed the study and revised the manuscript. All authors contributed to the article and approved the submitted version.

## Conflict of Interest

The authors declare that the research was conducted in the absence of any commercial or financial relationships that could be construed as a potential conflict of interest.
