# Supplementary material for: p-Terphenyls From Aspergillus sp. GZWMJZ-055: Identification, Derivation, Antioxidant and α-Glycosidase Inhibitory Activities
Source: Front Microbiol. 2021 Feb 25;12:654963. doi: 10.3389/fmicb.2021.654963 (PMC7947296; doi:10.3389/fmicb.2021.654963)

frontiers

Supplementary Material

p-Terphenyls from *Aspergillus* sp. GZWMJZ-055: Identification, Derivation, Antioxidant and α-Glycosidase Inhibitory Activities

Yanchao Xu[1,2,3†], Yong Wang[1,2,3†], Dan Wu[1,3], Wenwen He[1,3], Liping Wang[1,2,3*], and Weiming Zhu[1,4*]

[1] State Key Laboratory of Functions and Applications of Medicinal Plants, Guizhou Medical University, Guiyang 550014, China

[2] School of Pharmaceutical Sciences, Guizhou Medical University, Guiyang 550025, China

[3] Key Laboratory of Chemistry for Natural Products of Guizhou Province, Chinese Academy of Sciences, Guiyang 550014, China

[4] Laboratory for Marine Drugs and Bioproducts, Pilot National Laboratory for Marine Science and Technology, School of Medicine and Pharmacy, Ocean University of China, Qingdao 266003, China.

***Correspondence:**
Weiming Zhu
weimingzhu@ouc.edu.cn

Liping Wang
lipingw2006@163.com

†These authors contributed equally to this work.

List of Supporting Information

Table S1. ^{1}H (600 MHz) and ^{13}C (150 MHz) NMR Data for Compounds **2**–**4** in DMSO-*d*$_6$ S3
Table S2. ^{1}H (600 MHz) and ^{13}C (150 MHz) NMR Data of compounds **5**–**7** in DMSO-*d*$_6$ S3
Table S3. ^{1}H (600 MHz) and ^{13}C (150 MHz) NMR Data of compounds **8**–**10** in DMSO-*d*$_6$ S4
Table S4. ^{1}H (600 MHz) and ^{13}C (150 MHz) NMR Data of compounds **11**–**13** in DMSO-*d*$_6$ S4
Table S5. ^{1}H (600 MHz) and ^{13}C (150 MHz) NMR Data of compounds **14**–**16** in DMSO-*d*$_6$ S5
Table S6. ^{1}H (600 MHz) and ^{13}C (150 MHz) NMR Data of compounds **17**, **20**, **23** and **24** in DMSO-*d*$_6$ S5
Figure S1. Phylogenetic tree of ITS sequence for *Aspergillus* sp. GZWMJZ-055 .... S6
Figure S2. HRESIMS spectrum of compound **1** S6
Figure S3. ^{1}H NMR spectrum of compound **1** in DMSO-*d*$_6$ S7
Figure S4. ^{13}C NMR spectrum of compound **1** in DMSO-*d*$_6$ S7
Figure S5. HMQC spectrum of compound **1** in DMSO-*d*$_6$ S8
Figure S6. ^{1}H-^{1}H COSY spectrum of compound **1** in DMSO-*d*$_6$ S8
Figure S7. HMBC spectrum of compound **1** in DMSO-*d*$_6$ S9
Figure S8. ^{1}H NMR spectrum of compound **15** in DMSO-*d*$_6$ S9
Figure S9. ^{13}C NMR spectrum of compound **15** in DMSO-*d*$_6$ S10
Figure S10. ^{1}H NMR spectrum of compound **16** in DMSO-*d*$_6$ S10

Figure S11. ^{13}C NMR spectrum of compound **16** in DMSO-d_6 .... S11
Figure S12. ^{1}H NMR spectrum of compound **17** in DMSO-d_6 .... S11
Figure S13. ^{13}C NMR spectrum of compound **17** in DMSO-d_6 .... S12
Figure S14. HRESIMS spectrum of compound **18** S12
Figure S15. ^{1}H NMR spectrum of compound **18** in DMSO-d_6 .... S13
Figure S16. ^{13}C NMR spectrum of compound **18** in DMSO-d_6 .... S13
Figure S17. HRESIMS spectrum of compound **19** S14
Figure S18. ^{1}H NMR spectrum of compound **19** in DMSO-d_6 .... S14
Figure S19. ^{13}C NMR spectrum of compound **19** in DMSO-d_6 .... S15
Figure S20. ^{1}H NMR spectrum of compound **20** in DMSO-d_6 .... S15
Figure S21. ^{13}C NMR spectrum of compound **20** in DMSO-d_6 .... S16
Figure S22. HRESIMS spectrum of compound **21** S16
Figure S23. ^{1}H NMR spectrum of compound **21** in DMSO-d_6 .... S17
Figure S24. ^{13}C NMR spectrum of compound **21** in DMSO-d_6 .... S17
Figure S25. HRESIMS spectrum of compound **22** S18
Figure S26. ^{1}H NMR spectrum of compound **22** in DMSO-d_6 .... S18
Figure S27. ^{13}C NMR spectrum of compound **22** in DMSO-d_6 .... S19
Figure S28. HRESIMS spectrum of compound **23** S19
Figure S29. ^{1}H NMR spectrum of compound **23** in DMSO-d_6 .... S20
Figure S30. ^{13}C NMR spectrum of compound **23** in DMSO-d_6 .... S20
Figure S31. ^{1}H NMR spectrum of compound **24** in $CDCl_3$ S21
Figure S32. ^{13}C NMR spectrum of compound **24** in $CDCl_3$ S21
Figure S33. HRESIMS spectrum of compound **25** S22
Figure S34. ^{1}H NMR spectrum of compound **25** in $CDCl_3$ S22
Figure S35. ^{13}C NMR spectrum of compound **25** in $CDCl_3$ S23
Figure S36. ORAC curves of compounds **1**–**25** and trolox S23
Figure S37. HPLC detection for transformation of compound **6** to compound **15** in different conditions and reaction time S24

Table S1. ^{1}H (600 MHz) and ^{13}C (150 MHz) NMR Data of compounds **2**–**4** in DMSO-d_6

position	**2**		**3**		**4**	
	δ_C	δ_H (J in Hz)	δ_C	δ_H (J in Hz)	δ_C	δ_H (J in Hz)
1	134.4, C		123.9, C		124.8, C	
2	130.9, CH	7.29, d (7.9)	131.4, CH	7.11, d (8.5)	131.9, CH	7.09, d (8.5)
3	127.5, CH	7.36, t (7.9)	114.6, CH	6.79, d (8.5)	114.5, CH	6.76, d (8.5)
4	126.4, CH	7.26, t (7.9)	156.3, C		155.9, C	
5	127.5, CH	7.36, t (7.9)	114.6, CH	6.79, d (8.5)	114.5, CH	6.76, d (8.5)
6	130.9, CH	7.29, d (7.9)	131.4, CH	7.11, d (8.5)	131.9, CH	7.09, d (8.5)
1'	116.8, C		124.5, C		117.2, C	
2'	148.1, C		151.5, C		145.0, C	
3'	139.3, C		144.3, C		136.2, C	
4'	133.0, C		133.9, C		129.6, C	
5'	102.9, CH	6.42, s	108.0, CH	6.73, s	103.3, CH	6.36, s
6'	152.9, C		152.9, C		150.5, C	
1"	128.6, C		138.0, C		127.9, C	
2"	129.8, CH	7.45, d (8.5)	129.0, CH	7.57, d (7.3)	130.2, CH	7.42, d (8.5)
3"	115.2, CH	6.85, d (8.5)	128.2, CH	7.46, t (7.3)	114.9, CH	6.81, d (8.5)
4"	156.8, C		127.3, CH	7.37, t (7.3)	156.3, C	
5"	115.2, CH	6.85, d (8.5)	128.2, CH	7.46, t (7.3)	114.9, CH	6.81, d (8.5)
6"	129.8, CH	7.45, d (8.5)	129.0, CH	7.57, d (7.3)	130.2, CH	7.42, d (8.5)
3-OMe						
2'-OMe	60.5, CH_3	3.52, s				
3'-OMe	60.3, CH_3	3.51, s				
6'-OMe	55.8, CH_3	3.67, s			55.7, CH_3	3.59, s

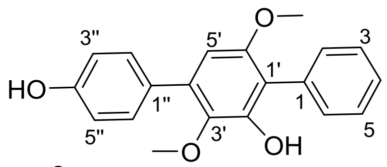

2

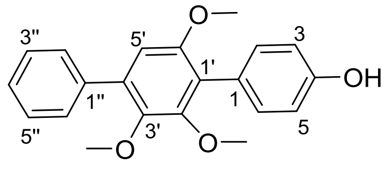

3

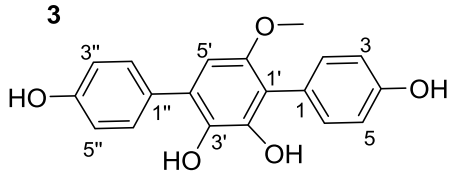

4

Table S2. ^{1}H (600 MHz) and ^{13}C (150 MHz) NMR Data of compounds **5**–**7** in DMSO-d_6

position	**5**		**6**		**7**	
	δ_C	δ_H (J in Hz)	δ_C	δ_H (J in Hz)	δ_C	δ_H (J in Hz)
1	124.4, C		125.0, C		125.1, C	
2	131.9, CH	7.09, d (8.5)	118.5, CH	6.68, d (1.9)	118.5, CH	6.68, d (2.0)
3	114.4, CH	6.76, d (8.5)	143.9, C		144.0, C	
4	156.1, C		144.3, C		144.3, C	
5	114.4, CH	6.76, d (8.5)	114.8, CH	6.71, d (8.0)	114.9, CH	6.71, d (8.0)
6	131.9, CH	7.09, d (8.5)	122.0, CH	6.54, dd (8.0, 1.9)	122.0, CH	6.54, dd (8.0, 2.0)
1'	116.9, C		117.2, C		117.1, C	
2'	148.2, C		148.2, C		148.2, C	
3'	139.3, C		139.3, C		139.3, C	
4'	132.4, C		132.3, C		132.5, C	
5'	102.9, CH	6.38, s	102.9, CH	6.37, s	102.9, CH	6.34, s
6'	153.1, C		153.1, C		153.1, C	
1"	128.5, C		128.8, C		129.4, C	
2"	129.7, CH	7.42, d (8.5)	129.8, CH	7.43, d (8.5)	116.2, CH	7.04, d (2.0)
3"	115.3, CH	6.85, d (8.5)	115.2, CH	6.83, d (8.5)	145.0, C	
4"	157.1, C		156.8, C		144.8, C	
5"	115.3, CH	6.85, d (8.5)	115.2, CH	6.83, d (8.5)	115.6, CH	6.80, d (8.0)
6"	129.7, CH	7.42, d (8.5)	129.8, CH	7.43, d (8.5)	119.7, CH	6.88, dd (8.0, 2.0)
3'-OMe	60.1, CH_3	3.29, s	60.1, CH_3	3.29, s	60.1, CH_3	3.31, s
6'-OMe	55.6, CH_3	3.63, s	55.6, CH_3	3.62, s	55.6, CH_3	3.62, s

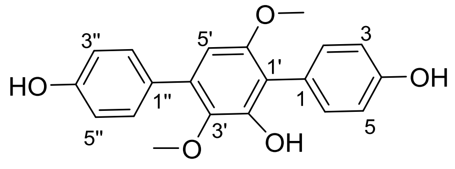

5

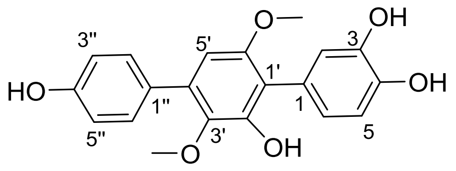

6

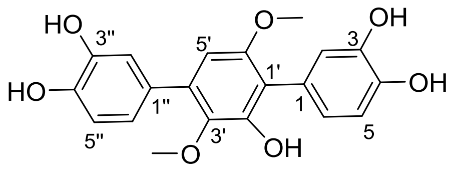

7

Table S3. ^{1}H (600 MHz) and ^{13}C (150 MHz) NMR Data of compounds **8**–**10** in DMSO-d_6

position	**8**		**9**		**10**	
	δ_C	δ_H (J in Hz)	δ_C	δ_H (J in Hz)	δ_C	δ_H (J in Hz)
1	124.4, C		124.8, C		123.8, C	
2	131.9, CH	7.10, d (8.5)	118.4, CH	6.68, s	131.5, CH	7.09, d (8.5)
3	114.4, CH	6.75, d (8.5)	144.0, C		114.7, CH	6.78, d (8.5)
4	156.0, C		144.3, C		156.3, C	
5	114.4, CH	6.75, d (8.5)	114.8, CH	6.71, d (8.0)	114.7, CH	6.78, d (8.5)
6	131.9, CH	7.10, d (8.5)	121.9, CH	6.54, d (8.0)	131.5, CH	7.09, d (8.5)
1'	117.8, C		118.1, C		124.2, C	
2'	148.3, C		148.2, C		151.6, C	
3'	139.5, C		139.4, C		144.3, C	
4'	132.5, C		132.3, C		134.0, C	
5'	103.2, CH	6.43, s	103.2, CH	6.42, s	107.8, CH	6.67, s
6'	153.2, C		153.2, C		152.9, C	
1''	138.3, C		138.2, C		128.6, C	
2''	128.7, CH	7.60, d (7.5)	128.7, CH	7.60, d (7.5)	130.2, CH	7.39, d (8.5)
3''	128.4, CH	7.45, t (7.5)	128.3, CH	7.45, t (7.5)	115.2, CH	6.84, d (8.5)
4''	127.3, CH	7.36, t (7.5)	127.2, CH	7.36, t (7.5)	156.9, C	
5''	128.4, CH	7.45, t (7.5)	128.3, CH	7.45, t (7.5)	115.2, CH	6.84, d (8.5)
6''	128.7, CH	7.60, d (7.5)	128.7, CH	7.60, d (7.5)	130.2, CH	7.39, d (8.5)
2'-OMe					60.4, CH_3	3.50, s
3'-OMe	60.4, CH_3	3.28, s	60.3, CH_3	3.29, s	60.4, CH_3	3.48, s
6'-OMe	55.7, CH_3	3.64, s	55.6, CH_3	3.64, s	55.9, CH_3	3.65, s

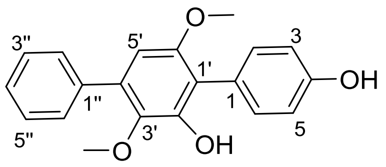

8

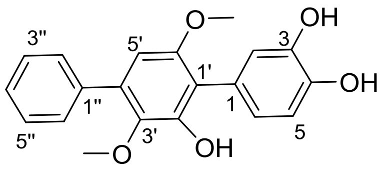

9

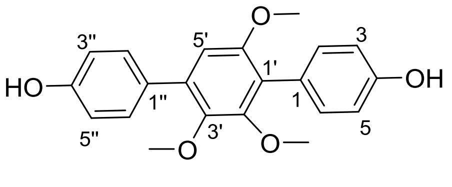

10

Table S4. ^{1}H (600 MHz) and ^{13}C (150 MHz) NMR Data of compounds **11**–**13** in DMSO-d_6

position	**11**		**12**		**13**	
	δ_C	δ_H (J in Hz)	δ_C	δ_H (J in Hz)	δ_C	δ_H (J in Hz)
1	124.4, C		125.0, C		126.7, C	
2	131.1, CH	6.96, d (2.0)	118.4, CH	6.67, d (2.0)	115.0, CH	6.86, d (1.9)
3	126.2, C		143.9, C		148.2, C	
4	153.6, C		144.3, C		146.6, C	
5	114.0, CH	6.77, d (8.0)	114.8, CH	6.70, d (8.0)	112.5, CH	6.95, d (8.2)
6	129.1, CH	6.93, dd (8.0, 2.0)	121.9, CH	6.52, dd (8.0, 2.0)	123.2, CH	6.80, dd (8.2, 1.9)
1'	118.0, C		117.2, C		116.8, C	
2'	148.2, C		148.2, C		148.2, C	
3'	139.5, C		139.2, C		139.3, C	
4'	132.3, C		132.4, C		132.7, C	
5'	103.2, CH	6.43, s	102.9, CH	6.33, s	103.0, CH	6.40, s
6'	153.2, C		153.1, C		153.1, C	
1''	138.3, C		128.8, C		128.7, C	
2''	128.7, CH	7.60, d (7.5)	129.7, CH	7.30, d (2.0)	129.8, CH	7.43, d (8.6)
3''	128.4, CH	7.46, t (7.5)	127.2, C		115.2, CH	6.85, d (8.6)
4''	127.2, CH	7.36, t (7.5)	154.3, C		156.8, C	
5''	128.4, CH	7.46, t (7.5)	114.8, CH	6.84, d (8.2)	115.2, CH	6.85, d (8.6)
6''	128.7, CH	7.60, d (7.5)	126.9, CH	7.23, dd (8.2, 2.0)	129.8, CH	7.43, d (8.6)
1'''	28.2, CH_2	3.22, d (6.6)	28.1, CH_2	3.25, d (7.3)	64.9, CH_2	4.52, d (6.7)
2'''	123.1, CH	5.31, t (6.6)	122.9, CH	5.32, t (7.3)	120.4, CH	5.47, t (6.7)
3'''	131.7, C		131.4, C		136.9, C	
4'''	17.7, CH_3	1.67, s	17.7, CH_3	1.68, s	18.1, CH_3	1.71, s
5'''	25.7, CH_3	1.68, s	25.6, CH_3	1.69, s	25.5, CH_3	1.76, s
3-OMe					55.5, CH_3	3.72, s
3'-OMe	60.4, CH_3	3.28, s	60.0, CH_3	3.28, s	60.1, CH_3	3.30, s
6'-OMe	55.6, CH_3	3.64, s	55.6, CH_3	3.61, s	55.6, CH_3	3.65, s

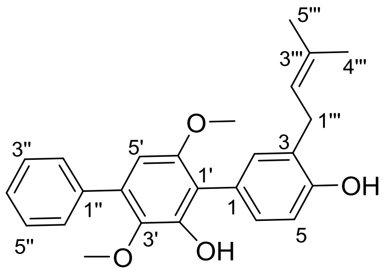

11

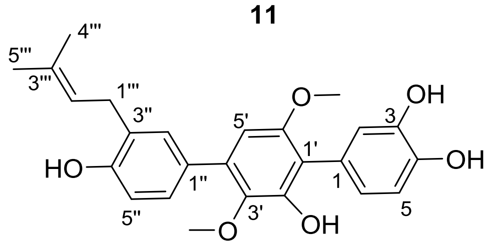

12

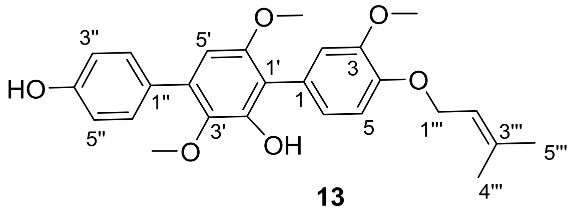

13

Table S5. ^{1}H (600 MHz) and ^{13}C (150 MHz) NMR Data of compounds **14**–**16** in DMSO-d_6

position	**14**		**15**		**16**	
	δ_C	δ_H (J in Hz)	δ_C	δ_H (J in Hz)	δ_C	δ_H (J in Hz)
1	114.8, C		114.0, C		113.9, C	
2	149.4, C		149.3, C		149.4, C	
3	98.5, CH	7.08, s	98.5, CH	7.07, s	98.5, CH	7.07, s
4	146.1, C		145.8, C		145.9, C	
5	142.7, C		142.5, C		142.6, C	
6	107.1, CH	7.38, s	107.0, CH	7.37, s	107.1, CH	7.37, s
1'	113.5, C		113.6, C		113.7, C	
2'	148.2, C		148.4, C		148.5, C	
3'	136.1, C		135.9, C		136.0, C	
4'	130.4, C		130.5, C		130.9, C	
5'	105.8, CH	6.75, s	105.5, CH	6.71, s	105.6, CH	6.68, s
6'	149.5, C		149.4, C		149.4, C	
1"	138.1, C		128.6, C		129.2, C	
2"	129.3, CH	7.58, d (7.5)	130.4, CH	7.42, d (8.2)	116.8, CH	7.03, s
3"	128.3, CH	7.45, t (7.5)	115.1, CH	6.85, d (8.2)	144.9, C	
4"	127.1, CH	7.36, t (7.5)	156.7, C		144.8, C	
5"	128.3, CH	7.45, t (7.5)	115.1, CH	6.85, d (8.2)	115.5, CH	6.81, d (7.8)
6"	129.3, CH	7.58, d (7.5)	130.4, CH	7.42, d (8.2)	120.4, CH	6.87, d (7.8)
3'-OMe	60.8, CH_3	3.78, s	60.6, CH_3	3.75, s	60.7, CH_3	3.74, s
6'-OMe	55.9, CH_3	3.98, s	55.8, CH_3	3.97, s	55.9, CH_3	3.97, s

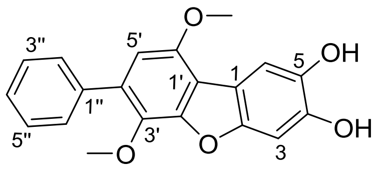

14

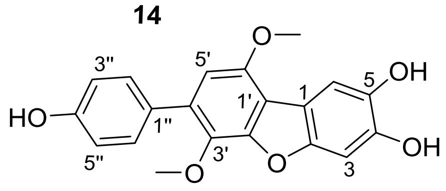

15

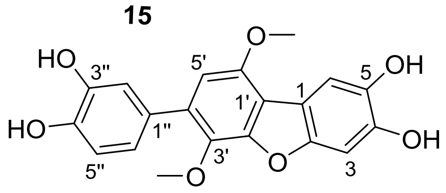

16

Table S6. ^{1}H (600 MHz) and ^{13}C (150 MHz) NMR Data of compounds **17**, **20**, **23** and **24** in DMSO-d_6

position	**17**		**20**		**23** [a]		**24** [b]	
	δ_C	δ_H (J in Hz)	δ_C	δ_H (J in Hz)	δ_C	δ_H (J in Hz)	δ_C	δ_H (J in Hz)
1	125.3, C		121.3, C		130.0, C		130.9, C	
2	131.9, CH	7.15, d (8.5)	132.1, CH	7.20, d (8.0)	131.0, CH	7.26, d (8.5)	124.9, CH	7.11, s
3	114.3, CH	6.75, d (8.5)	114.5, CH	6.79, d (8.0)	121.4, CH	7.18, d (8.5)	141.5, C	
4	155.7, C		157.0, CH		149.8, C		141.3, C	
5	114.3, CH	6.75, d (8.5)	114.5, CH	6.79, d (8.0)	121.4, CH	7.18, d (8.5)	123.2, CH	7.31, d (8.5)
6	131.9, CH	7.15, d (8.5)	132.1, CH	7.20, d (8.0)	131.0, CH	7.26, d (8.5)	128.5, CH	7.18, d (8.5)
1'	115.7, C		118.9, C		123.4, C		122.4, C	
2'	145.1, C		152.3, C		142.4, C		142.4, C	
3'	134.4, C		183.4, C		143.0, C		143.0, C	
4'	129.6, C		141.7, C		133.6, C		133.9, C	
5'	106.6, CH	6.28, s	131.2, CH	6.73, s	110.5, CH	7.00, s	110.6, CH	7.02, s
6'	148.2, C		187.0, C		152.7, C		152.5, C	
1"	128.4, C		123.1, C		134.7, C		134.6, C	
2"	129.9, CH	7.35, d (8.5)	130.8, CH	7.46, d (8.0)	129.9, CH	7.67, d (8.5)	130.0, CH	7.67, d (8.5)
3"	114.9, CH	6.80, d (8.5)	115.4, CH	6.85, d (8.0)	121.9, CH	7.25, d (8.5)	121.9, CH	7.25, d (8.5)
4"	156.2, C		159.2, C		150.1, C		150.1, C	
5"	114.9, CH	6.80, d (8.5)	115.4, CH	6.85, d (8.0)	121.9, CH	7.25, d (8.5)	121.9, CH	7.25, d (8.5)
6"	129.9, CH	7.35, d (8.5)	130.8, CH	7.46, d (8.0)	129.9, CH	7.67, d (8.5)	130.0, CH	7.67, d (8.5)
3'-OMe					60.7, CH_3	3.39, s	60.7, CH_3	3.39, s
6'-OMe					56.1, CH_3	3.76, s	56.1, CH_3	3.79, s

[a] The ^{1}H and ^{13}C NMR Data for three acetoxy were δ_H 2.02 (s, 3H), 2.29 (s, 3H), 2.30 (s, 3H), and δ_C 168.7 (C), 169.3 ×2 (C), 20.1 (CH_3), 20.9×2 (CH_3), respectively.

[b] The ^{1}H and ^{13}C NMR Data for four acetoxy were δ_H 2.05 (s, 3H), 2.30 (s, 3H), 2.31 (s, 6H), and δ_C 168.4×2 (C), 168.7 (C), 169.3 (C), 20.0 (CH_3), 20.4×2 (CH_3), 20.9 (CH_3), respectively.

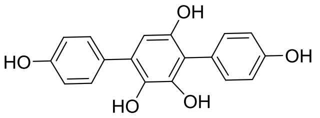

17

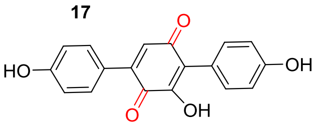

20

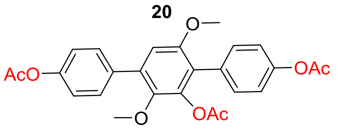

23

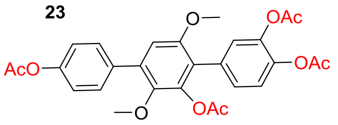

24

Figure S1. Phylogenetic tree of ITS sequence for *Aspergillus* sp. GZWMJZ-055

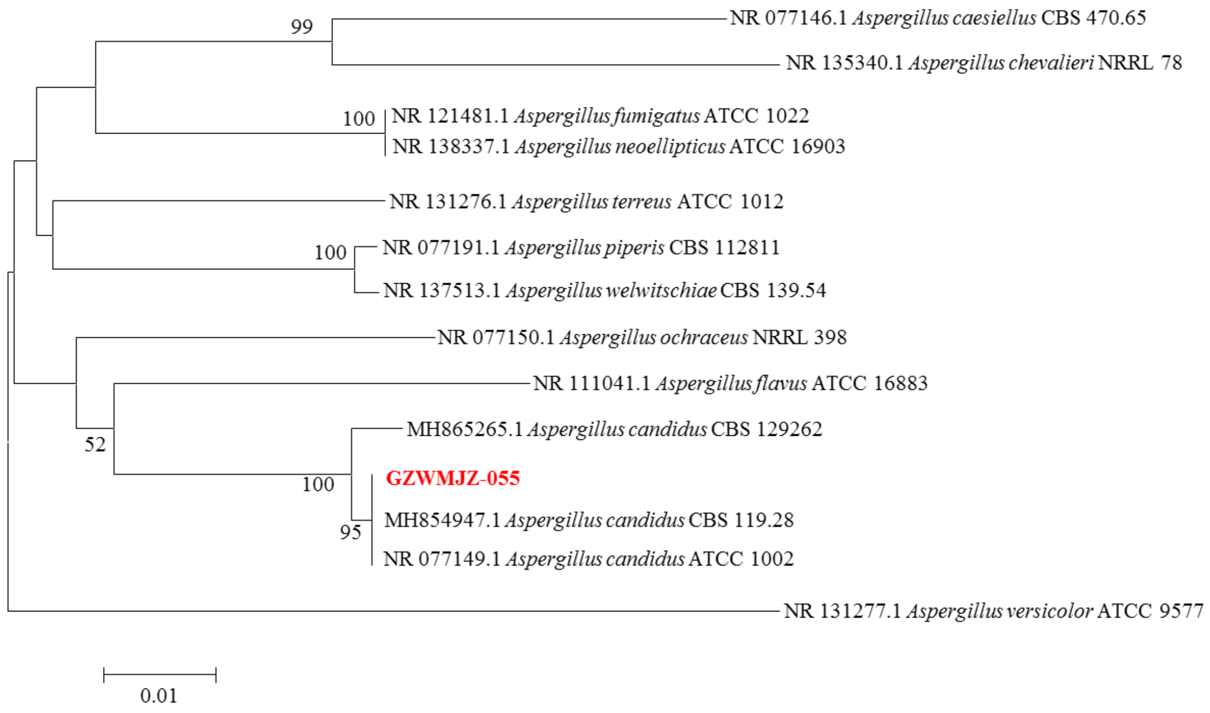

Figure S2. HRESIMS spectrum of compound **1**

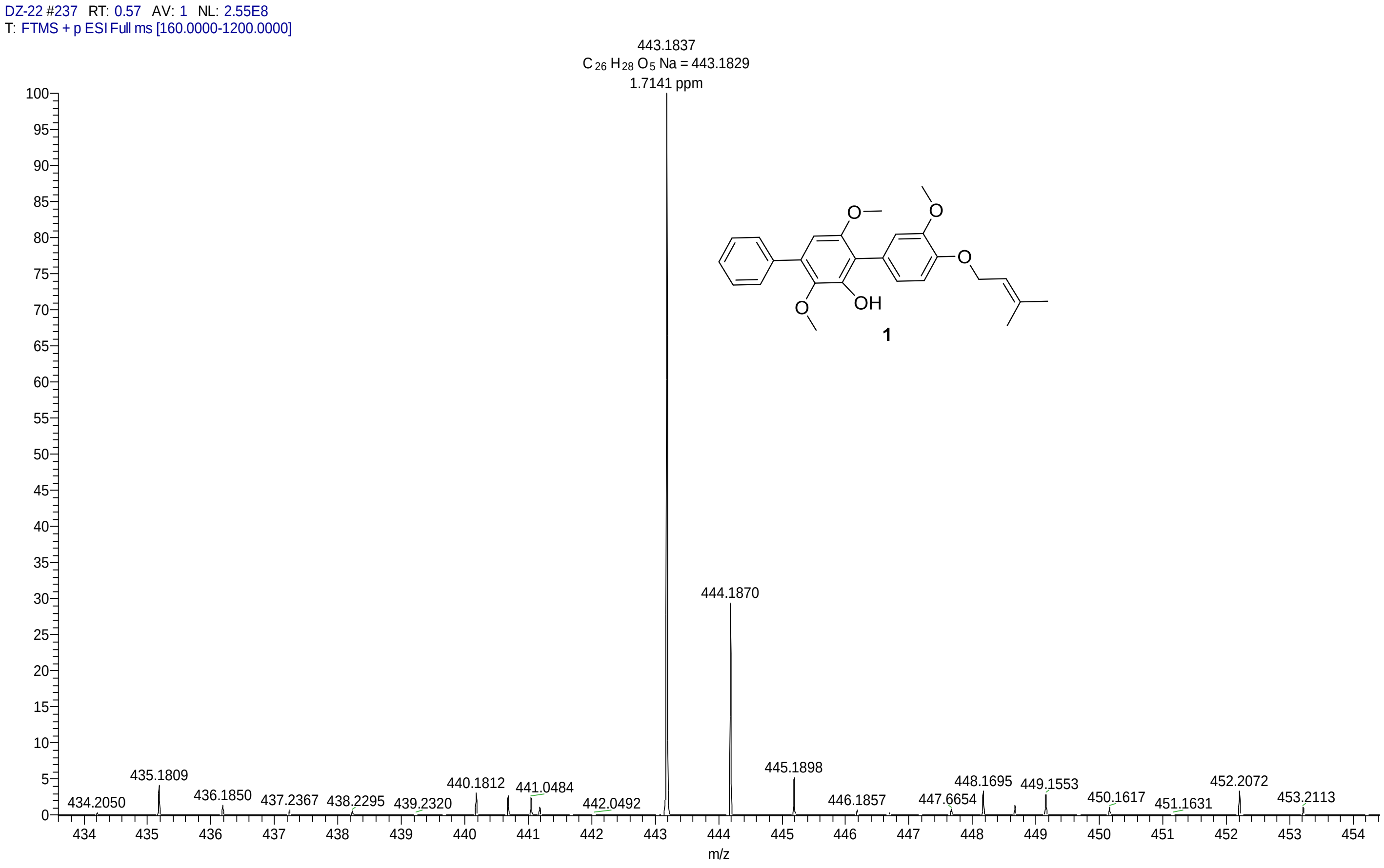

Figure S3. ^{1}H-NMR spectrum of compound **1** in DMSO-d_6

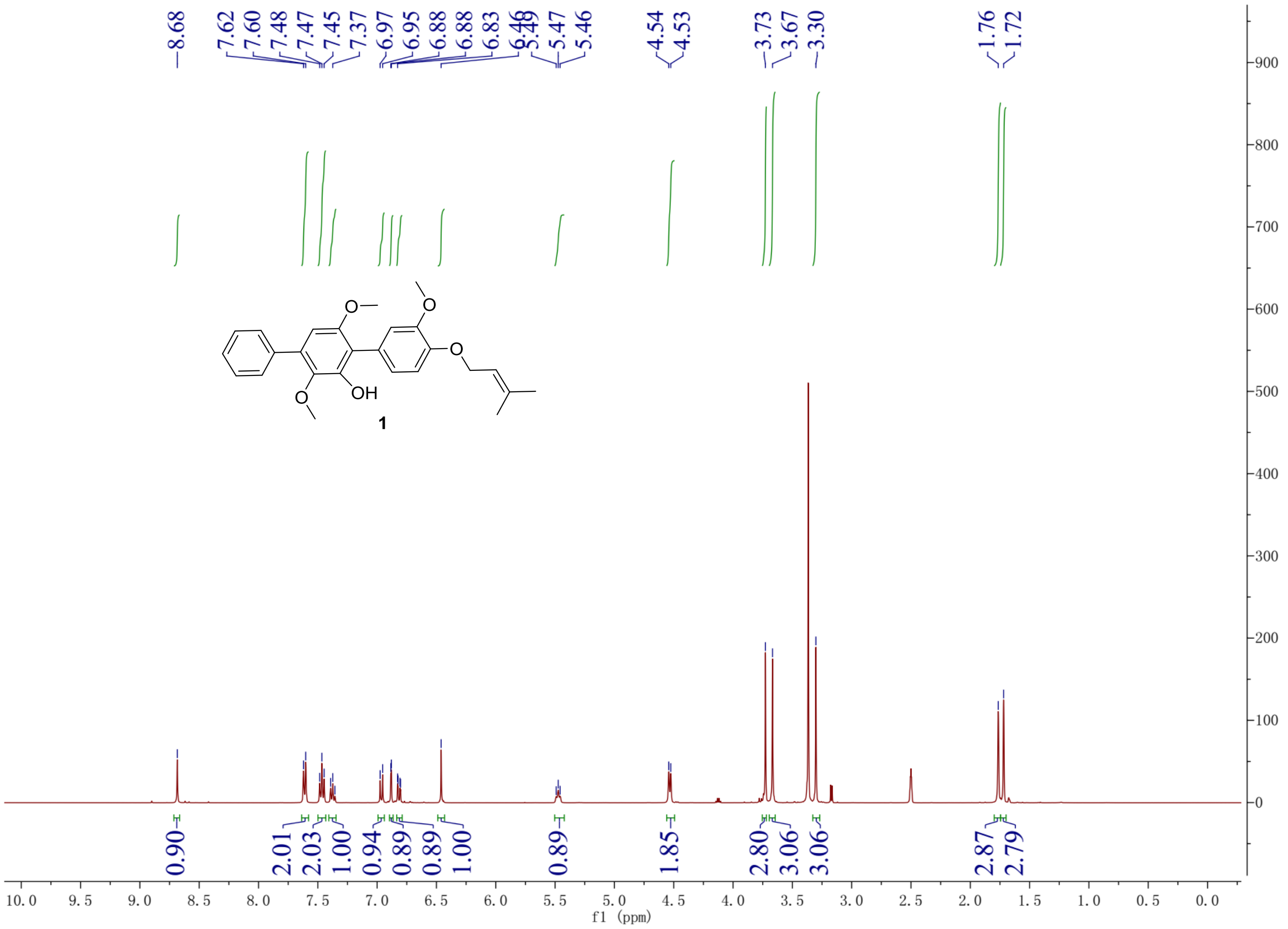

Figure S4. ^{13}C-NMR spectrum of compound **1** in DMSO-d_6

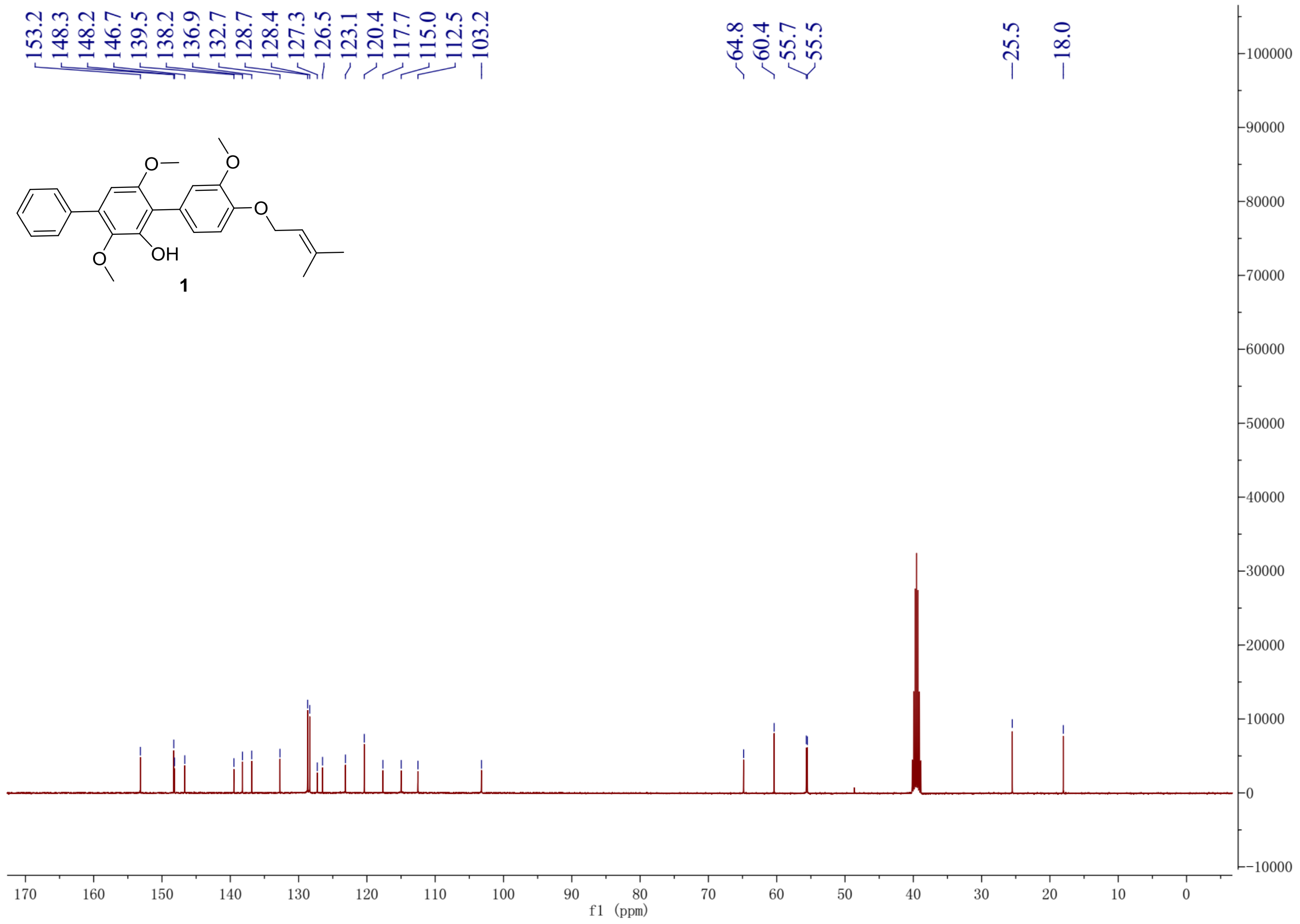

Figure S5. HMQC spectrum of compound **1** in DMSO-d_6

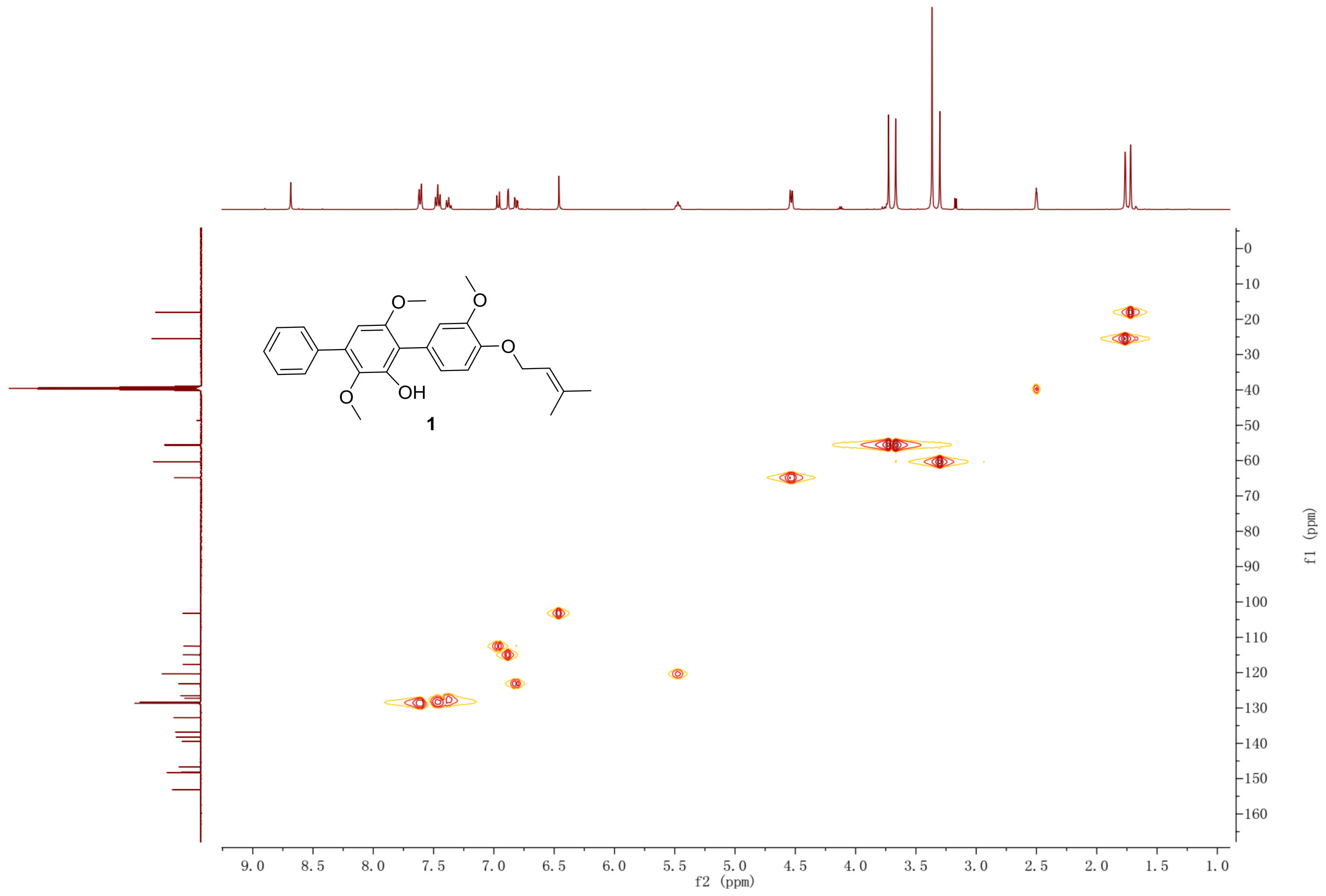

Figure S6. ^1H-^1H COSY spectrum of compound **1** in DMSO-d_6

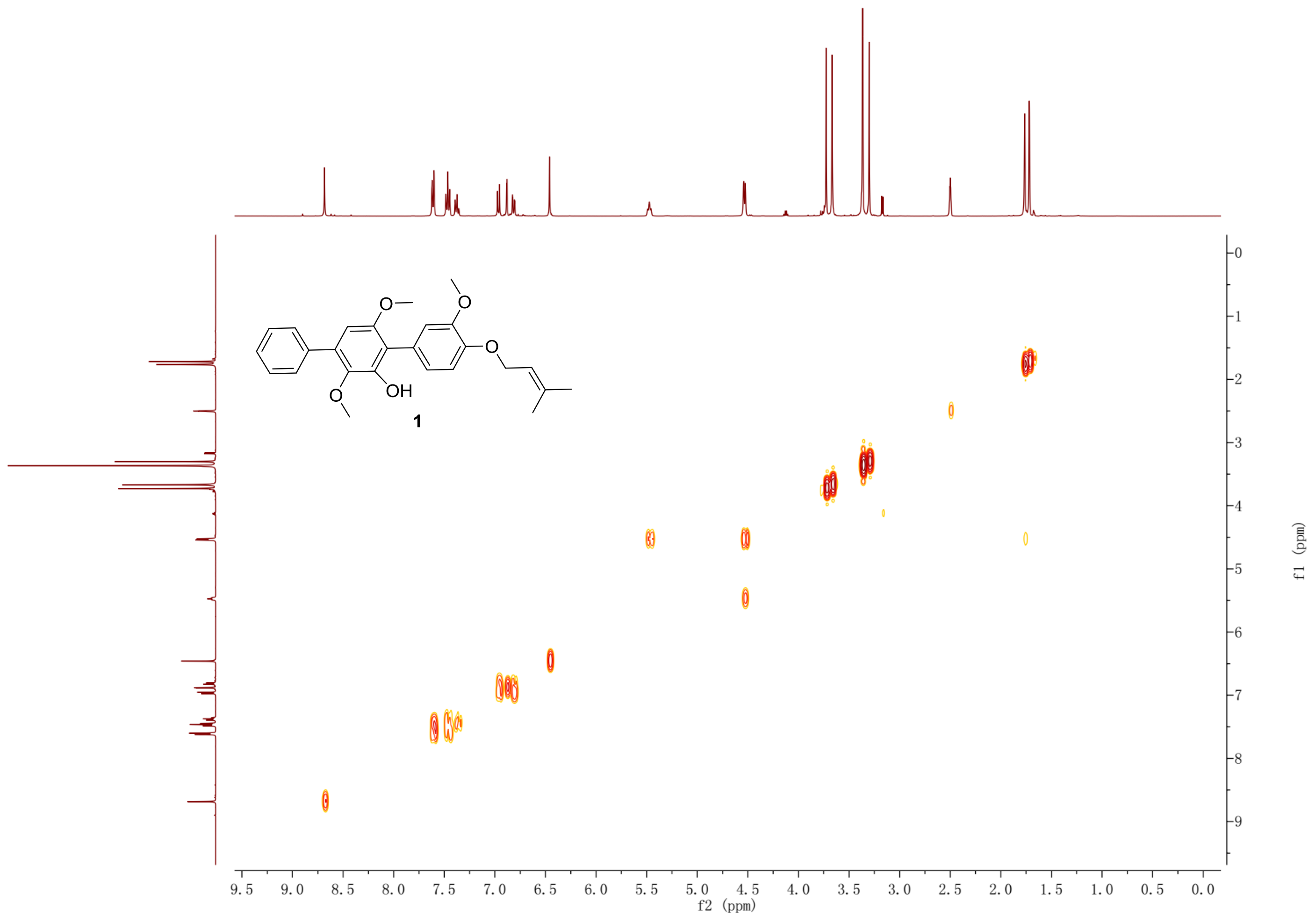

Figure S7. HMBC spectrum of compound **1** in DMSO-d_6

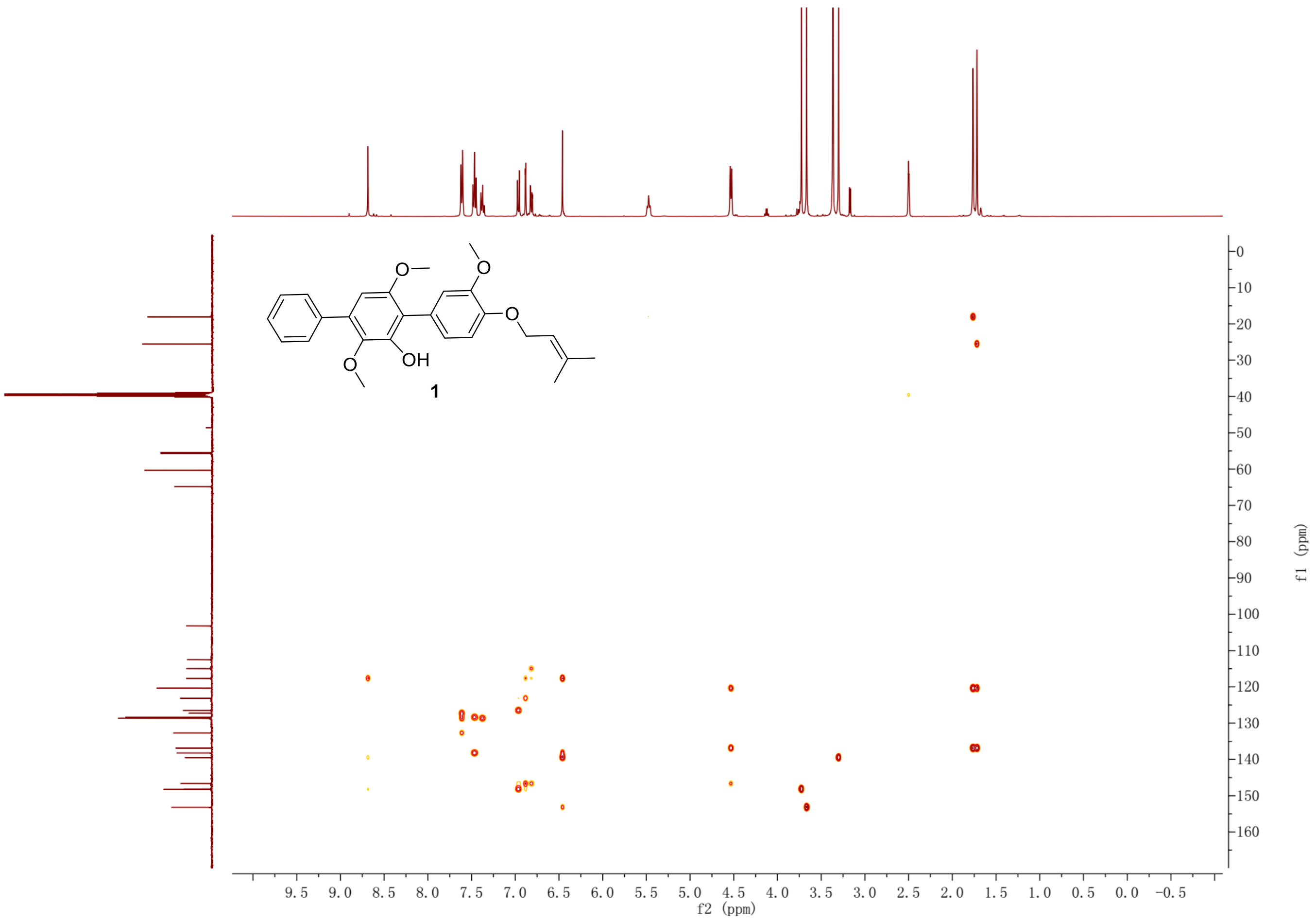

Figure S8. ^{1}H-NMR spectrum of synthesized **15** in DMSO-d_6

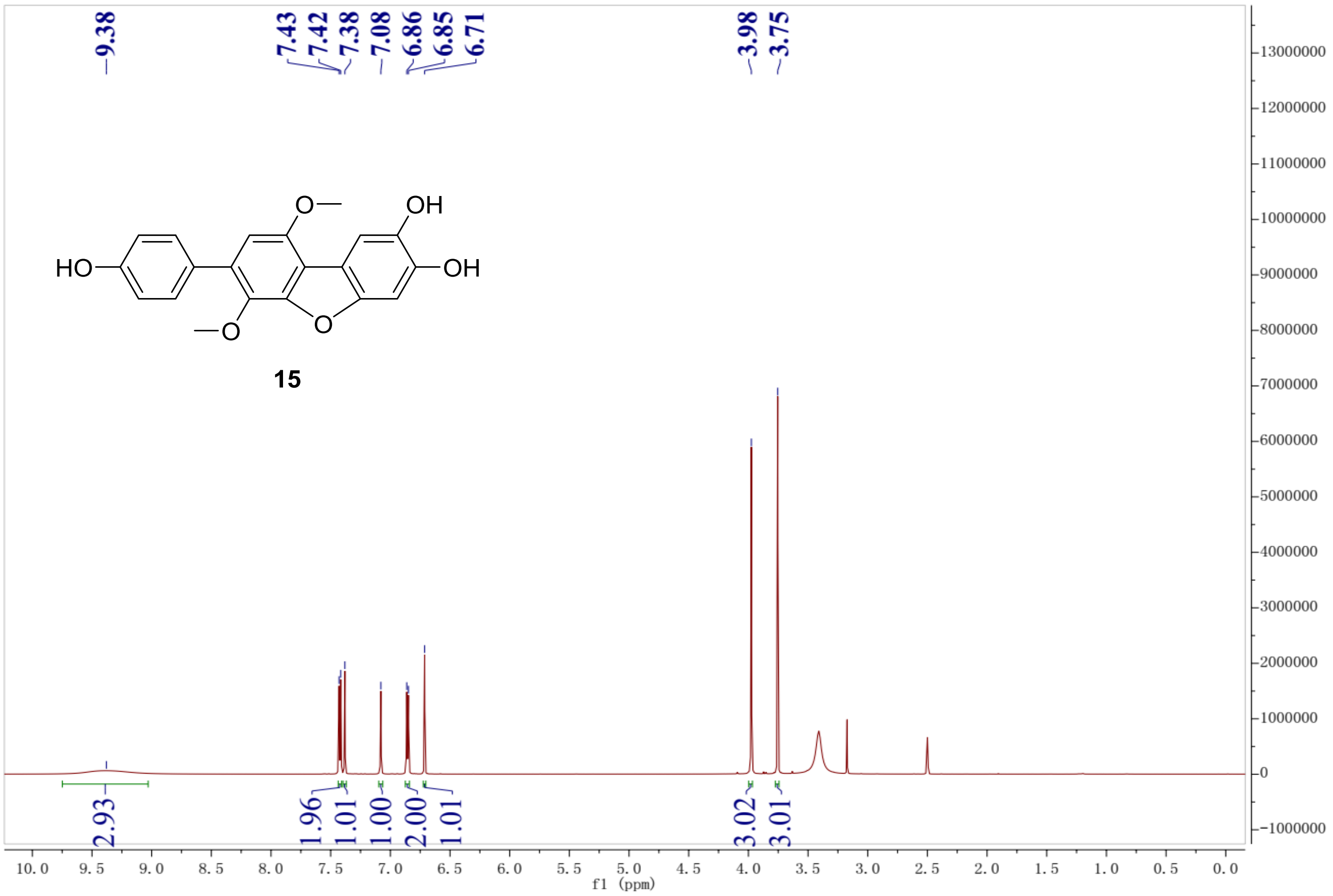

Figure S9. ^{13}C-NMR spectrum of synthesized **15** in DMSO-d_6

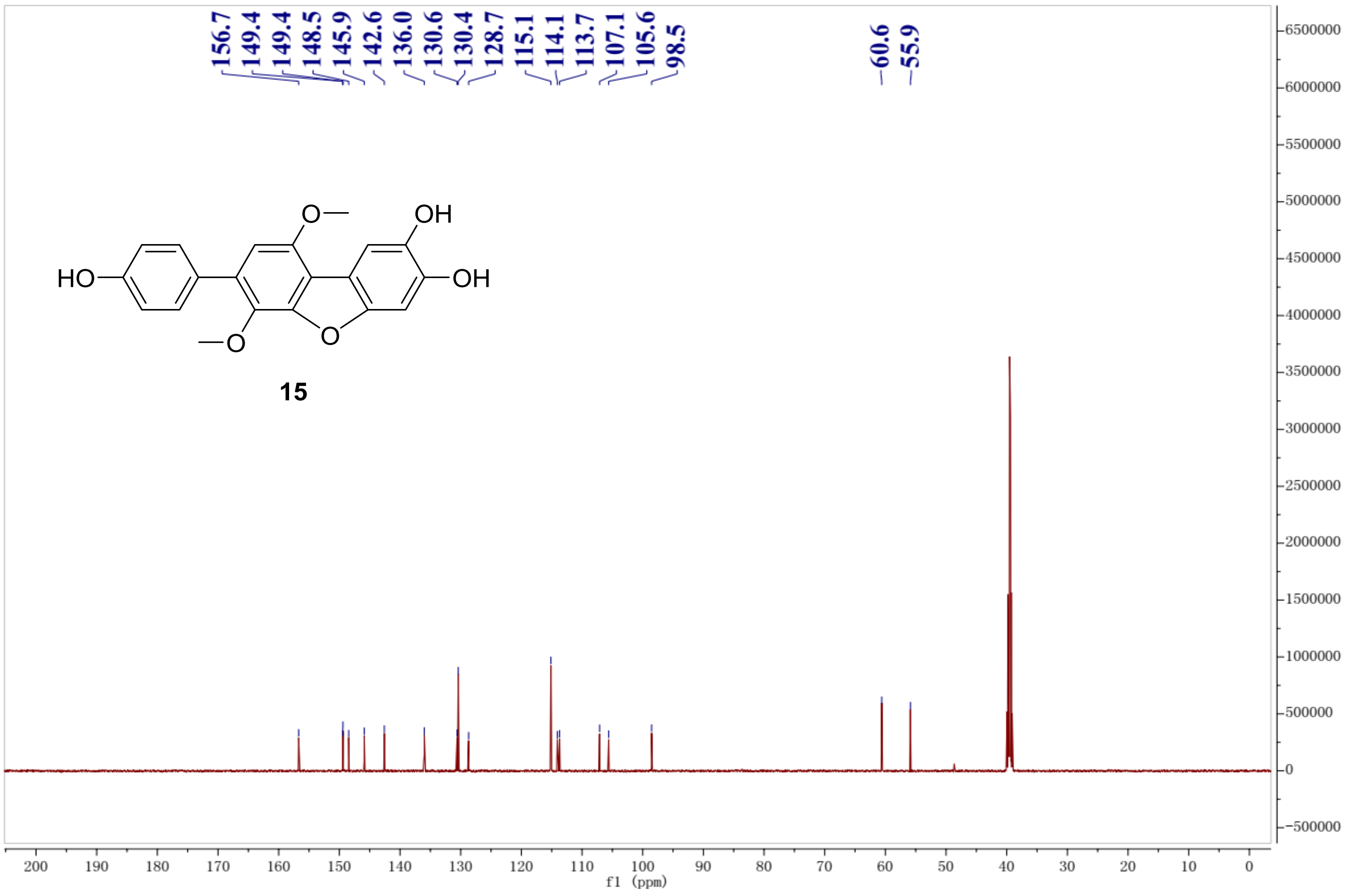

Figure S10. ^{1}H-NMR spectrum of synthesized **16** in DMSO-d_6

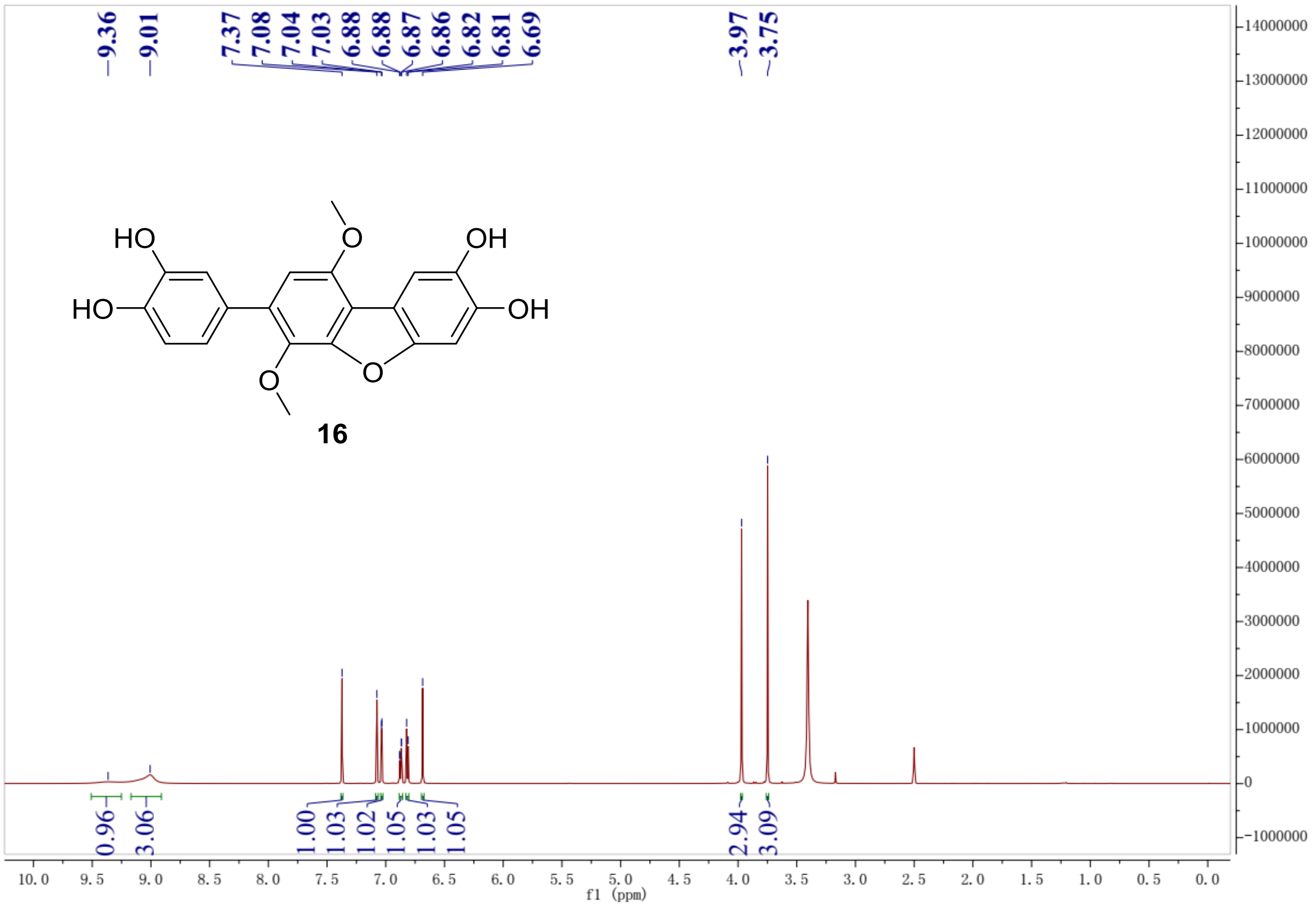

Figure S11. ^{13}C-NMR spectrum of synthesized **16** in DMSO-d_6

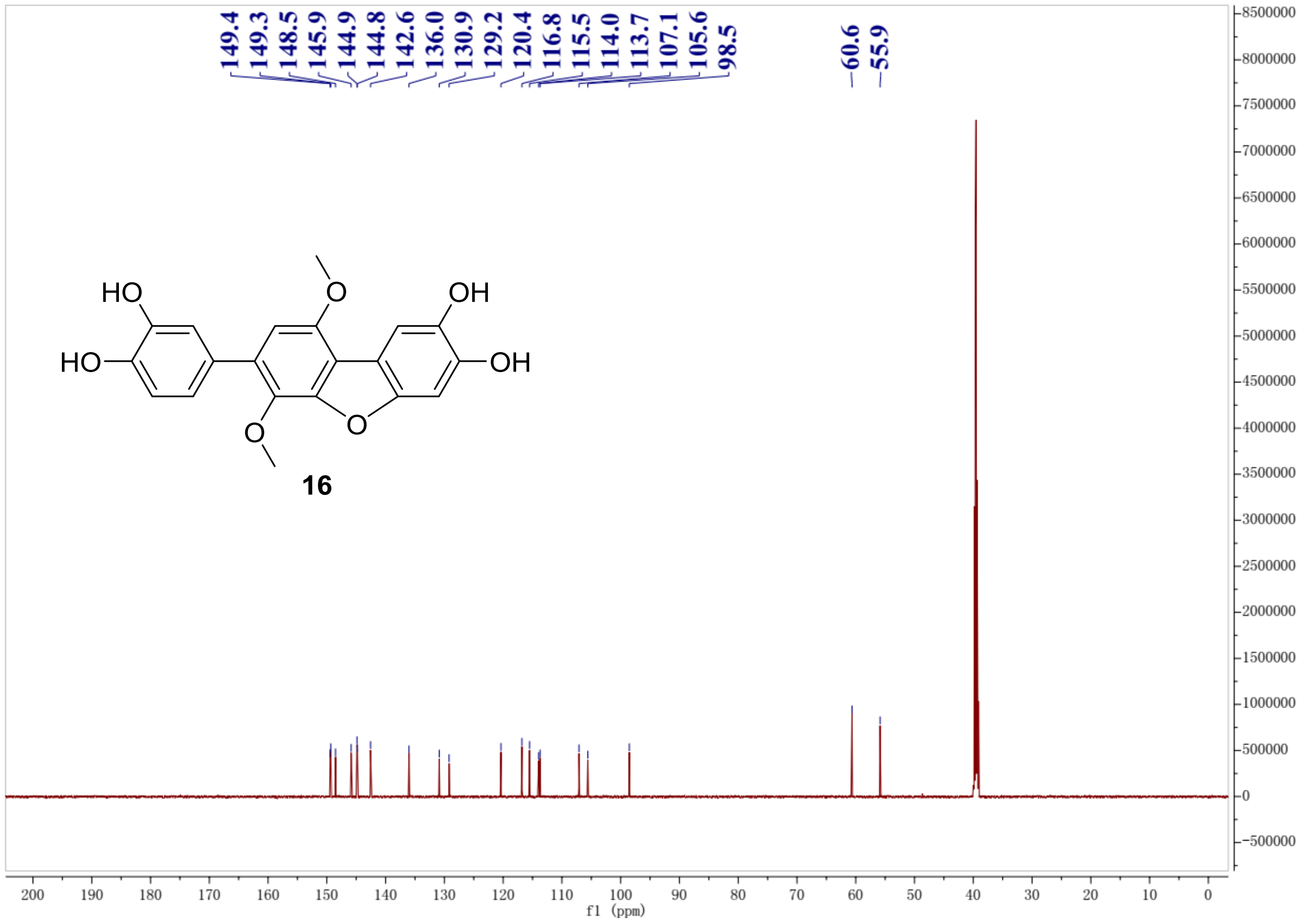

Figure S12. ^{1}H-NMR spectrum of compound **17** in DMSO-d_6

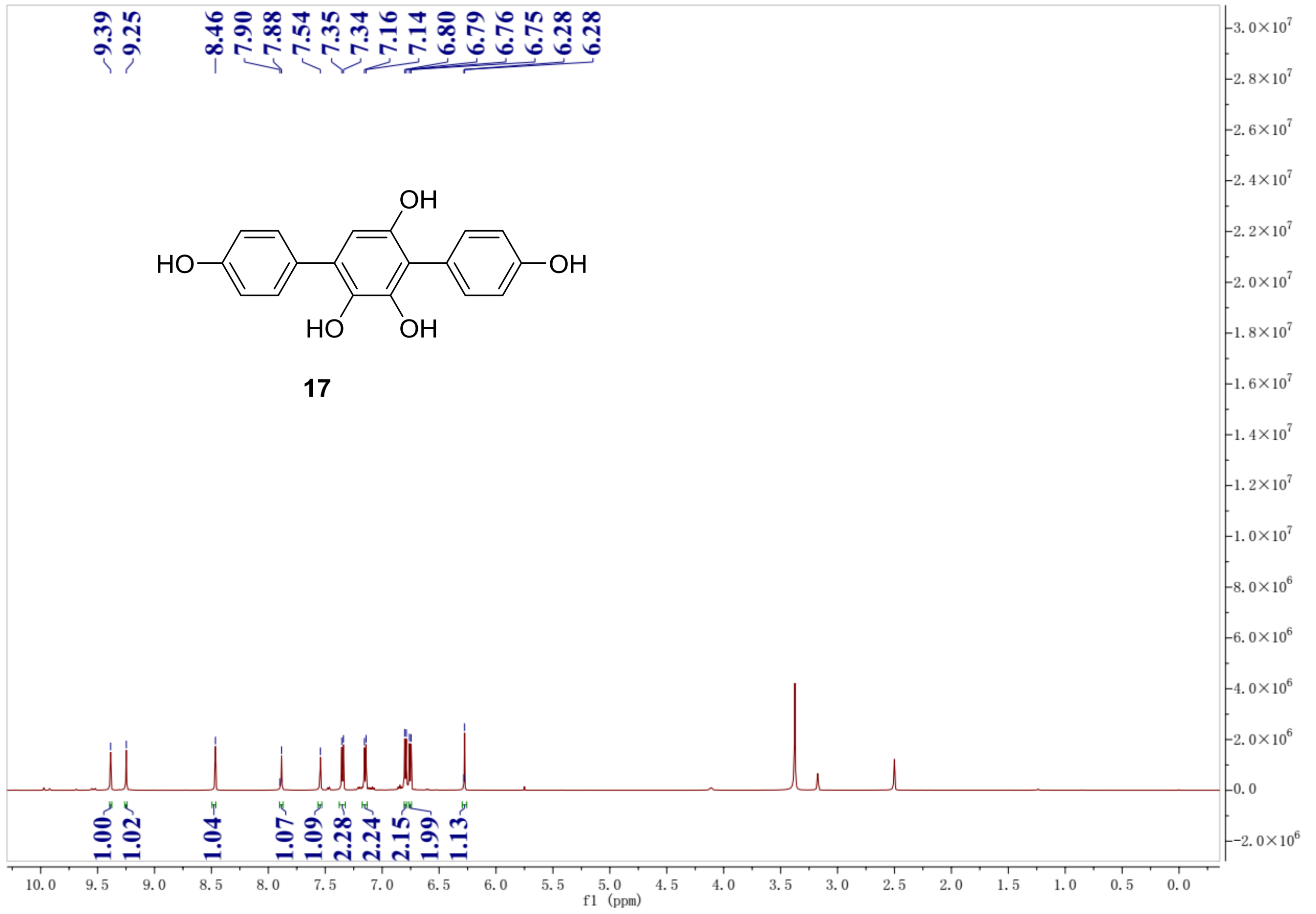

Figure S13. ^{13}C-NMR spectrum of compound **17** in DMSO-d_6

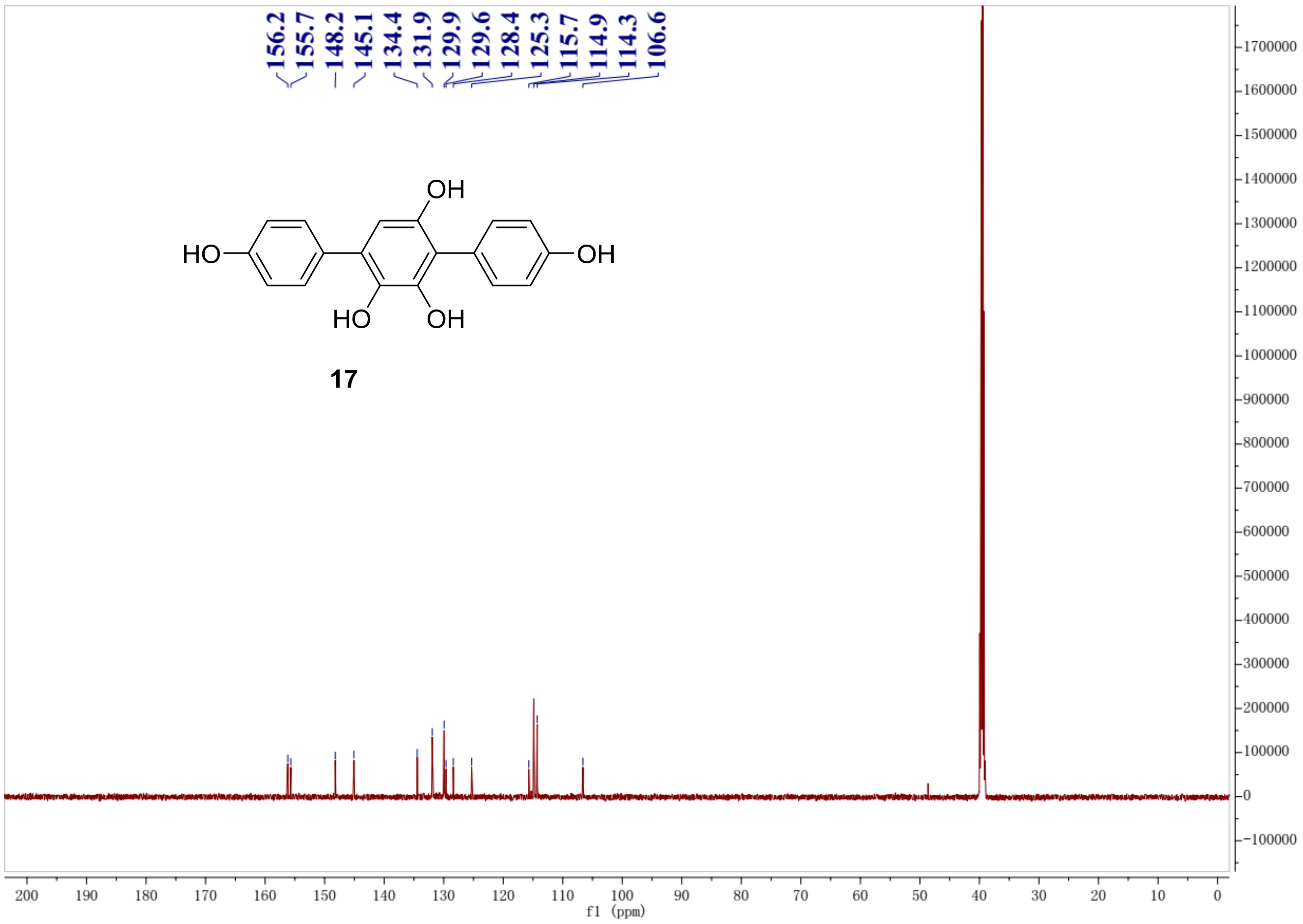

Figure S14. HRESIMS spectrum of compound **18**

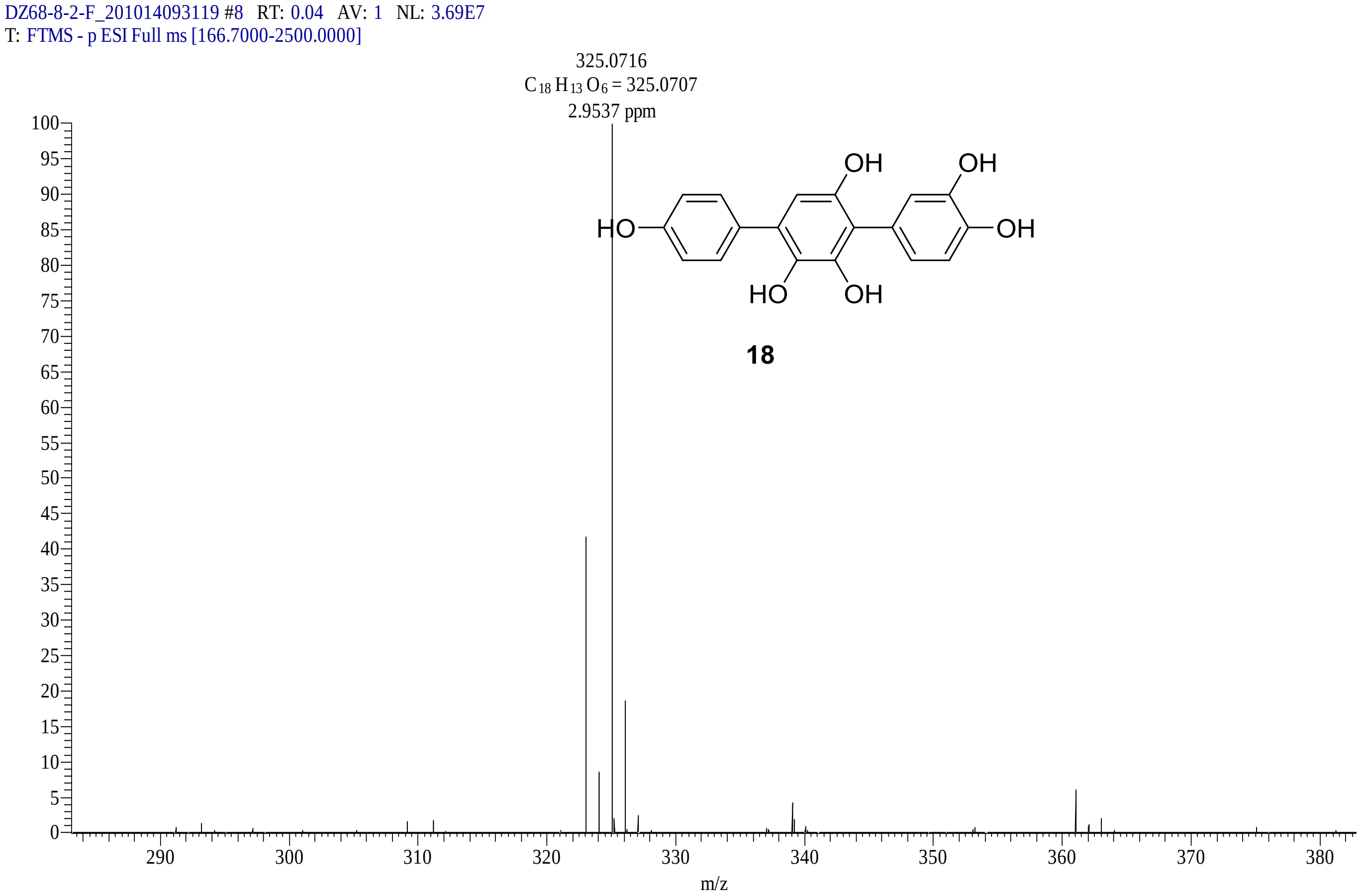

Figure S15. ^{1}H-NMR spectrum of compound **18** in DMSO-d_6

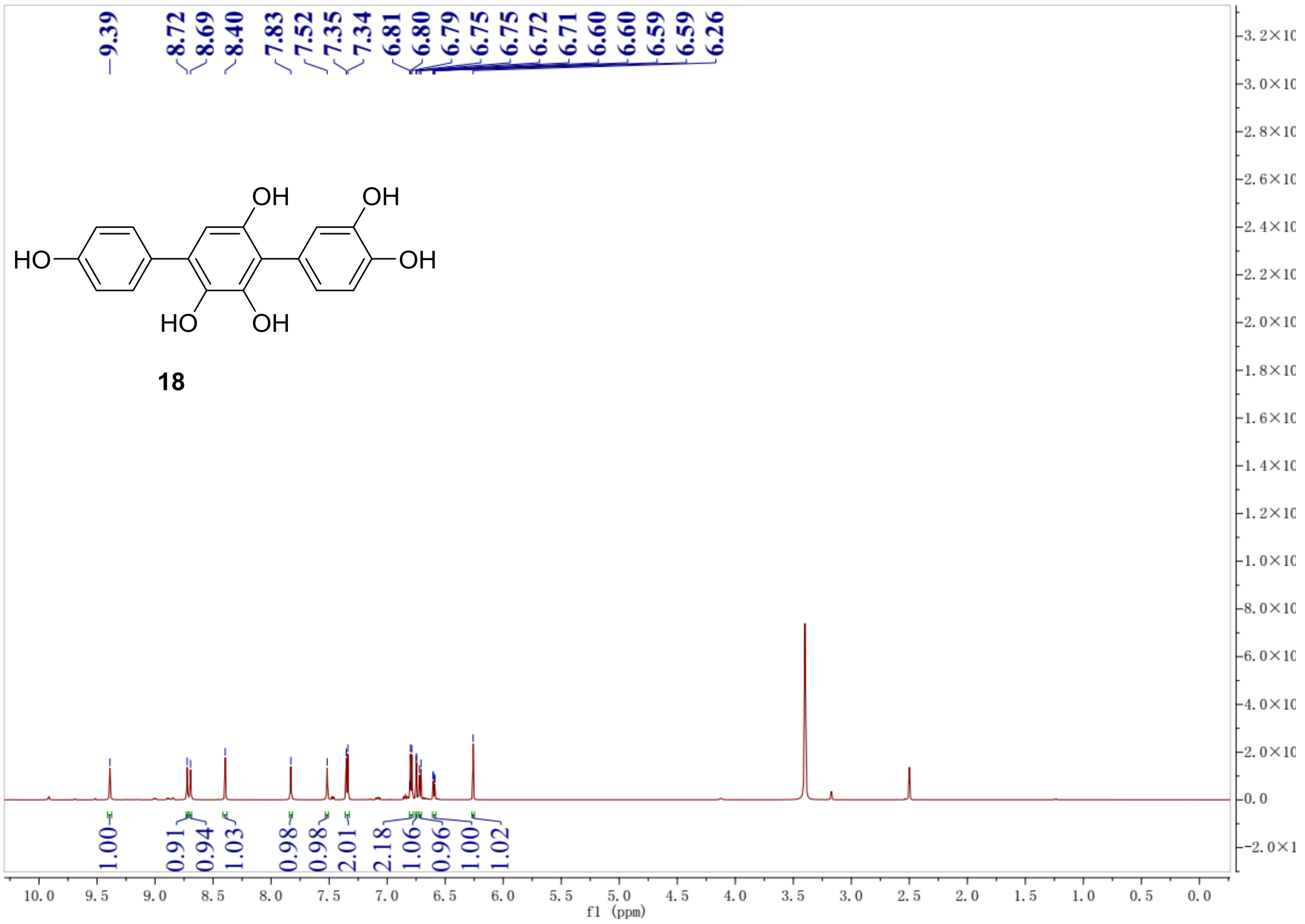

Figure S16. ^{13}C-NMR spectrum of compound **18** in DMSO-d_6

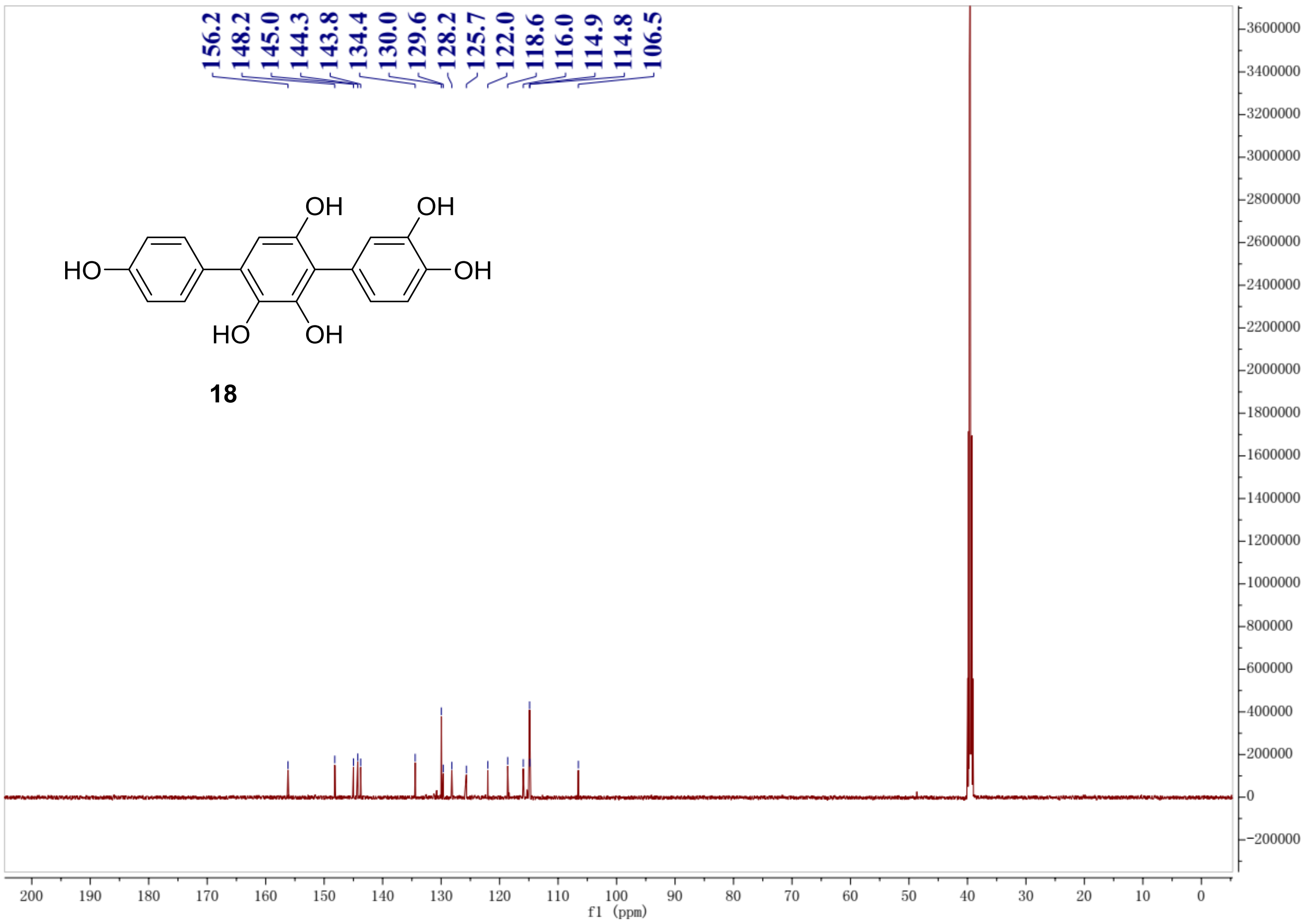

Figure S17. HRESIMS spectrum of compound **19**

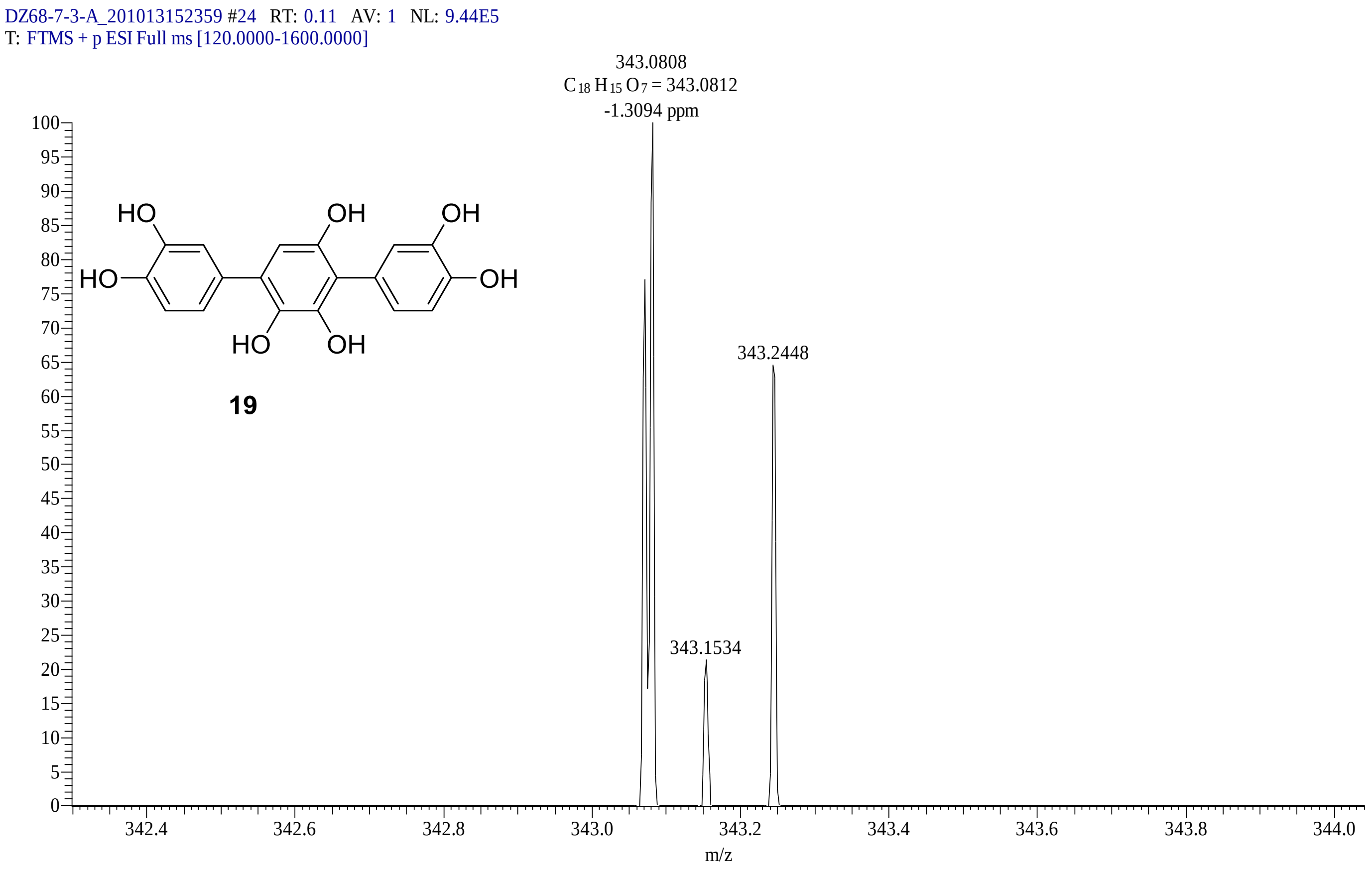

Figure S18. ^{1}H-NMR spectrum of compound **19** in DMSO-d_6

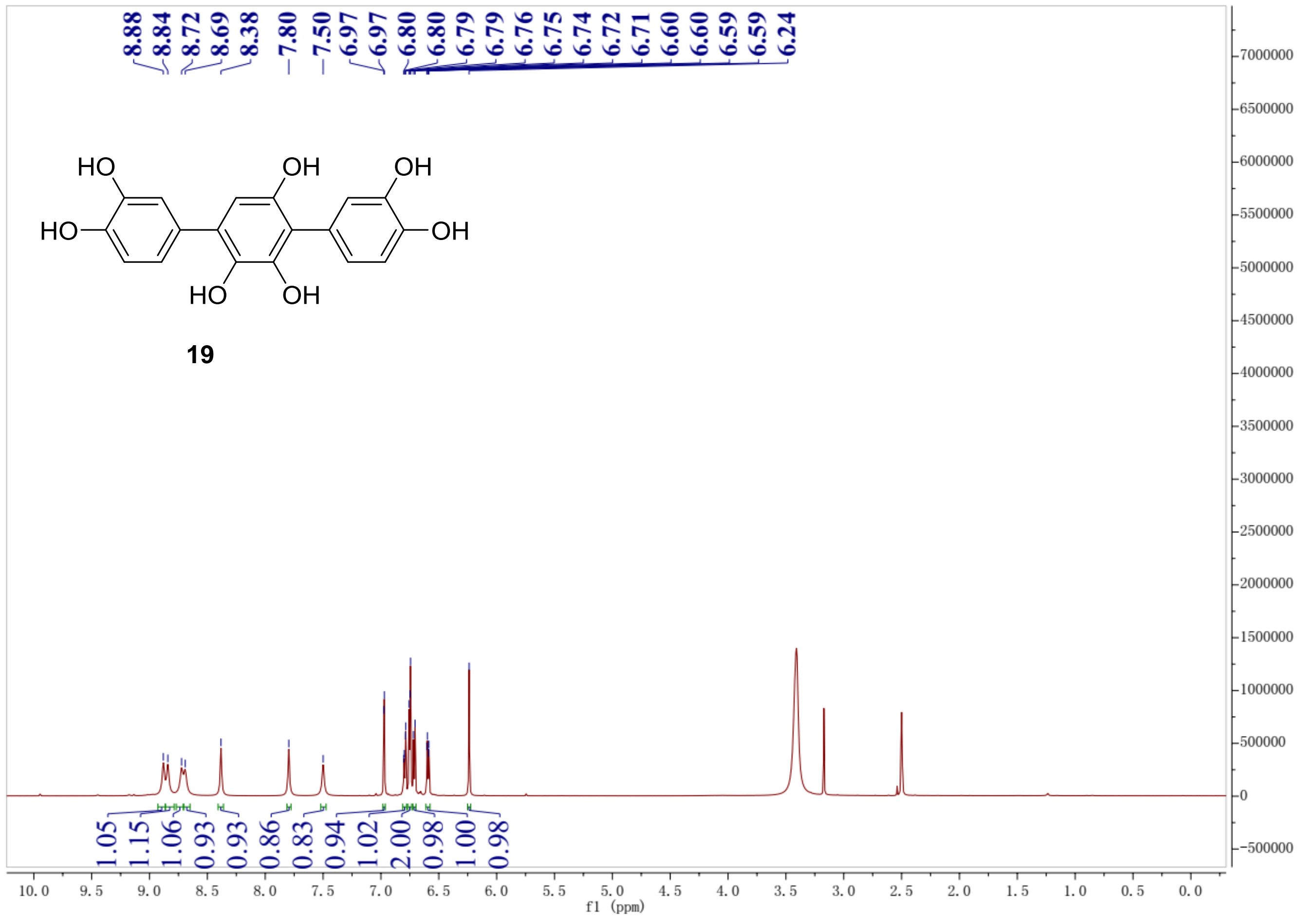

Figure S19. ^{13}C-NMR spectrum of compound **19** in DMSO-d_6

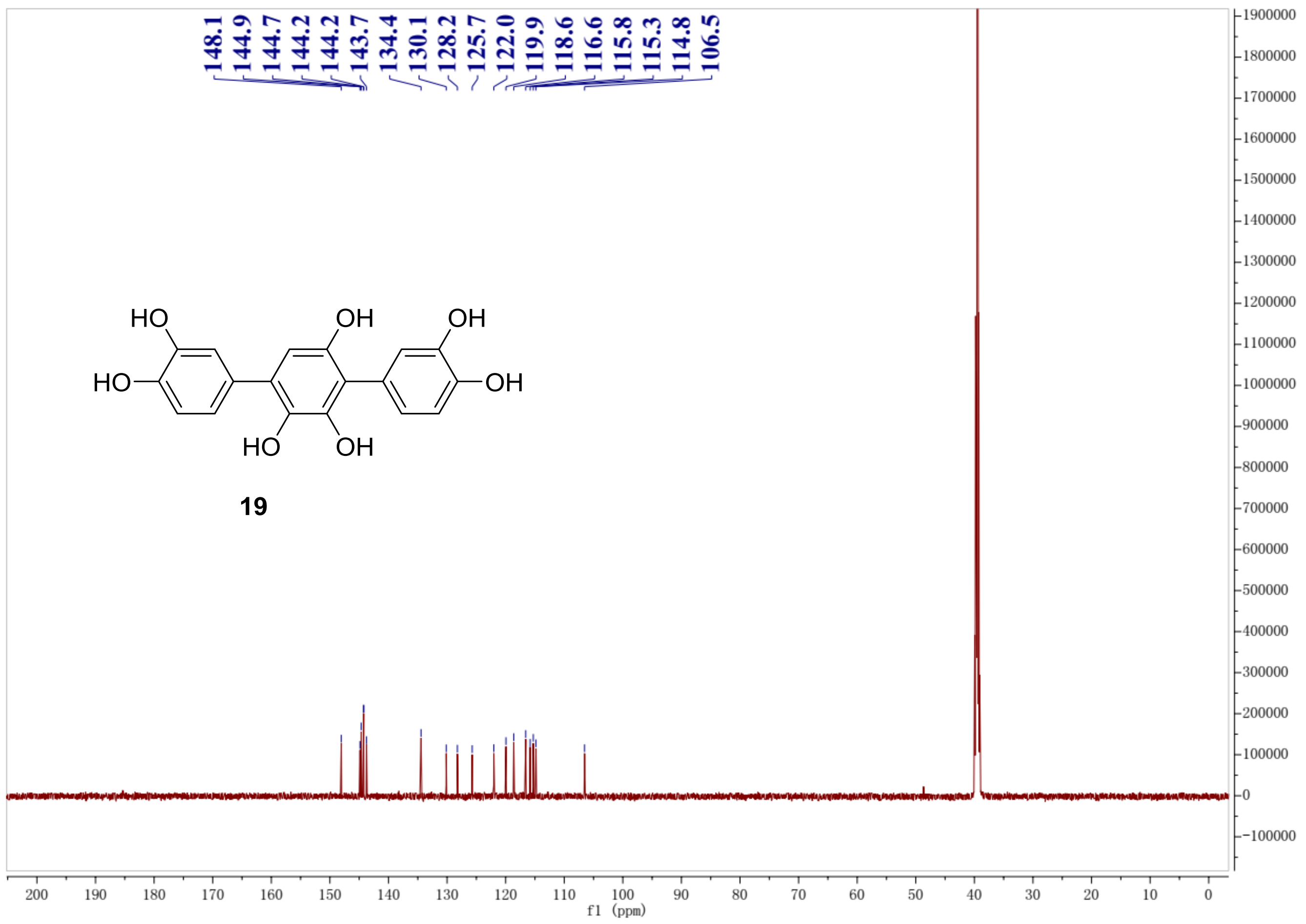

Figure S20. ^{1}H-NMR spectrum of compound **20** in DMSO-d_6

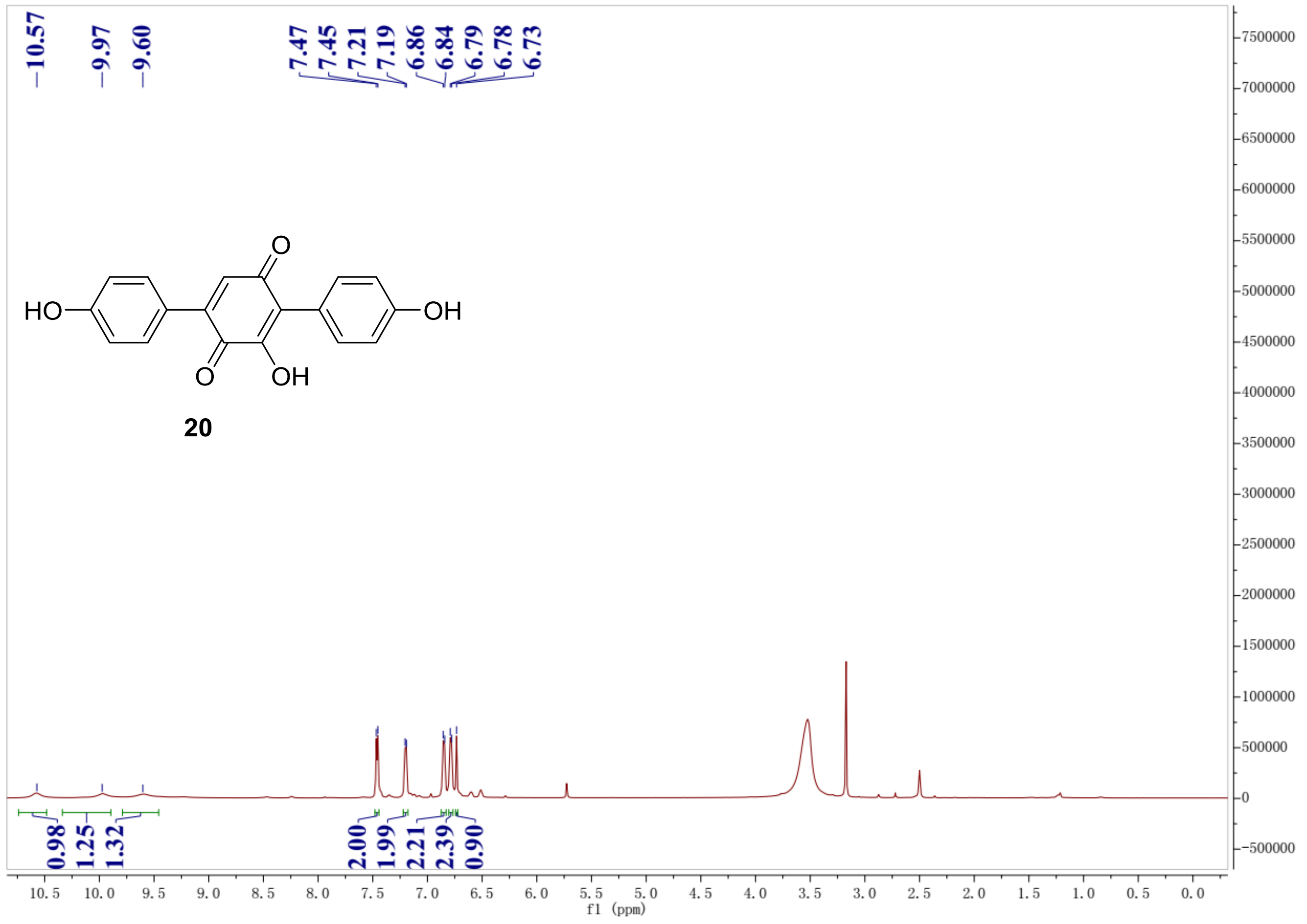

Figure S21. ^{13}C-NMR spectrum of compound **20** in DMSO-d_6

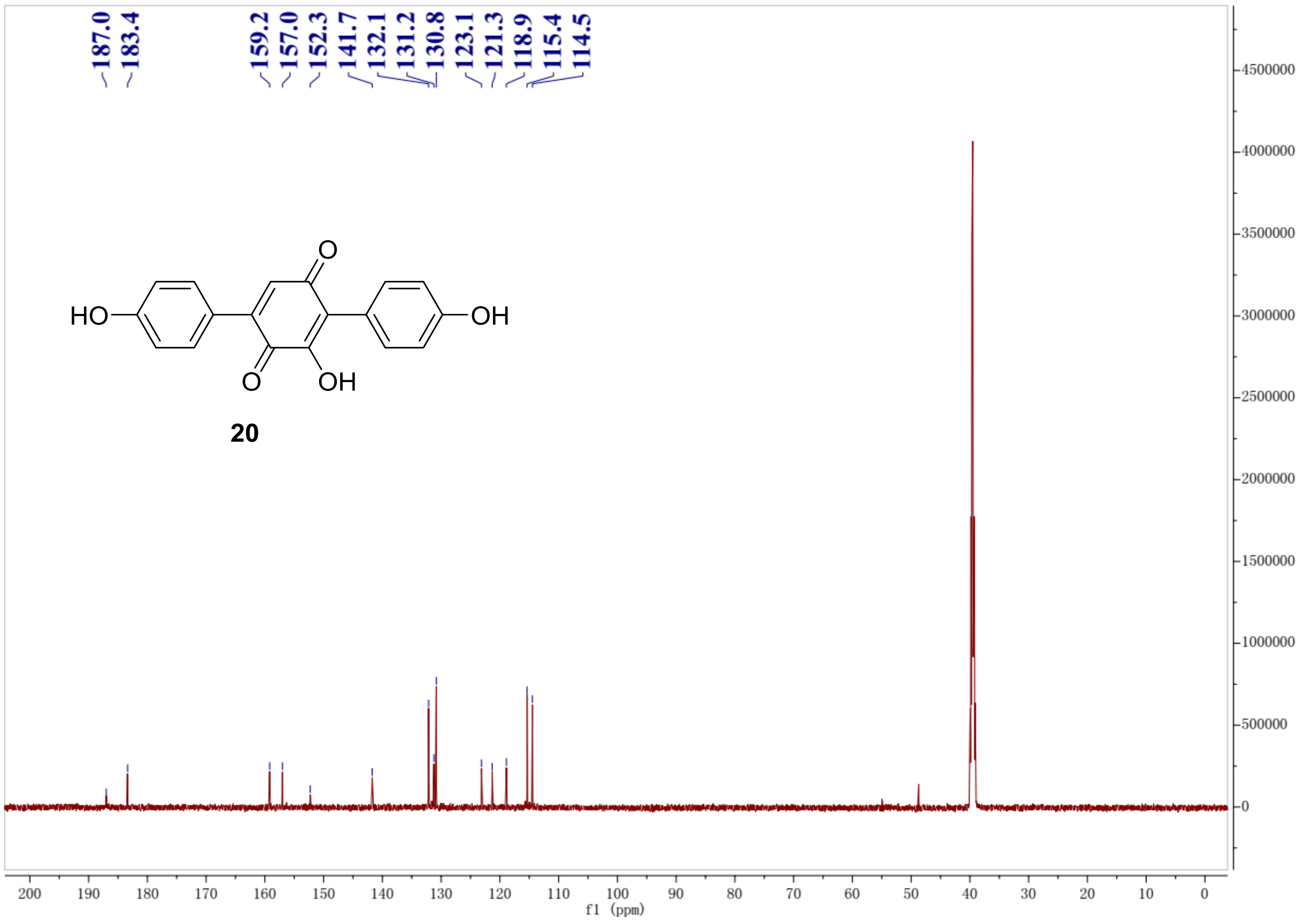

Figure S22. HRESIMS spectrum of compound **21**

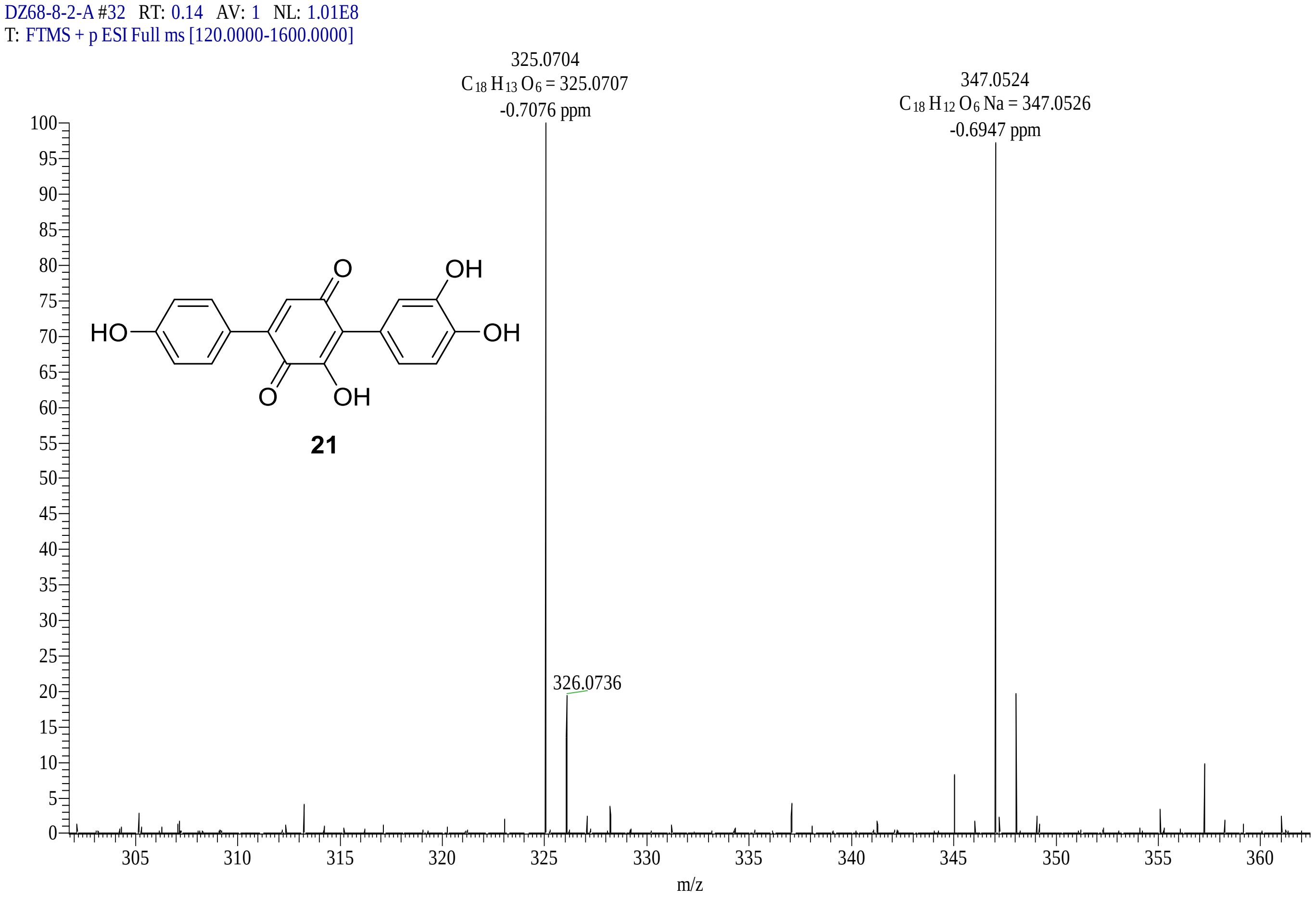

Figure S23. ^{1}H-NMR spectrum of compound **21** in DMSO-d_6

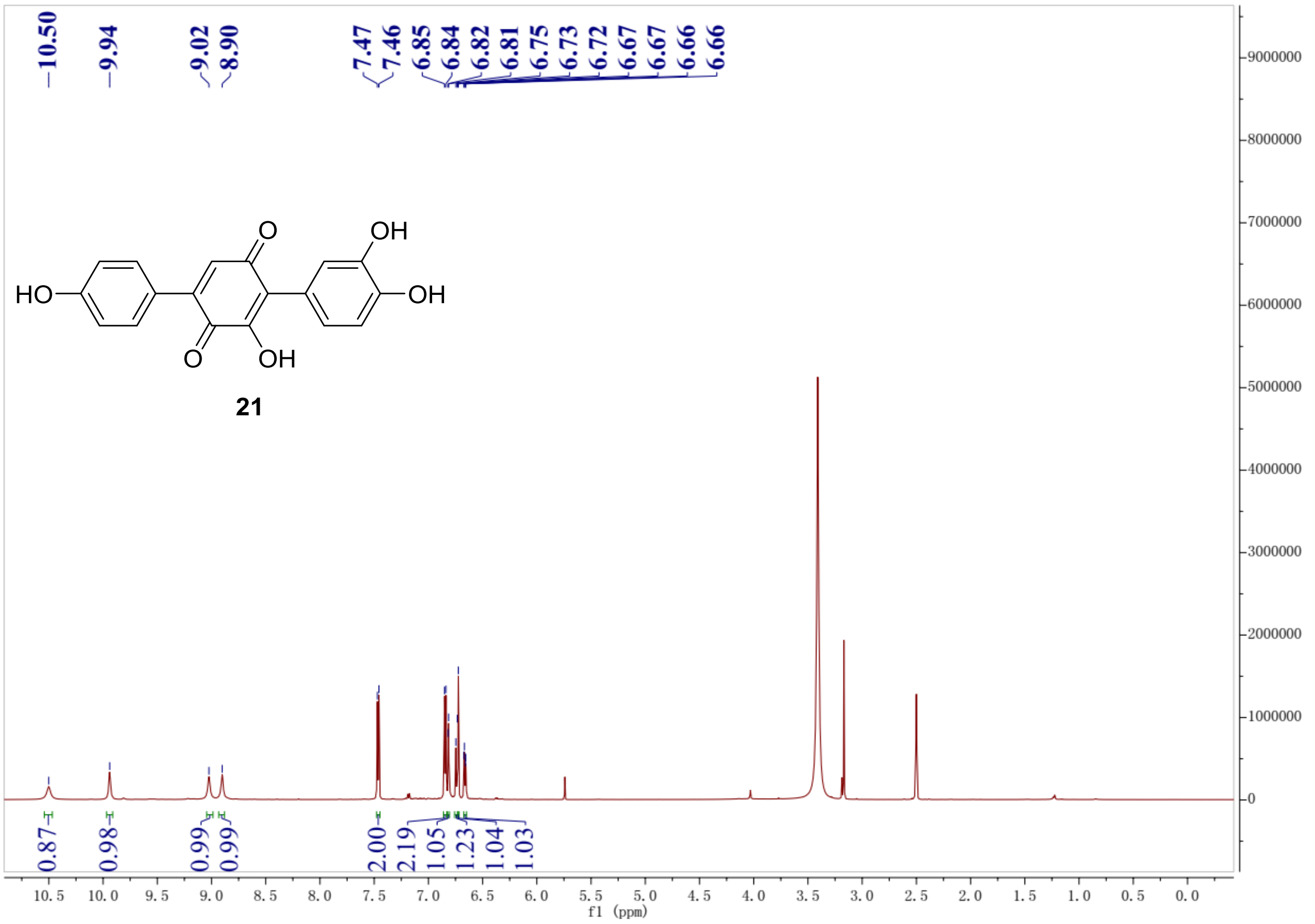

Figure S24. ^{13}C-NMR spectrum of compound **21** in DMSO-d_6

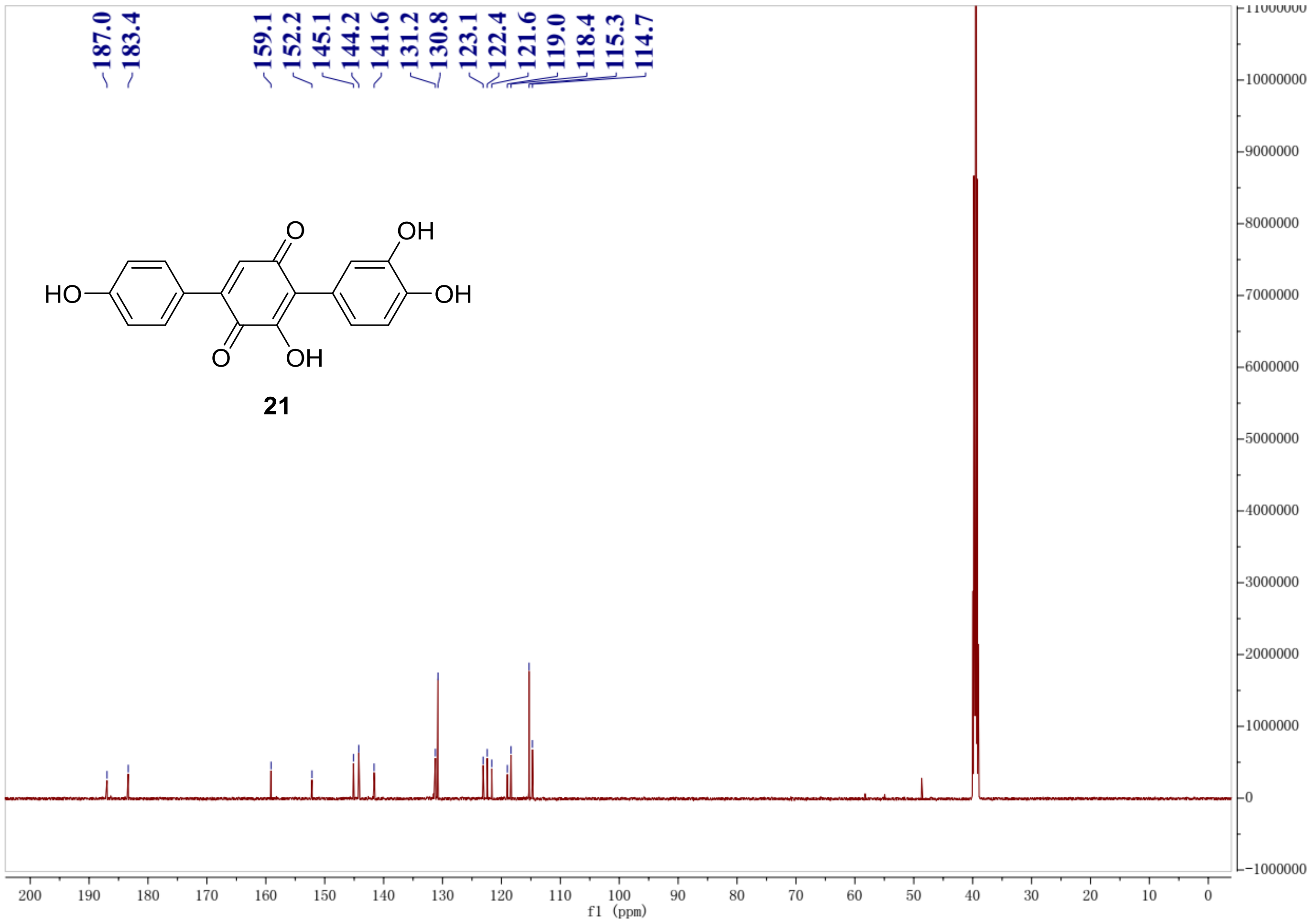

Figure S25. HRESIMS spectrum of compound **22**

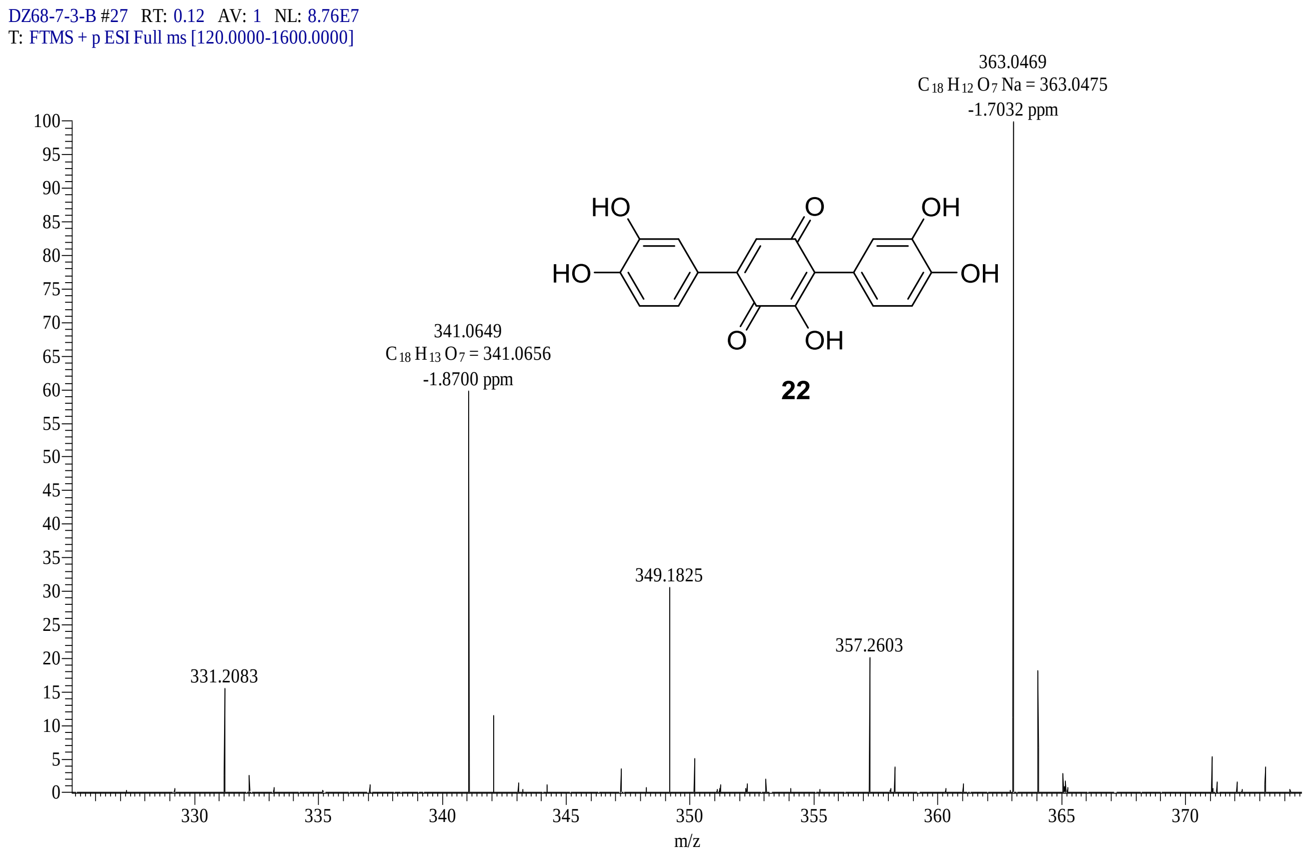

Figure S26. ^{1}H-NMR spectrum of compound **22** in DMSO-d_6

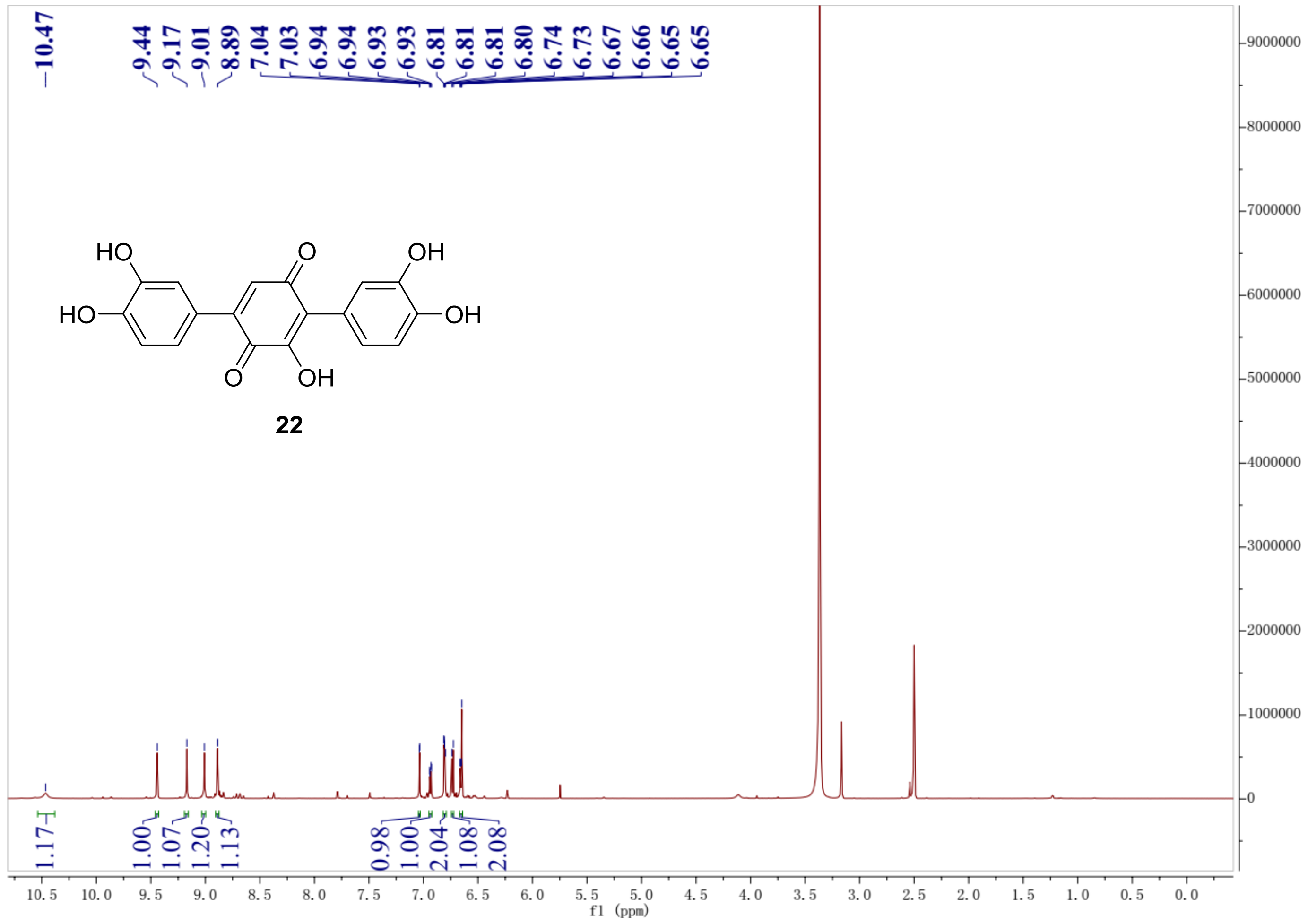

Figure S27. ^{13}C-NMR spectrum of compound **22** in DMSO-d_6

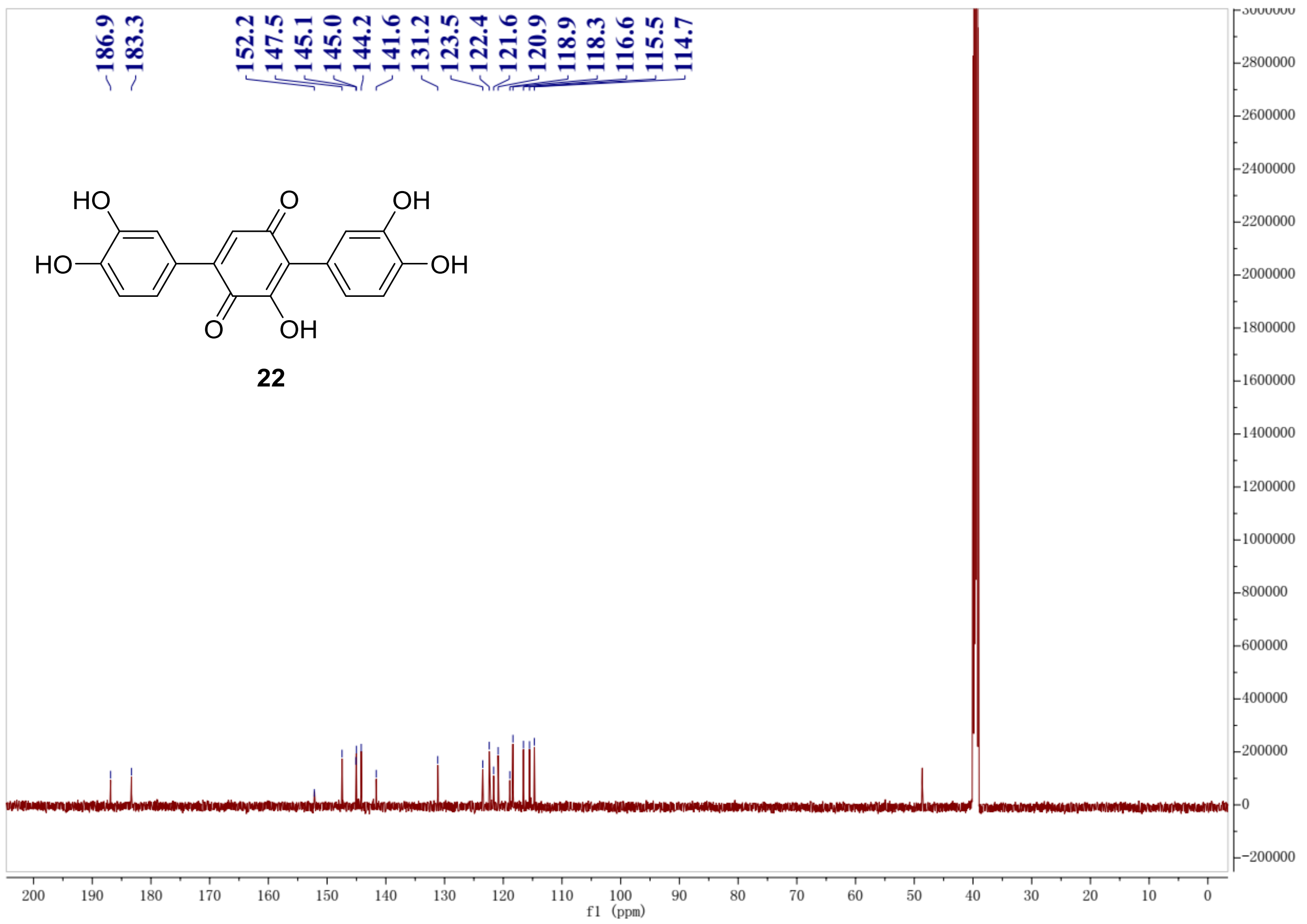

Figure S28. HRESIMS spectrum of compound **23**

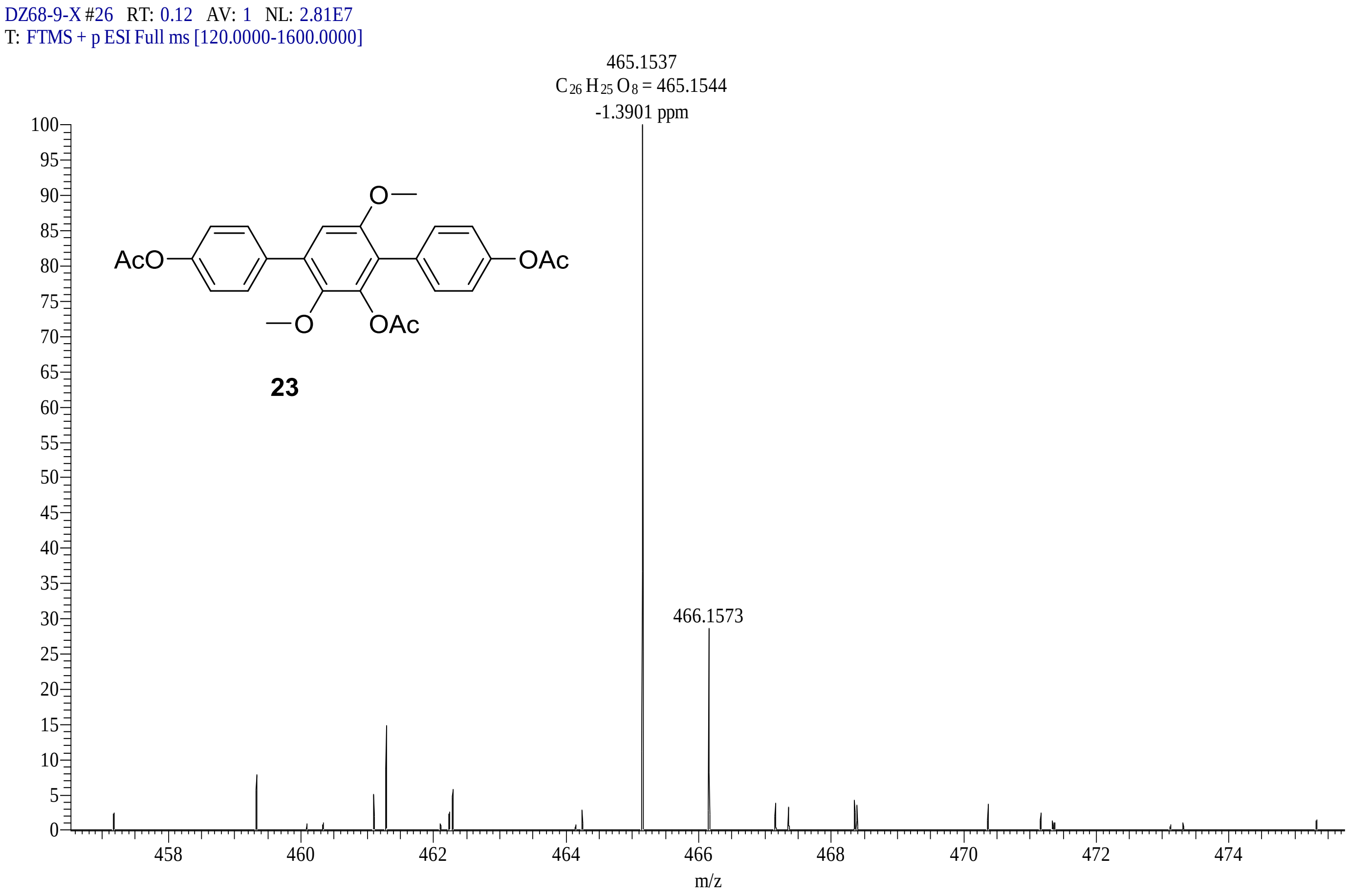

Figure S29. ^{1}H-NMR spectrum of compound **23** in DMSO-d_6

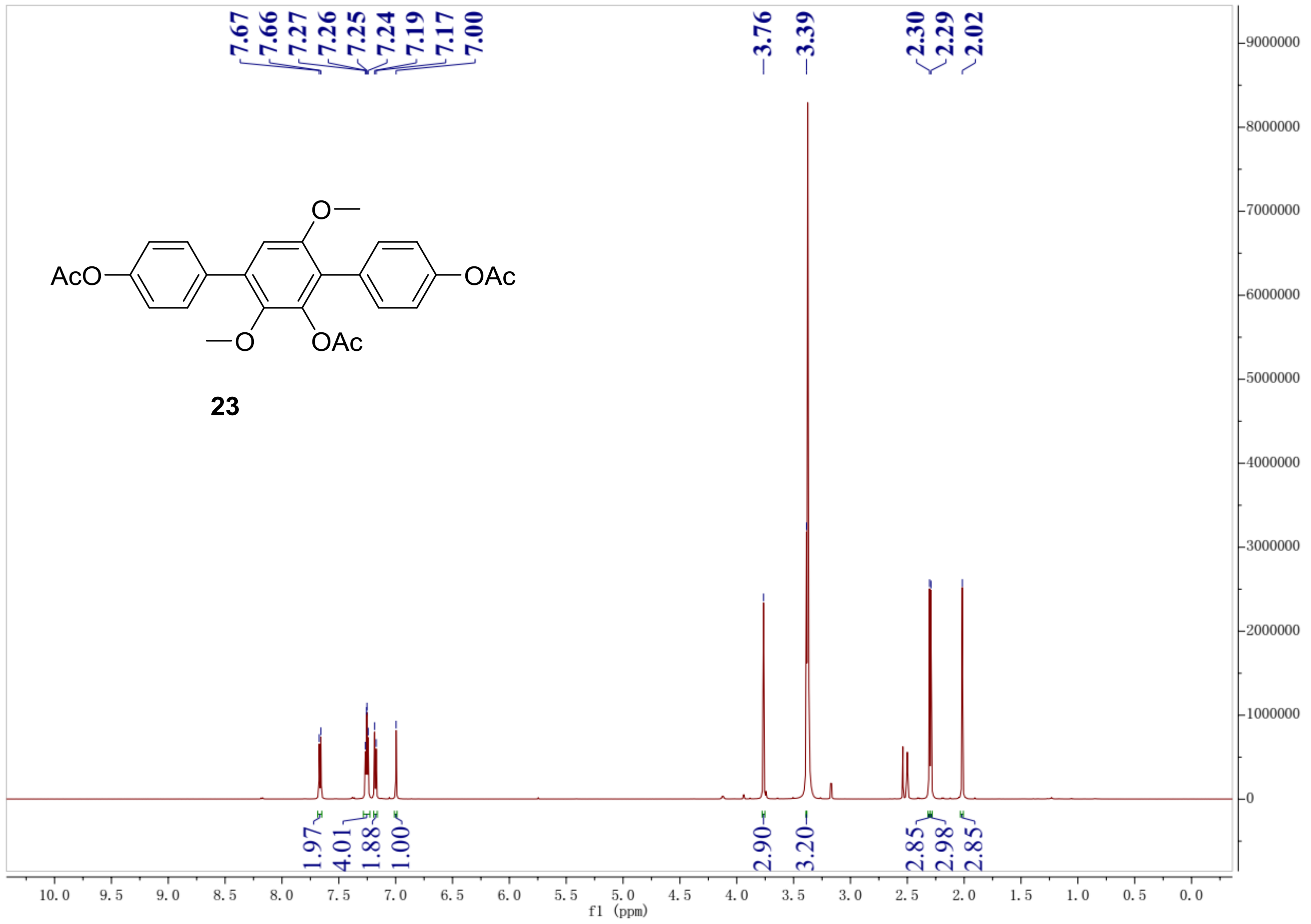

Figure S30. ^{13}C-NMR spectrum of compound **23** in DMSO-d_6

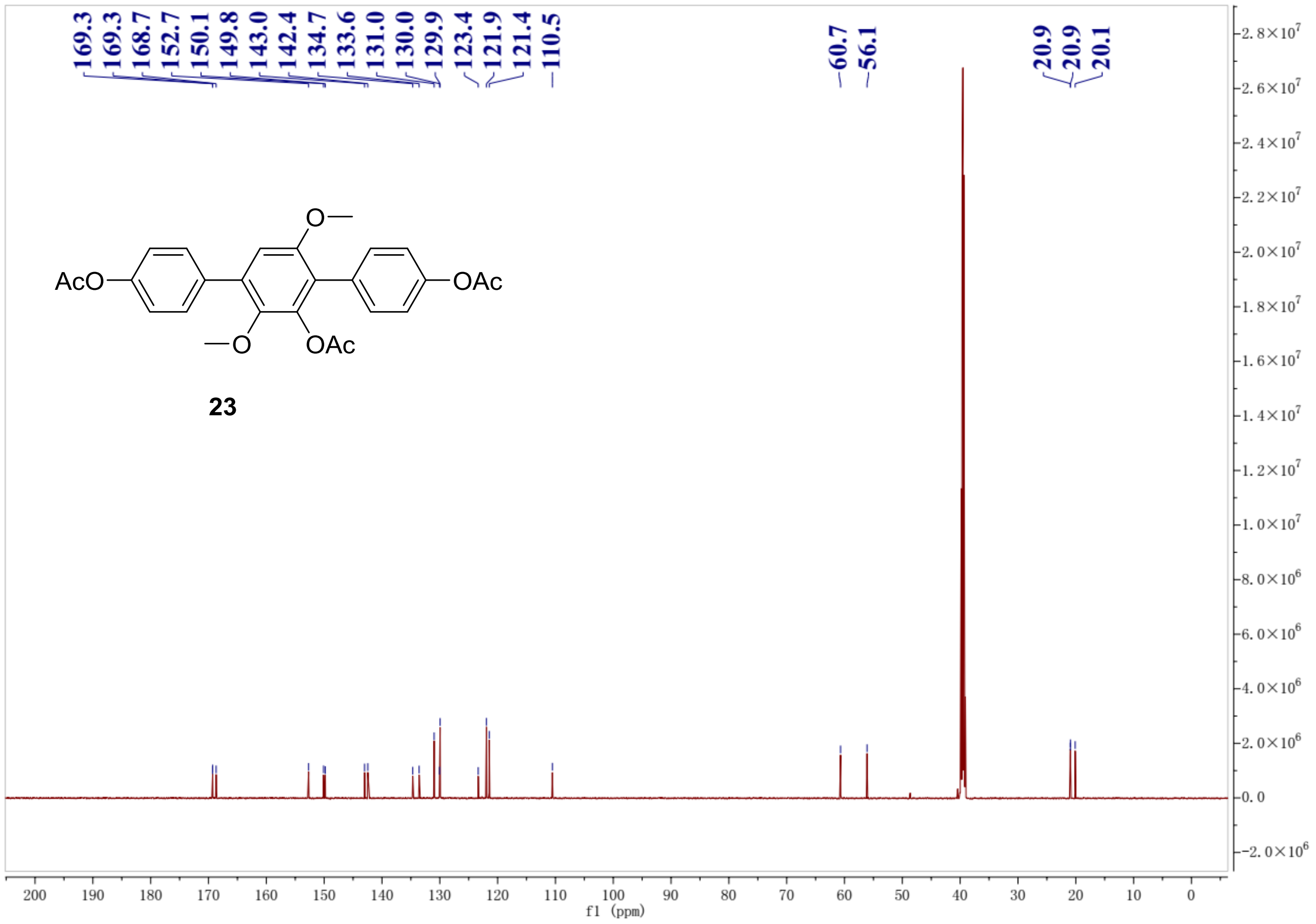

Figure S31. ^{1}H-NMR spectrum of compound **24** in $CDCl_3$

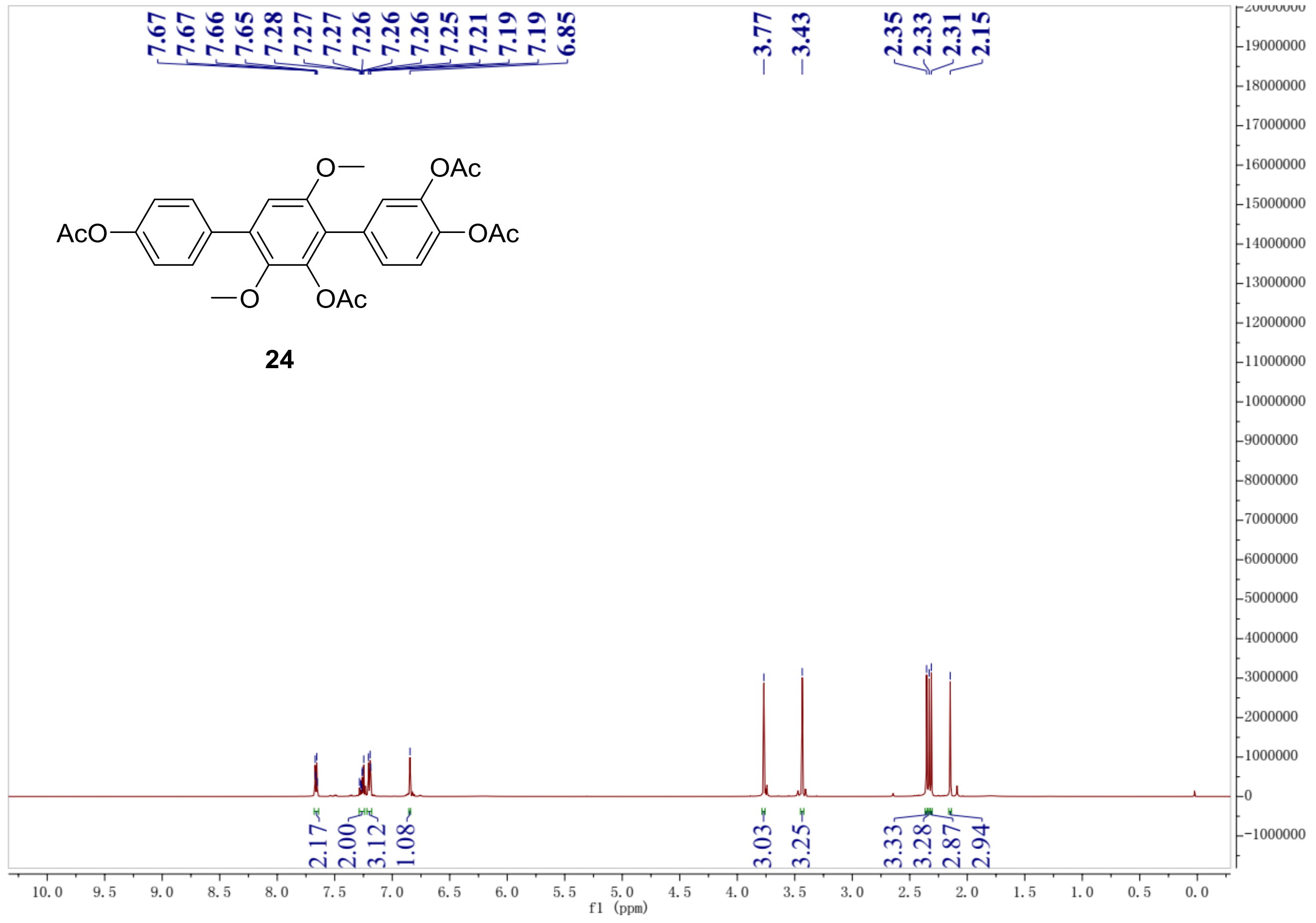

Figure S32. ^{13}C-NMR spectrum of compound **24** in $CDCl_3$

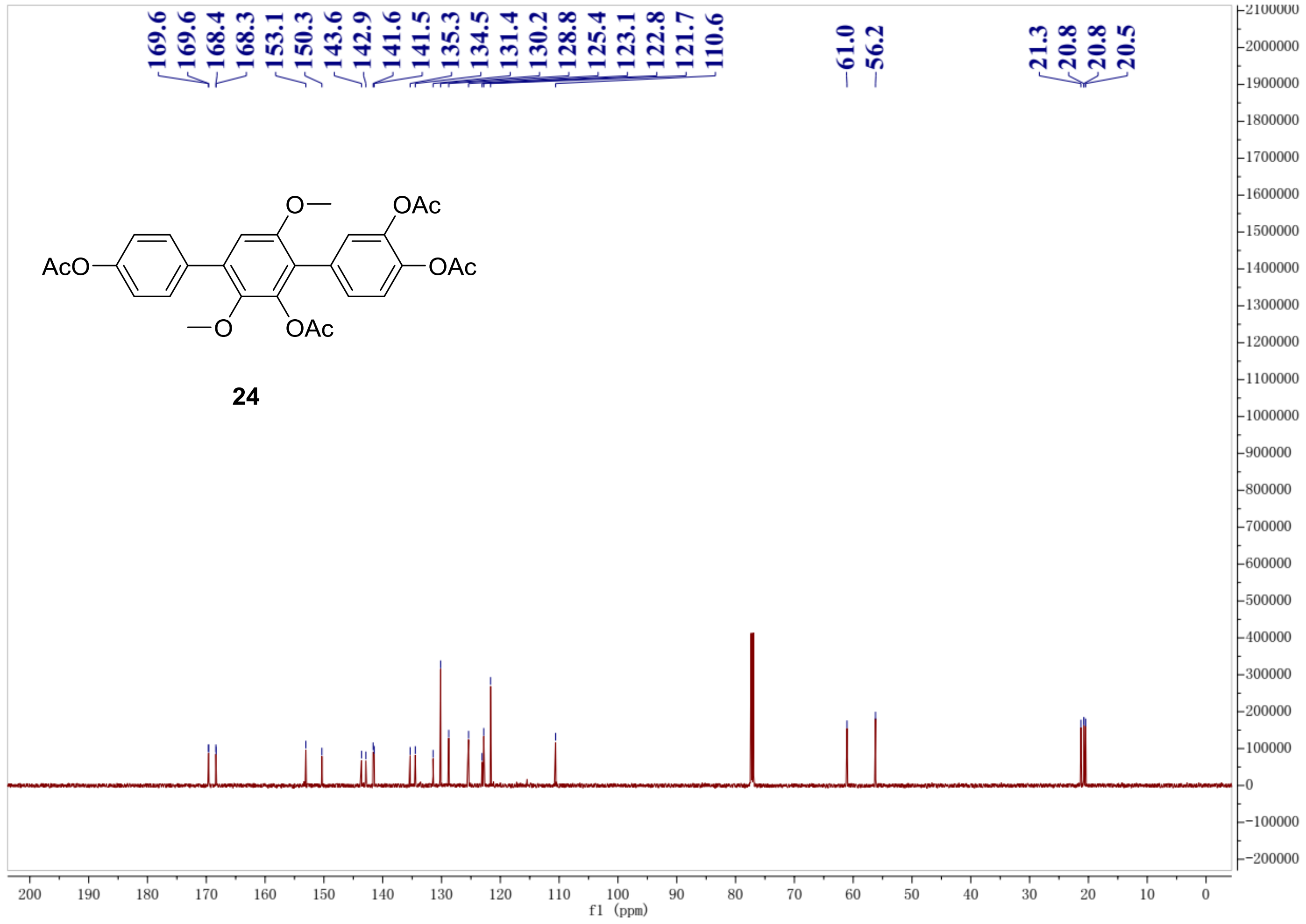

Figure S33. HRESIMS spectrum of compound **25**

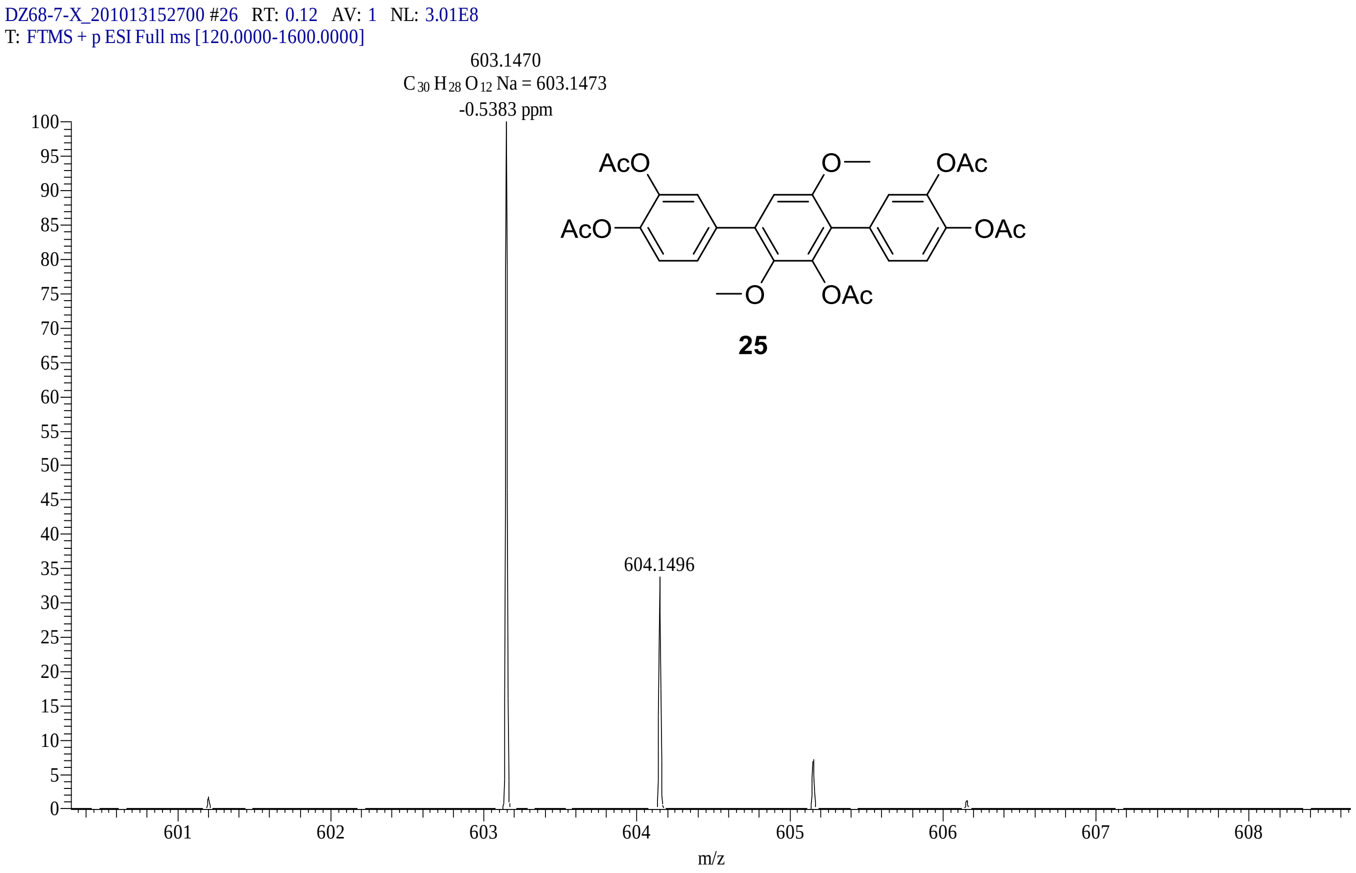

Figure S34. ^{1}H-NMR spectrum of compound **25** in $CDCl_3$

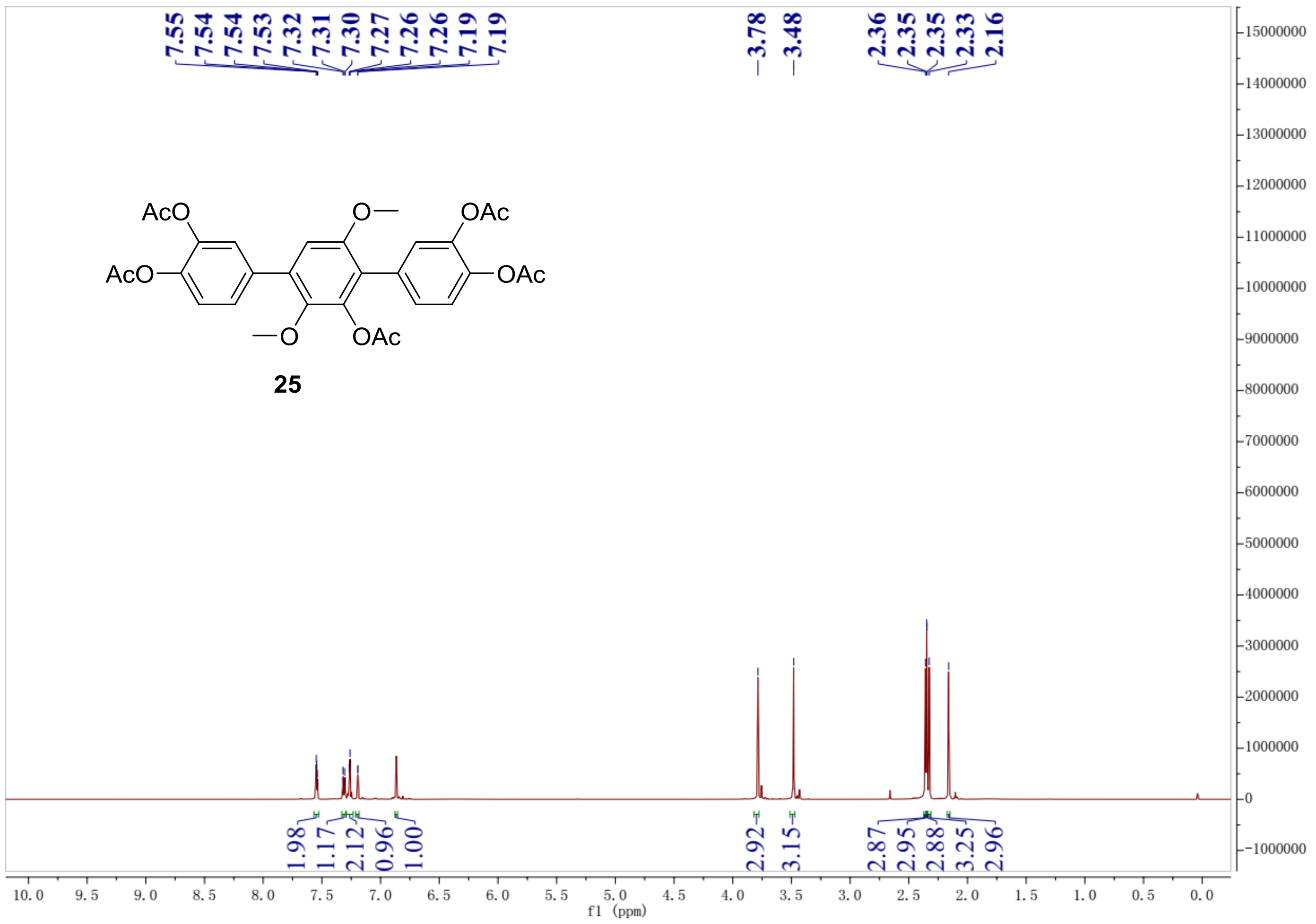

Figure S35. ^{13}C-NMR spectrum of compound **25** in $CDCl_3$

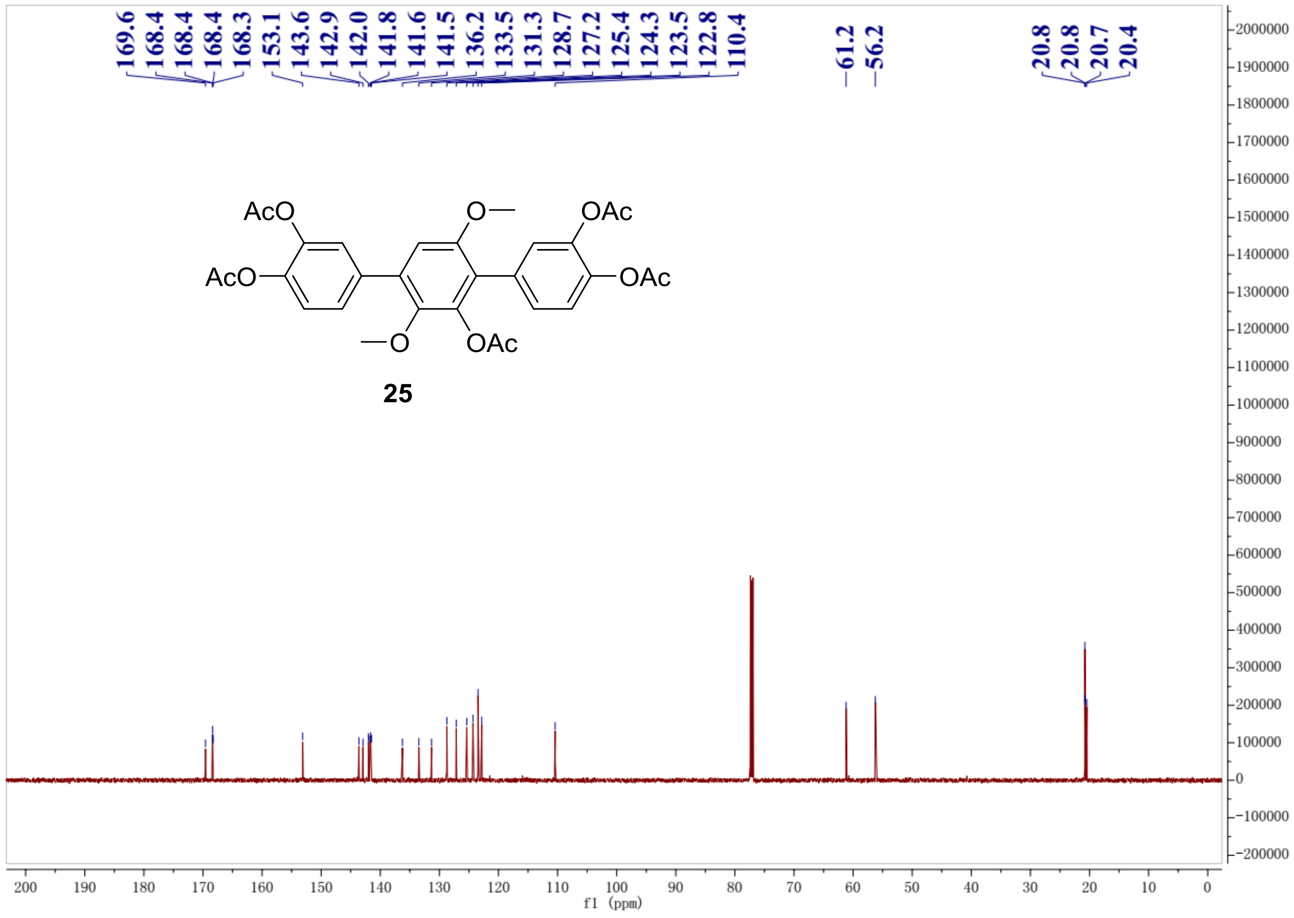

Figure S36. ORAC curves of compounds **1**–**25** and trolox

a. Testing concentration: 6.25 μM

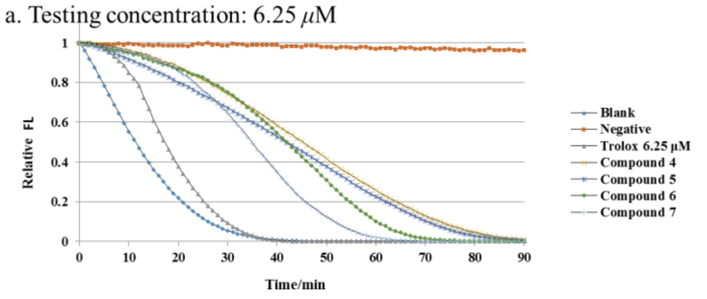

b. Testing concentration: 12.5 μM

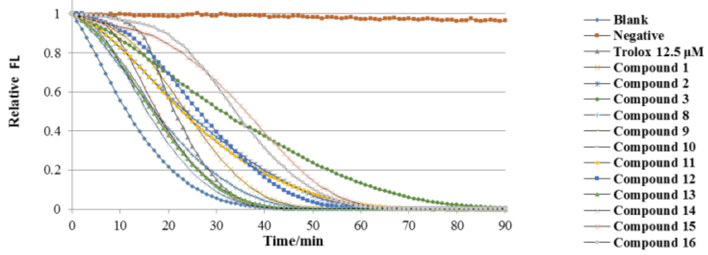

c. Testing concentration: 25 μM

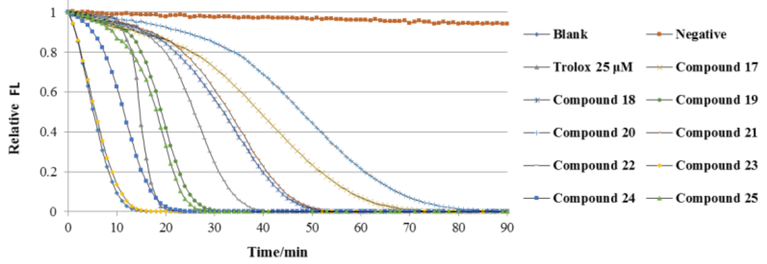

Figure S37. HPLC detection for transformation of compound **6** to compound **15** in different conditions and reaction time

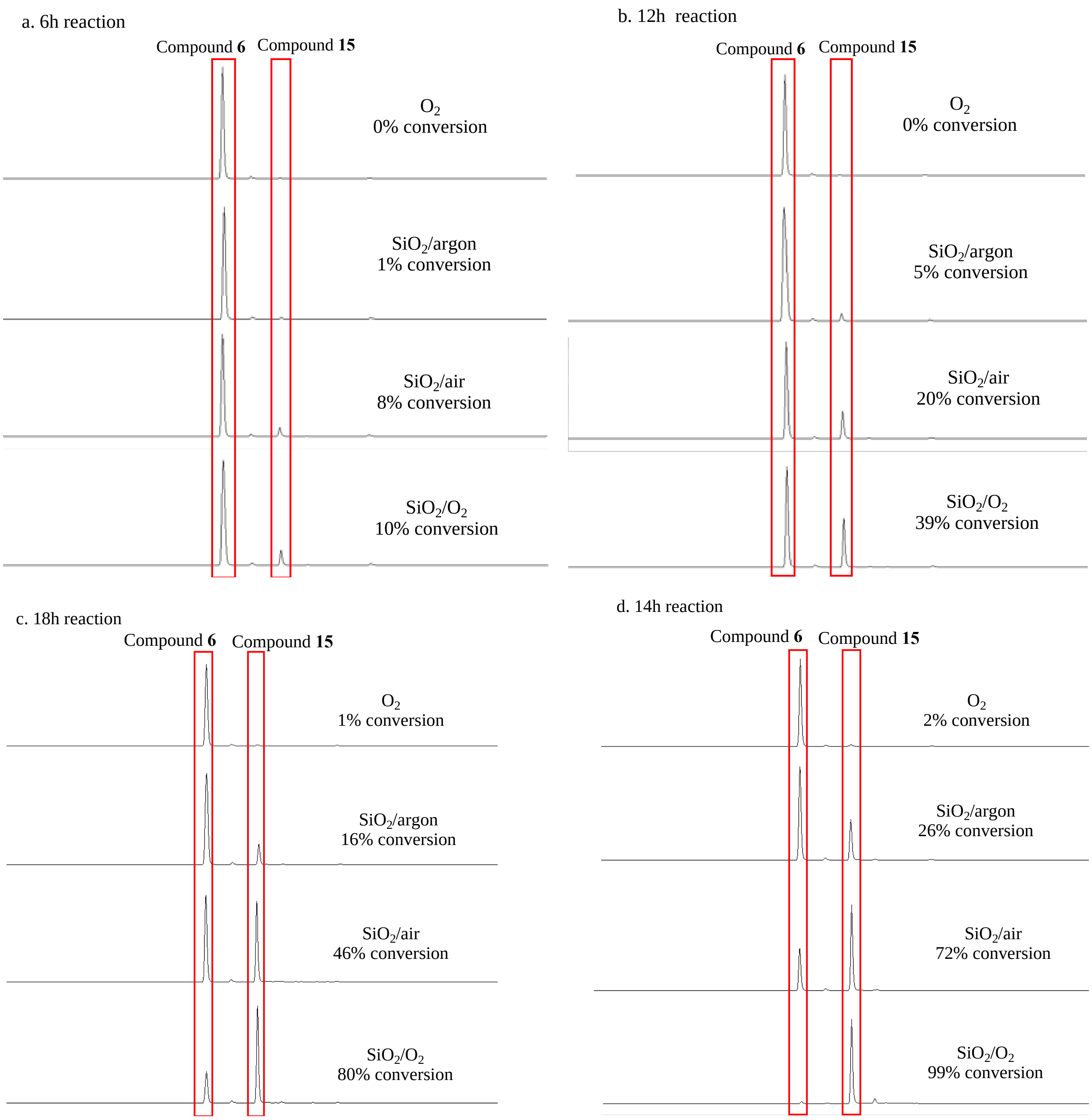

Supplement: Supplementary file 1 [file Data_Sheet_1.pdf]
